# Supplementary material for: A genome-wide association study in 10,000 individuals links plasma N-glycome to liver disease and anti-inflammatory proteins
Source: Nat Commun. 2025 Jul 1;16:5525. doi: 10.1038/s41467-025-60431-y (PMC12218978; doi:10.1038/s41467-025-60431-y)
Supplement: Supplementary file 1 — Supplementary Information [file 41467_2025_60431_MOESM1_ESM.pdf]

A genome-wide association study in 10,000 individuals links plasma N-glycome to liver disease and anti-inflammatory proteins

Sodbo Sharapov[1], Anna Timoshchuk[1], Olga Zaytseva[2], Denis Maslov[1], Anna Soplenkova[1], Elizaveta E. Elgaeva[3,4], Evgeny S. Tiys[3], Massimo Mangino[5,6], Clemens Wittenbecher[7], Lennart Karssen[8], Maria Timofeeva[9,10], Arina Nostaeva[3,8], Frano Vuckovic[2], Irena Trbojević-Akmačić[2], Tamara Štambuk[2], Sofya Feoktistova[3], Nadezhda A. Potapova[11], Viktoria Voroshilova[3,12], Frances Williams[5] , Dragan Primorac[13,14,15], Jan Van Zundert[16,17], Michel Georges[18], Karsten Suhre[19], Massimo Allegri[20], Nishi Chaturvedi[21], Malcolm Dunlop[9], Matthias B. Schulze[22,23,24], Tim Spector[5], Yakov A. Tsepilov[3,25], Gordan Lauc[2,26], Yurii S. Aulchenko[1,3]

1 MSU Institute for Artificial Intelligence, Lomonosov Moscow State University, Moscow, Russia;
2 Genos Glycoscience Research Laboratory, Borongajska cesta 83H, 10000 Zagreb, Croatia;
3 Institute of Cytology and Genetics, Novosibirsk, 630090, Russia;
4 Novosibirsk State University, Novosibirsk, 630090, Russia;
5 Department of Twin Research and Genetic Epidemiology, School of Life Course Sciences, King's College London, St Thomas' Campus, Lambeth Palace Road, London, SE1 7EH, UK;
6 NIHR Biomedical Research Centre at Guy's and St Thomas' Foundation Trust, London SE1 21 9RT, UK;
7 Department of Molecular Epidemiology, German Institute of Human Nutrition Potsdam-Rehbruecke, Nuthetal, Germany;
8 PolyOmica, 's-Hertogenbosch 5237 PA, Netherlands;
9 Colon Cancer Genetics Group, Cancer Research UK Scotland Centre, Institute of Genetics & Cancer, Western General Hospital, The University of Edinburgh, Edinburgh EH4 2XU, UK;
10 D-IAS, Danish Institute for Advanced Study, Department of Public Health, University of Southern Denmark, J.B. Winsløws Vej 9, DK-5000 Odense C, Denmark;
11 Engelhardt Institute of Molecular Biology, Russian Academy of Sciences, 119991 Moscow, Russia
12 Vavilov Institute of General Genetics Russian Academy of Sciences, 119991 Moscow, Russia;
13 St. Catherine Specialty Hospital, Ulica Kneza Branimira 71E, Zagreb, Croatia;
14 University of Split School of Medicine, Split 21 000, Croatia
15 University of Osijek School of Medicine, Osijek 31 000, Croatia
16 Department of Anesthesiology and Multidisciplinary Paincentre, ZOL, Genk/Lanaken, Belgium;
17 Department of Anesthesiology and Pain Medicine, Maastricht University Medical Centre, P. Debyelaan 25, Maastricht, 6229 HX, The Netherlands;
18 Unit of Animal Genomics, WELBIO, GIGA-R and Faculty of Veterinary Medicine, University of Liège, (B34) 1 Avenue de l'Hôpital, Liège 4000, Belgium;
19 Department of Physiology and Biophysics, Weill Cornell Medicine-Qatar, Education City, P.O. Box 24144 Doha, Qatar;
20 Centre Lemanique d'antalgie et neuromodulation – EHC - Morges - CH;
21 MRC Unit for Lifelong Health & Ageing University College London, London, UK;
22 Department of Molecular Epidemiology, German Institute of Human Nutrition Potsdam- Rehbruecke, 14558 Nuthetal, Germany;
23 German Center for Diabetes Research (DZD), Neuherberg, 85764, Germany;
24 Institute of Nutrition Science, University of Potsdam, Potsdam, Germany;
25 Wellcome Sanger Institute, Cambridge, CB10 1RQ, the UK
26 University of Zagreb Faculty of Pharmacy and Biochemistry, Zagreb, Croatia;

Supplementary Information contents

Supplementary Figure 1 …………………………………………………………………….3

Supplementary Figure 2 …………………………………………………………………4-13

Supplementary Figure 3 ……………………………………………………………..… 14-15

Supplementary Figure 4 …………………………………………………………..…... 16-19

Supplementary Figure 5 ………………………………………………………………….. 20

Supplementary Figure 6 ………………………………………………………………...…21

Supplementary Figure 7 ……………………………………………………………………...22

Supplementary Figure 8 ……………………………………………………………………...23

Supplementary Figure 9 ………………………………………………………………...…24

Supplementary Figure 10 …………………………………………………………..….. 25

Supplementary Notes ………………………………………………………………..… 26-49

References ………………………………………………………………………………….. 49-56

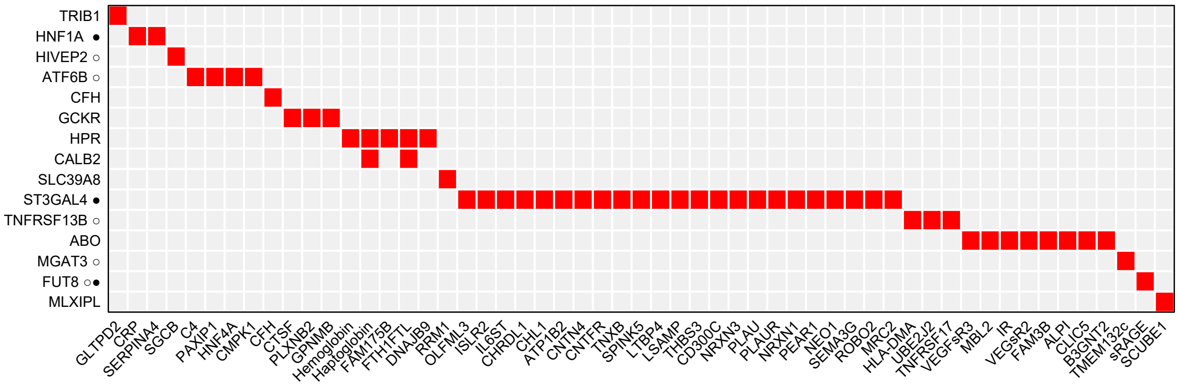

Supplementary Figure 1. Colocalisation results between glyQTLs and trans- pQTLs ($P_{adjSMR} < 0.05$ and $P_{HEIDI} >= 0.05$). Y axis – glyQTLs, X axis – trans-pQTLs (with the exception of HPT, CFAH, C4 which are cis-pQTLs)

rs16830188_1:25298841_PGP82

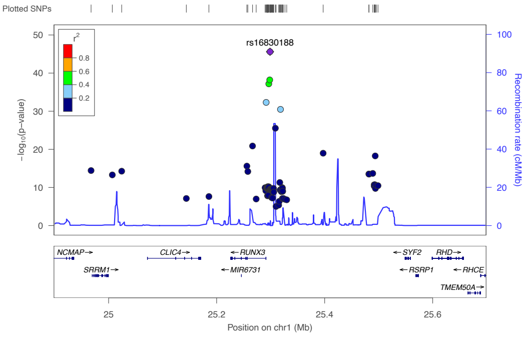

rs12738964_1:93274450_PGP102

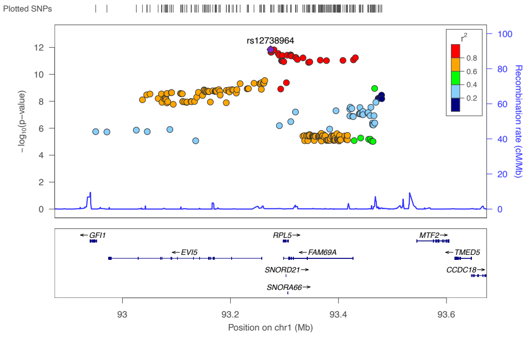

rs34881159_1:161590503_PGP6

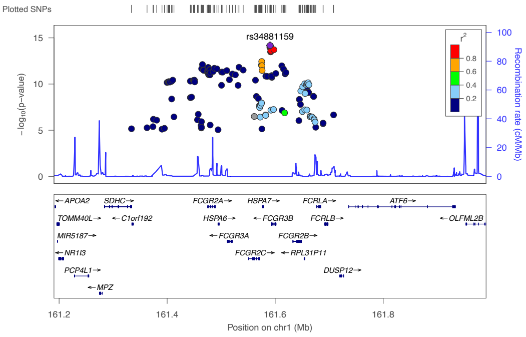

rs1260326_2:27730940_PGP102

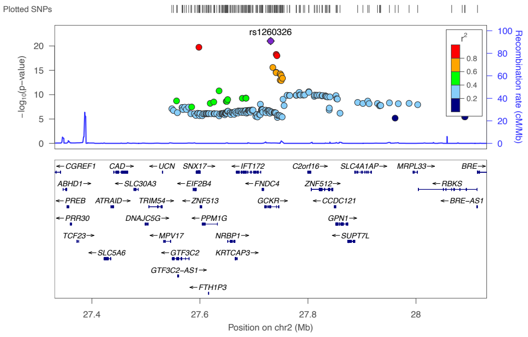

rs1257218_2:135014685_PGP103

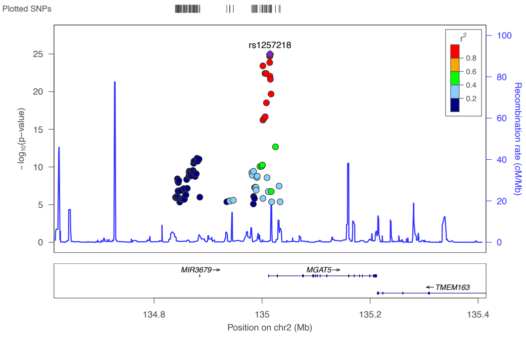

rs9842703_3:142934693_PGP57

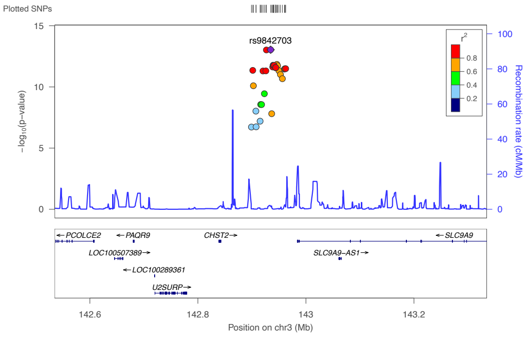

rs11710456_3:186725887_PGP41

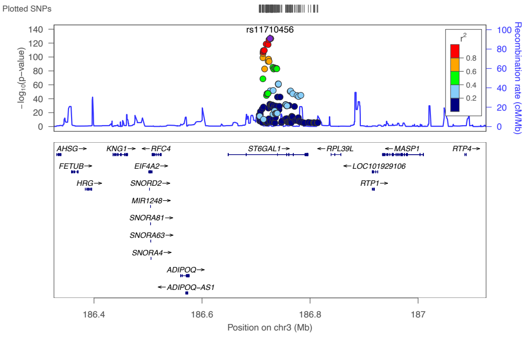

rs3774964_4:103519487_PGP70

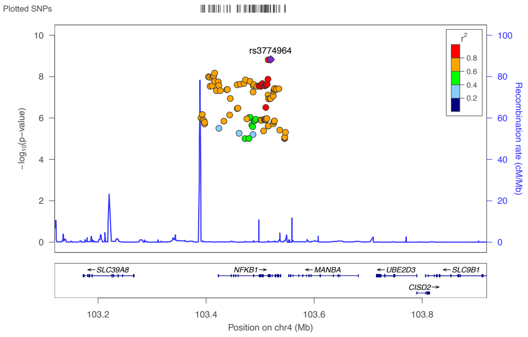

rs7705720_5:95280033_PGP41

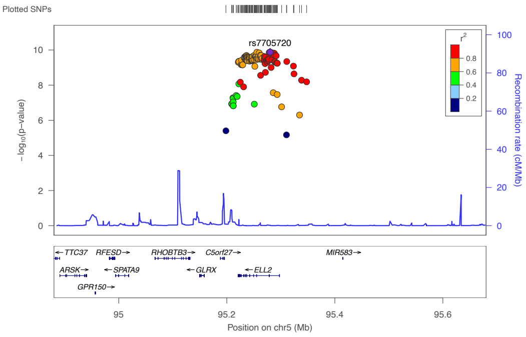

rs76360119_5:179183793_PGP23

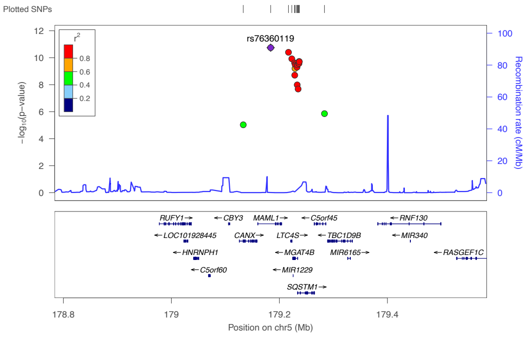

rs8283_6:32083300_PGP18

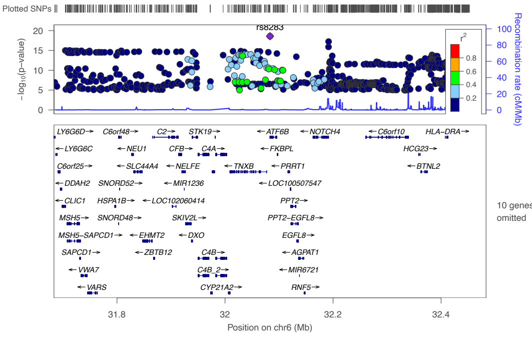

rs9352006_6:74238815_PGP72

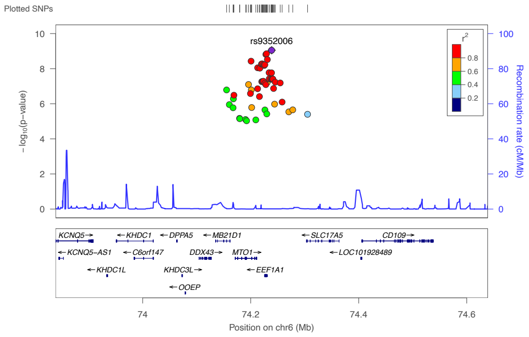

rs4543384_6:139629524_PGP63

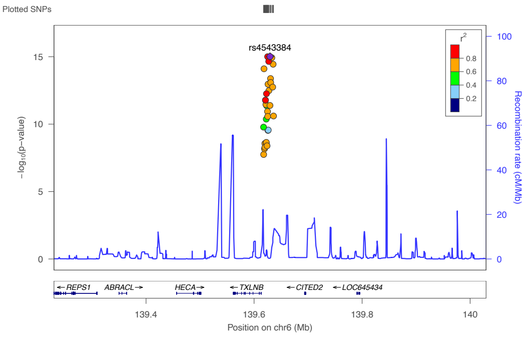

rs7758383_6:143169723_PGP67

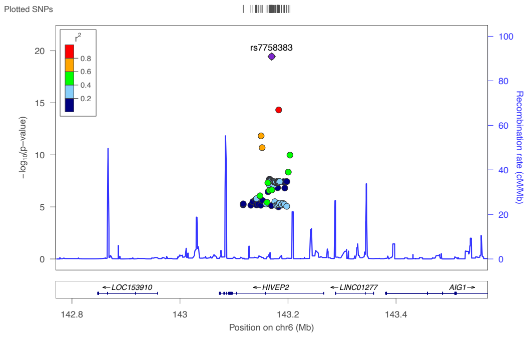

rs7804185_7:50353144_PGP60

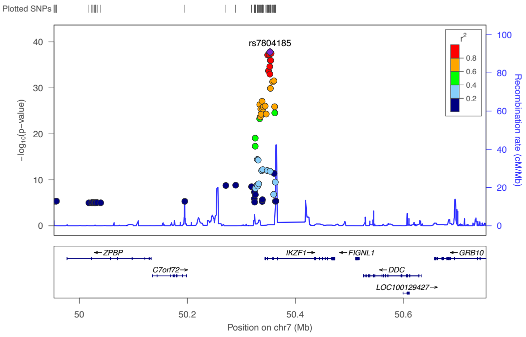

rs34166762_7:73018524_PGP22

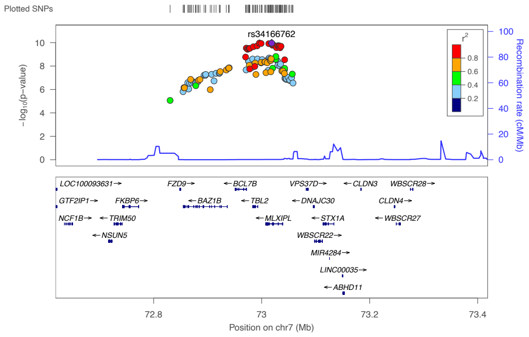

rs7781265_7:150950940_PGP74

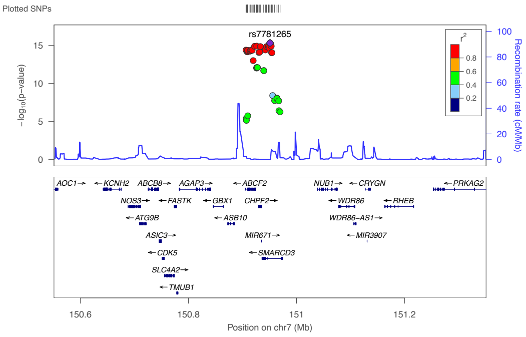

rs28601761_8:126500031_PGP112

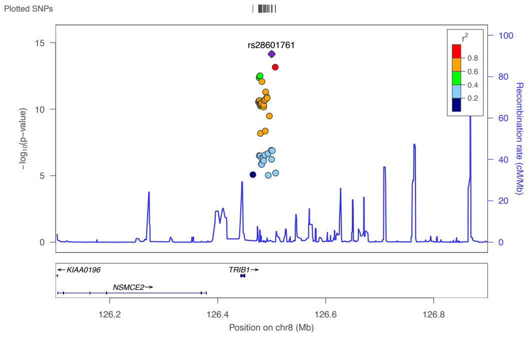

rs13297246_9:33128617_PGP67

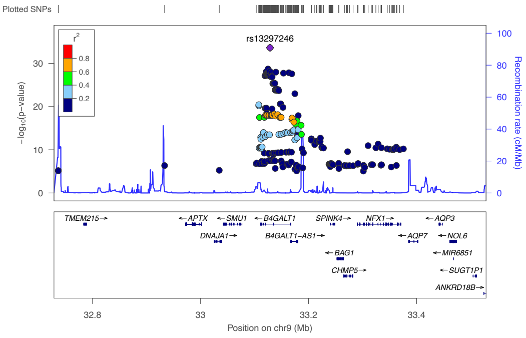

rs7866188_9:94681588_PGP114

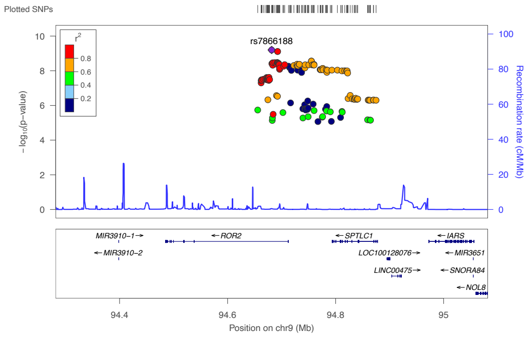

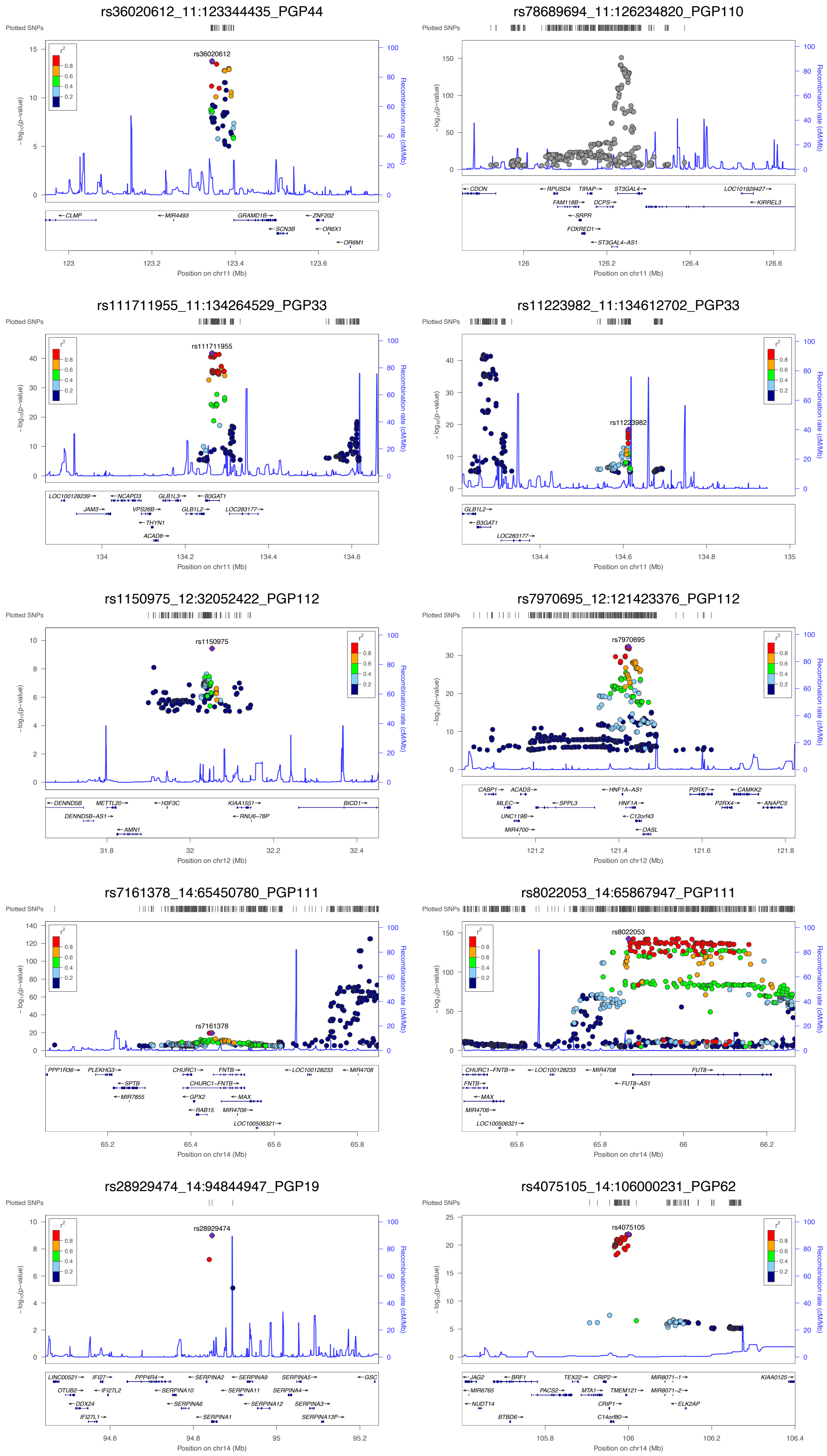
rs36020612_11:123344435_PGP44
Position on chr11 (Mb)
rs78689694_11:126234820_PGP110
Position on chr11 (Mb)
rs111711955_11:134264529_PGP33
Position on chr11 (Mb)
rs11223982_11:134612702_PGP33
Position on chr11 (Mb)
rs1150975_12:32052422_PGP112
Position on chr12 (Mb)
rs7970695_12:121423376_PGP112
Position on chr12 (Mb)
rs7161378_14:65450780_PGP111
Position on chr14 (Mb)
rs8022053_14:65867947_PGP111
Position on chr14 (Mb)
rs28929474_14:94844947_PGP19
Position on chr14 (Mb)
rs4075105_14:106000231_PGP62
Position on chr14 (Mb)
Plotted SNPs
Recombination rate (cM/Mb)

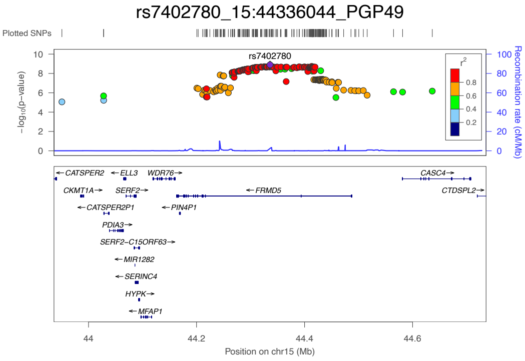
rs7402780_15:44336044_PGP49
Plotted SNPs
rs7402780
-log10(p-value)
Recombination rate (cM/Mb)
CATSPER2
ELL3
WDR76
CASC4
CKMT1A
SERF2
FRMD5
CTDSPL2
CATSPER2P1
PIN4P1
PDIA3
SERF2-C15ORF63
MIR1282
SERINC4
HYPK
MFAP1
Position on chr15 (Mb)

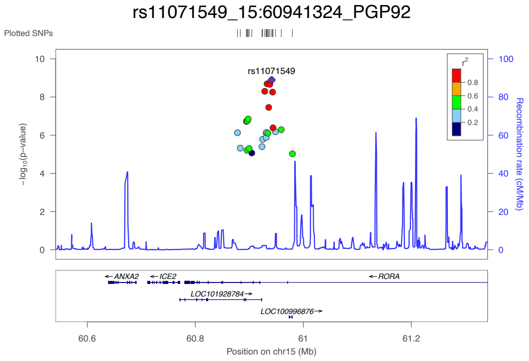
rs11071549_15:60941324_PGP92
Plotted SNPs
rs11071549
-log10(p-value)
Recombination rate (cM/Mb)
ANXA2
ICE2
RORA
LOC101928784
LOC100996876
Position on chr15 (Mb)

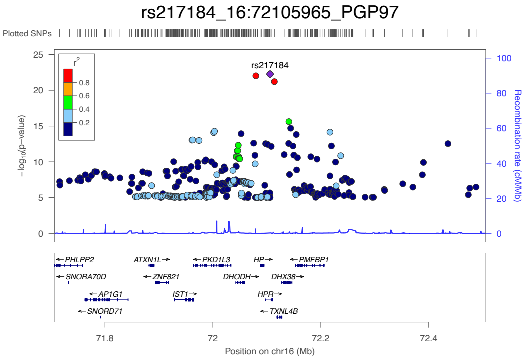
rs217184_16:72105965_PGP97
Plotted SNPs
rs217184
-log10(p-value)
Recombination rate (cM/Mb)
PHLPP2
ATXN1L
PKD1L3
HP
PMFBP1
SNORA70D
ZNF821
DHODH
DHX38
AP1G1
IST1
HPR
SNORD71
TXNL4B
Position on chr16 (Mb)

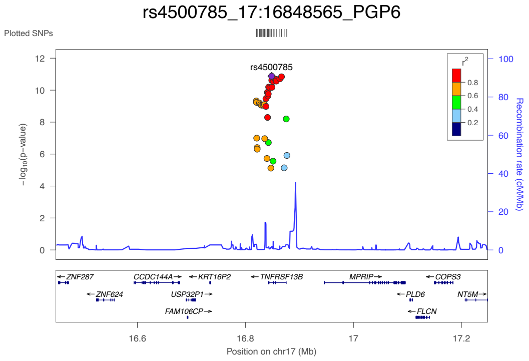
rs4500785_17:16848565_PGP6
Plotted SNPs
rs4500785
-log10(p-value)
Recombination rate (cM/Mb)
ZNF287
CCDC144A
KRT16P2
TNFRSF13B
MPRIP
COPS3
ZNF624
USP32P1
PLD6
NT5M
FAM106CP
FLCN
Position on chr17 (Mb)

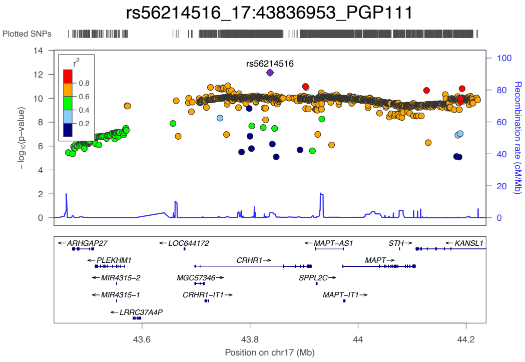
rs56214516_17:43836953_PGP111
Plotted SNPs
rs56214516
-log10(p-value)
Recombination rate (cM/Mb)
ARHGAP27
LOC644172
MAPT-AS1
STH
KANSL1
PLEKHM1
CRHR1
MAPT
MIR4315-2
MGC57346
SPPL2C
MIR4315-1
CRHR1-IT1
MAPT-IT1
LRRC37A4P
Position on chr17 (Mb)

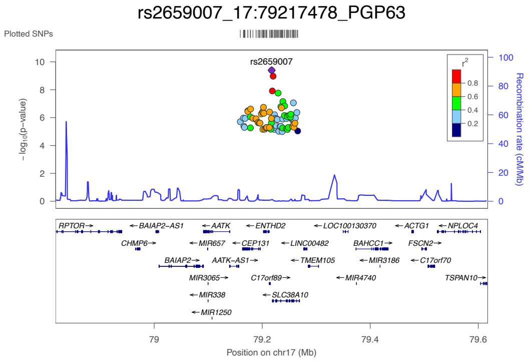
rs2659007_17:79217478_PGP63
Plotted SNPs
rs2659007
-log10(p-value)
Recombination rate (cM/Mb)
RPTOR
BAIAP2-AS1
AATK
ENTHD2
LOC100130370
ACTG1
NPLOC4
CHMP6
MIR657
CEP131
LINC00482
BAHCC1
FSCN2
BAIAP2
AATK-AS1
TMEM105
MIR3186
C17orf70
MIR3065
C17orf89
MIR4740
TSPAN10
MIR338
SLC38A10
MIR1250
Position on chr17 (Mb)

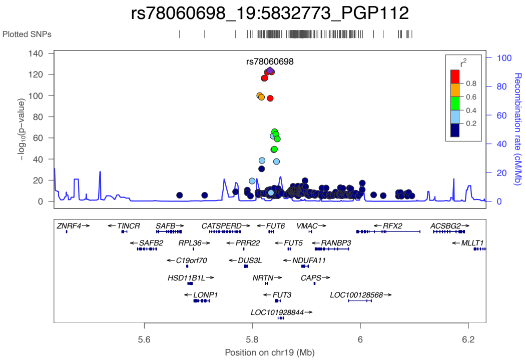
rs78060698_19:5832773_PGP112
Plotted SNPs
rs78060698
-log10(p-value)
Recombination rate (cM/Mb)
ZNRF4
TINCR
SAFB
CATSPERD
FUT6
VMAC
RFX2
ACSBG2
SAFB2
RPL36
PRR22
FUT5
RANBP3
MLLT1
C19orf70
DUS3L
NDUFA11
HSD11B1L
NRTN
CAPS
LONP1
FUT3
LOC100128568
LOC101928844
Position on chr19 (Mb)

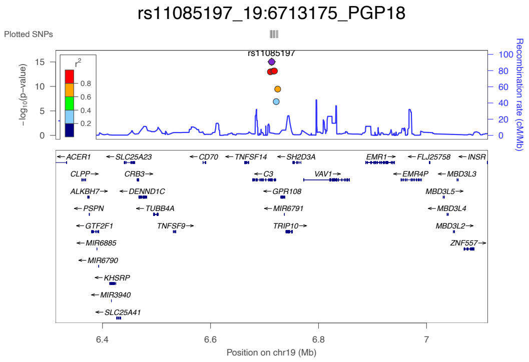
rs11085197_19:6713175_PGP18
Plotted SNPs
rs11085197
-log10(p-value)
Recombination rate (cM/Mb)
ACER1
SLC25A23
CD70
TNFSF14
SH2D3A
EMR1
FLJ25758
INSR
CLPP
CRB3
C3
VAV1
EMR4P
MBD3L3
ALKBH7
DENND1C
GPR108
MBD3L5
PSPN
TUBB4A
MIR6791
MBD3L4
GTF2F1
TNFSF9
TRIP10
MBD3L2
MIR6885
ZNF557
MIR6790
KHSRP
MIR3940
SLC25A41
Position on chr19 (Mb)

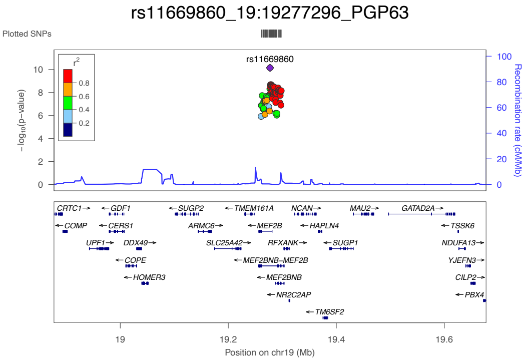
rs11669860_19:19277296_PGP63
Plotted SNPs
rs11669860
-log10(p-value)
Recombination rate (cM/Mb)
CRTC1
GDF1
SUGP2
TMEM161A
NCAN
MAU2
GATAD2A
COMP
CERS1
ARMC6
MEF2B
HAPLN4
TSSK6
UPF1
DDX49
SLC25A42
RFXANK
SUGP1
NDUFA13
COPE
MEF2BNB-MEF2B
YJEFN3
HOMER3
MEF2BNB
CILP2
NR2C2AP
PBX4
TM6SF2
Position on chr19 (Mb)

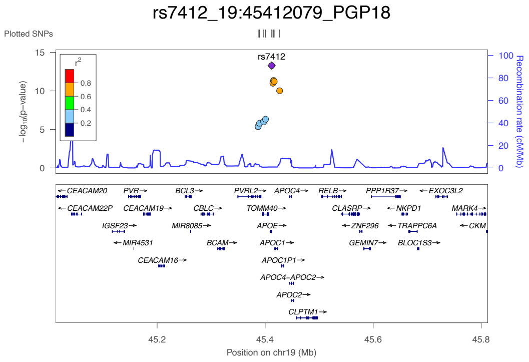
rs7412_19:45412079_PGP18
Plotted SNPs
rs7412
-log10(p-value)
Recombination rate (cM/Mb)
CEACAM20
PVR
BCL3
PVRL2
APOC4
RELB
PPP1R37
EXOC3L2
CEACAM22P
CEACAM19
CBLC
TOMM40
CLASRP
NKPD1
MARK4
IGSF23
MIR8085
APOE
ZNF296
TRAPPC6A
CKM
MIR4531
BCAM
APOC1
GEMIN7
BLOC1S3
CEACAM16
APOC1P1
APOC4-APOC2
APOC2
CLPTM1
Position on chr19 (Mb)

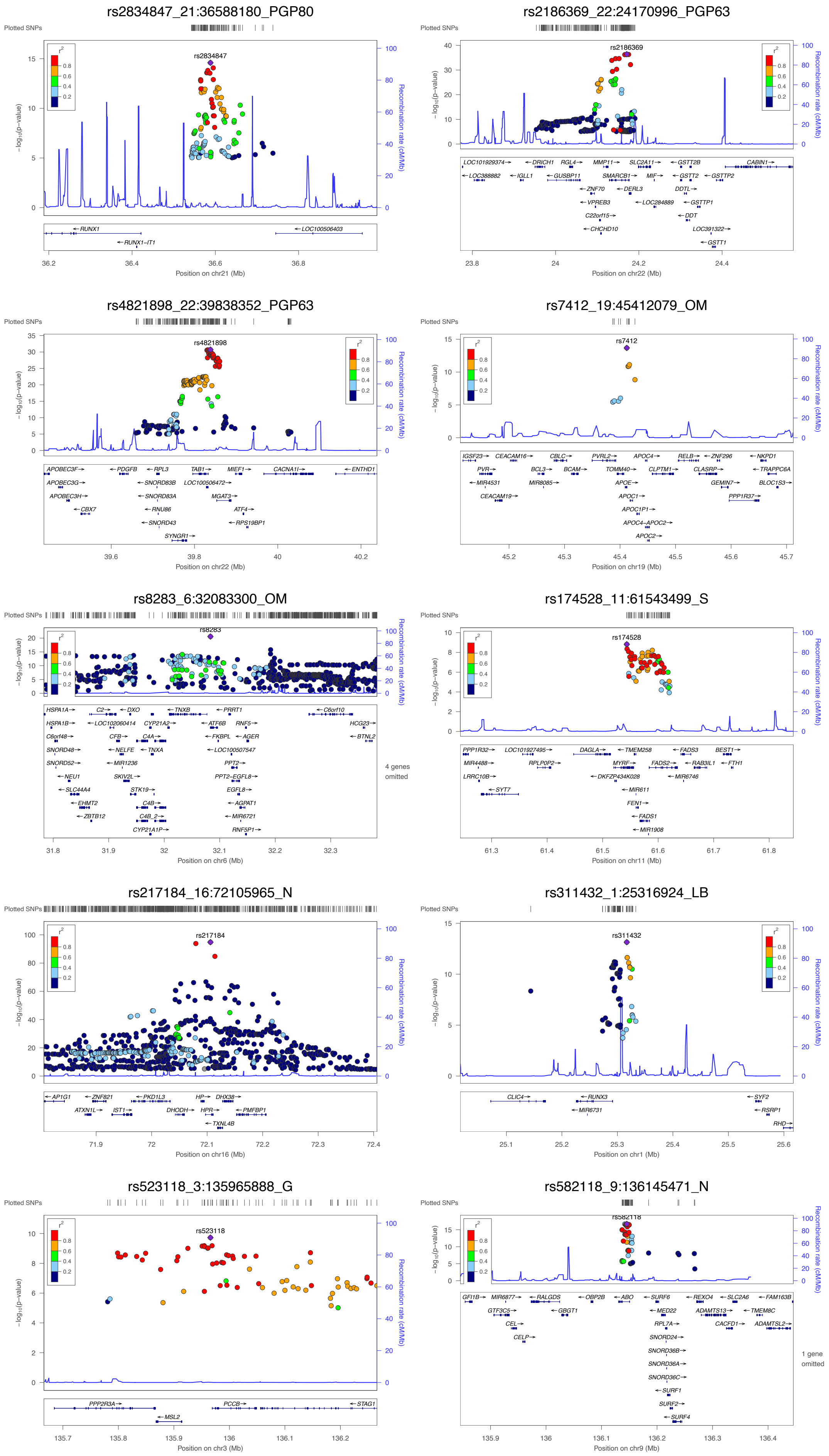
rs2834847_21:36588180_PGP80
rs2186369_22:24170996_PGP63
rs4821898_22:39838352_PGP63
rs7412_19:45412079_OM
rs8283_6:32083300_OM
rs174528_11:61543499_S
rs217184_16:72105965_N
rs311432_1:25316924_LB
rs523118_3:135965888_G
rs582118_9:136145471_N
Plotted SNPs
Recombination rate (cM/Mb)
Position on chr21 (Mb)
Position on chr22 (Mb)
Position on chr19 (Mb)
Position on chr6 (Mb)
Position on chr11 (Mb)
Position on chr16 (Mb)
Position on chr1 (Mb)
Position on chr3 (Mb)
Position on chr9 (Mb)
4 genes omitted
1 gene omitted

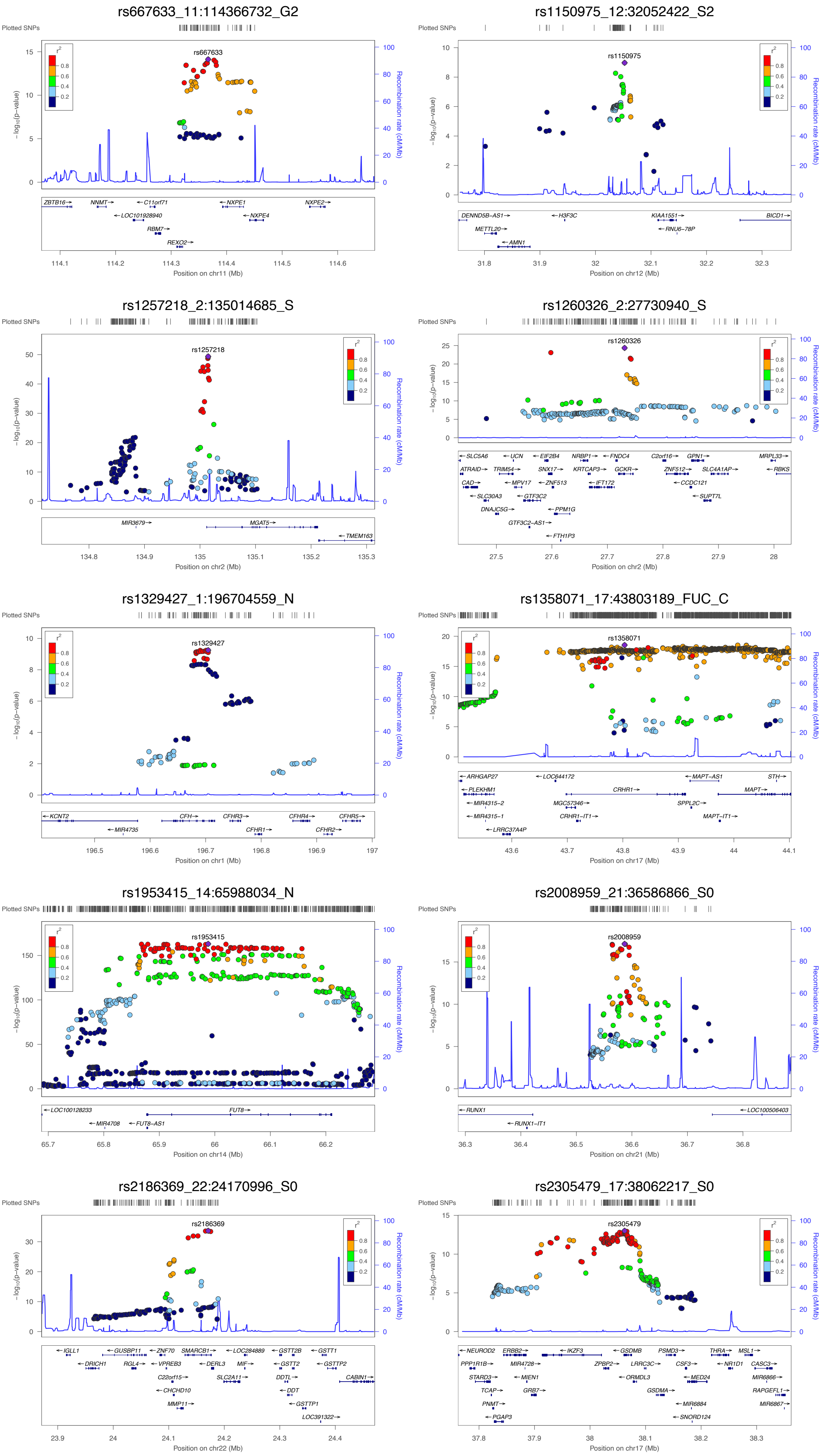
rs667633_11:114366732_G2
Plotted SNPs
rs667633
− log10(p−value)
Recombination rate (cM/Mb)
ZBTB16 NNMT C11orf71 NXPE1 NXPE2 LOC101928940 NXPE4 RBM7 REXO2
Position on chr11 (Mb)
rs1150975_12:32052422_S2
rs1150975
DENND5B−AS1 H3F3C KIAA1551 BICD1 METTL20 RNU6−78P AMN1
Position on chr12 (Mb)
rs1257218_2:135014685_S
rs1257218
MIR3679 MGAT5 TMEM163
Position on chr2 (Mb)
rs1260326_2:27730940_S
rs1260326
SLC5A6 UCN EIF2B4 NRBP1 FNDC4 C2orf16 GPN1 MRPL33 ATRAID TRIM54 SNX17 KRTCAP3 GCKR ZNF512 SLC4A1AP RBKS CAD MPV17 ZNF513 IFT172 CCDC121 SLC30A3 GTF3C2 SUPT7L DNAJC5G PPM1G GTF3C2−AS1 FTH1P3
Position on chr2 (Mb)
rs1329427_1:196704559_N
rs1329427
KCNT2 CFH CFHR3 CFHR4 CFHR5 MIR4735 CFHR1 CFHR2
Position on chr1 (Mb)
rs1358071_17:43803189_FUC_C
rs1358071
ARHGAP27 LOC644172 MAPT−AS1 STH PLEKHM1 CRHR1 MAPT MIR4315−2 MGC57346 SPPL2C MIR4315−1 CRHR1−IT1 MAPT−IT1 LRRC37A4P
Position on chr17 (Mb)
rs1953415_14:65988034_N
rs1953415
LOC100128233 FUT8 MIR4708 FUT8−AS1
Position on chr14 (Mb)
rs2008959_21:36586866_S0
rs2008959
RUNX1 LOC100506403 RUNX1−IT1
Position on chr21 (Mb)
rs2186369_22:24170996_S0
rs2186369
IGLL1 GUSBP11 ZNF70 SMARCB1 LOC284889 GSTT2B GSTT1 DRICH1 RGL4 VPREB3 DERL3 MIF GSTT2 GSTTP2 C22orf15 SLC2A11 DDTL CABIN1 CHCHD10 DDT MMP11 GSTTP1 LOC391322
Position on chr22 (Mb)
rs2305479_17:38062217_S0
rs2305479
NEUROD2 ERBB2 IKZF3 GSDMB PSMD3 THRA MSL1 PPP1R1B MIR4728 ZPBP2 LRRC3C CSF3 NR1D1 CASC3 STARD3 MIEN1 ORMDL3 MED24 MIR6866 TCAP GRB7 GSDMA RAPGEFL1 PNMT MIR6884 MIR6867 PGAP3 SNORD124
Position on chr17 (Mb)

rs2470750_3:98690592_HB

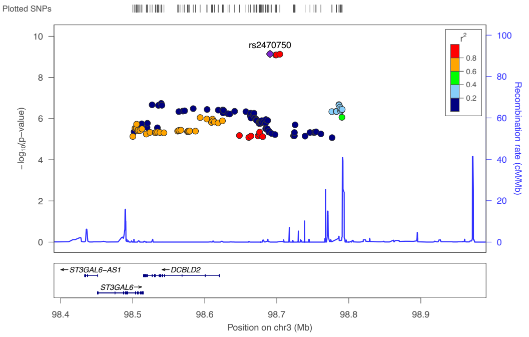

rs2497318_10:94432000_G1

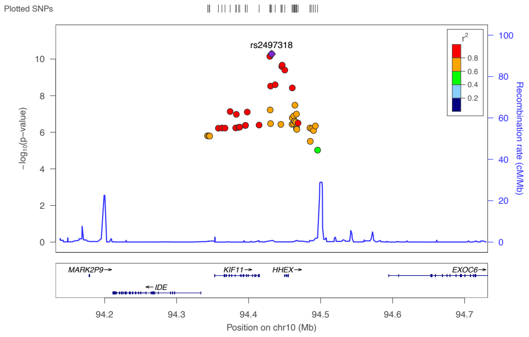

rs2511737_8:103580527_B

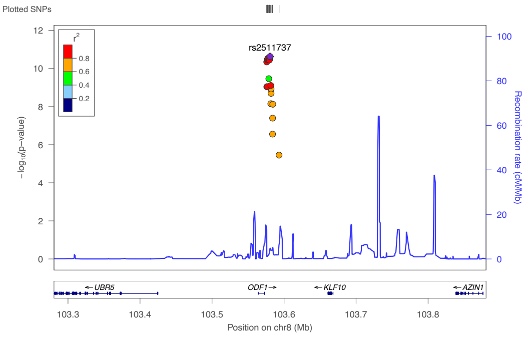

rs2618588_20:17832658_FUC_C

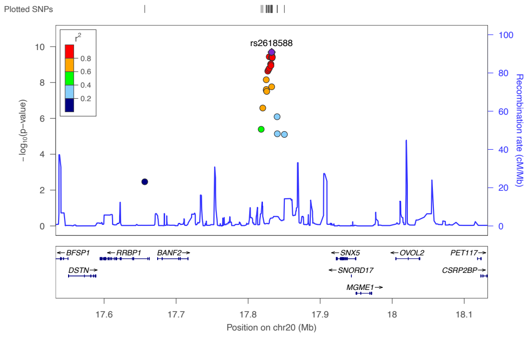

rs2659007_17:79217478_G

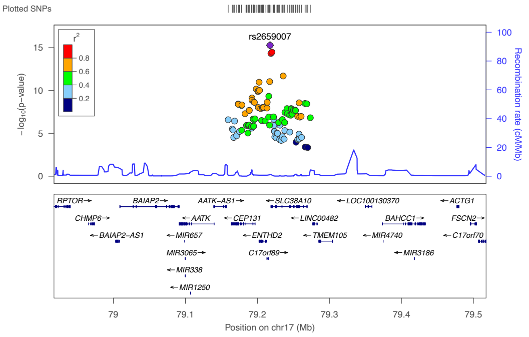

rs2881699_7:6544886_N

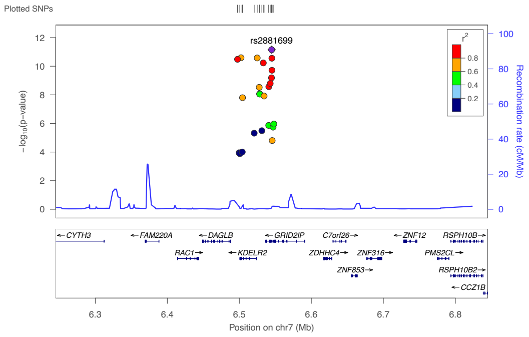

rs3777183_5:95263735_N

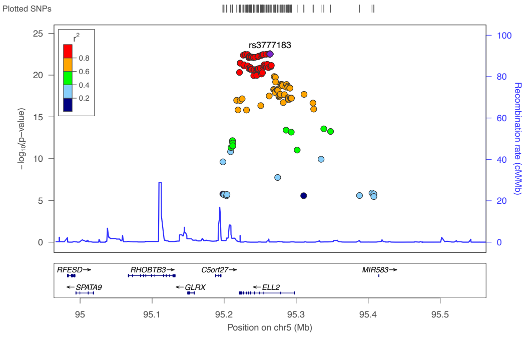

rs3795016_19:6003679_HB

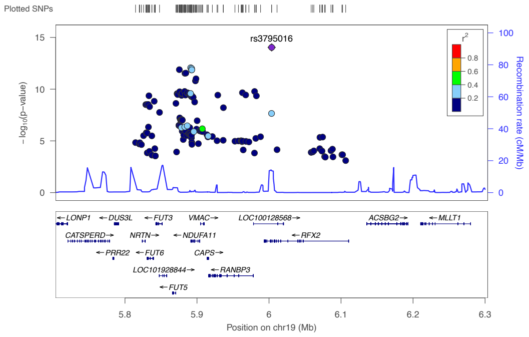

rs3822960_6:74230859_FUC_C

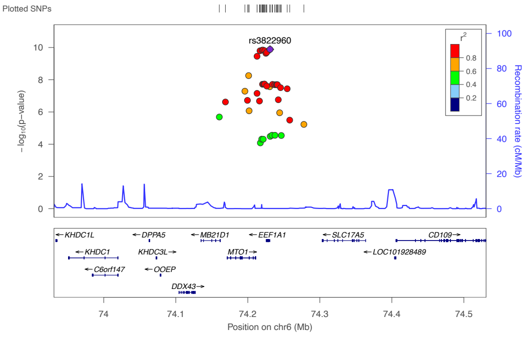

rs4543384_6:139629524_FUC_C

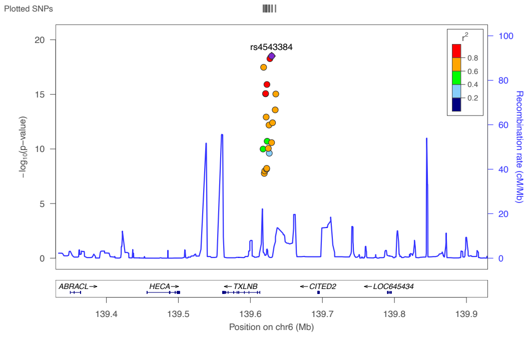

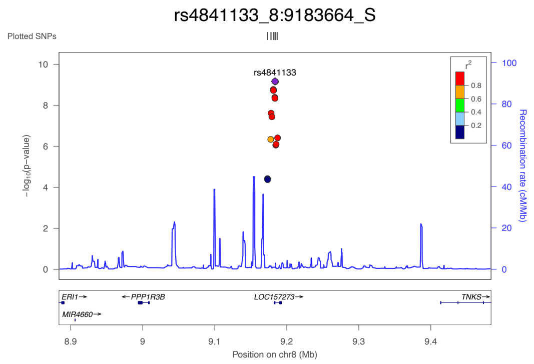
rs4841133_8:9183664_S
Plotted SNPs
rs4841133
−log10(p−value)
Recombination rate (cM/Mb)
ERI1
PPP1R3B
LOC157273
TNKS
MIR4660
Position on chr8 (Mb)
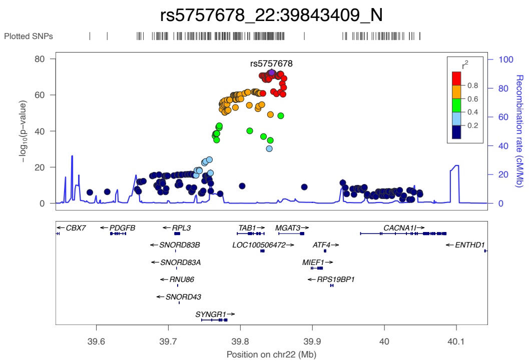
rs5757678_22:39843409_N
Plotted SNPs
rs5757678
−log10(p−value)
Recombination rate (cM/Mb)
CBX7
PDGFB
RPL3
TAB1
MGAT3
CACNA1I
SNORD83B
LOC100506472
ATF4
ENTHD1
SNORD83A
MIEF1
RNU86
RPS19BP1
SNORD43
SYNGR1
Position on chr22 (Mb)
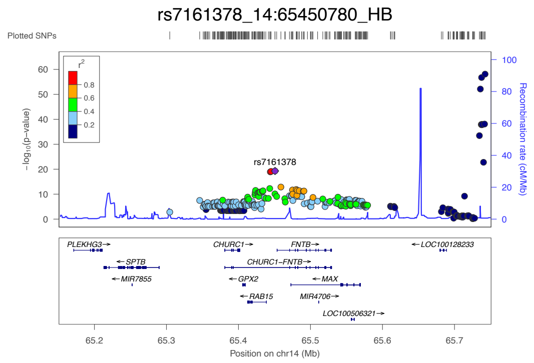
rs7161378_14:65450780_HB
Plotted SNPs
rs7161378
−log10(p−value)
Recombination rate (cM/Mb)
PLEKHG3
CHURC1
FNTB
LOC100128233
SPTB
CHURC1−FNTB
MIR7855
GPX2
MAX
RAB15
MIR4706
LOC100506321
Position on chr14 (Mb)
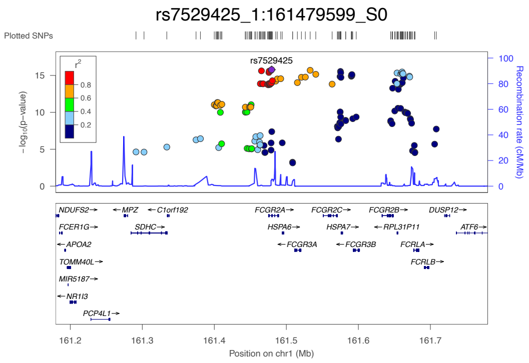
rs7529425_1:161479599_S0
Plotted SNPs
rs7529425
−log10(p−value)
Recombination rate (cM/Mb)
NDUFS2
MPZ
C1orf192
FCGR2A
FCGR2C
FCGR2B
DUSP12
FCER1G
SDHC
HSPA6
HSPA7
RPL31P11
ATF6
APOA2
FCGR3A
FCGR3B
FCRLA
TOMM40L
FCRLB
MIR5187
NR1I3
PCP4L1
Position on chr1 (Mb)
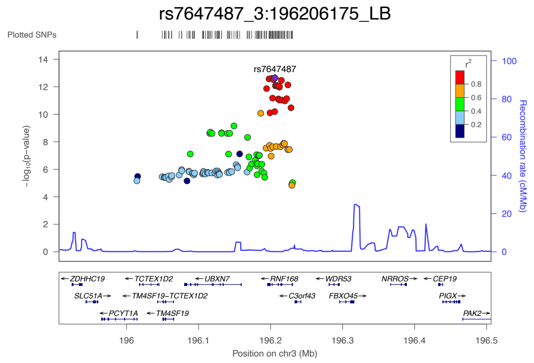
rs7647487_3:196206175_LB
Plotted SNPs
rs7647487
−log10(p−value)
Recombination rate (cM/Mb)
ZDHHC19
TCTEX1D2
UBXN7
RNF168
WDR53
NRROS
CEP19
SLC51A
TM4SF19−TCTEX1D2
C3orf43
FBXO45
PIGX
PCYT1A
TM4SF19
PAK2
Position on chr3 (Mb)
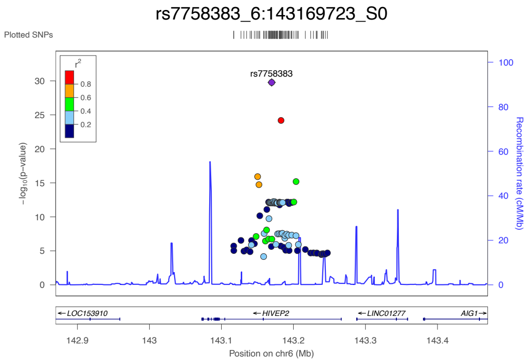
rs7758383_6:143169723_S0
Plotted SNPs
rs7758383
−log10(p−value)
Recombination rate (cM/Mb)
LOC153910
HIVEP2
LINC01277
AIG1
Position on chr6 (Mb)
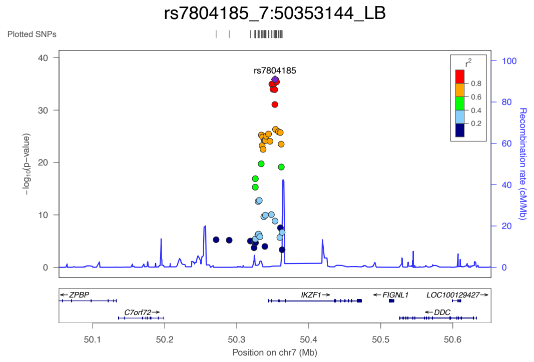
rs7804185_7:50353144_LB
Plotted SNPs
rs7804185
−log10(p−value)
Recombination rate (cM/Mb)
ZPBP
IKZF1
FIGNL1
LOC100129427
C7orf72
DDC
Position on chr7 (Mb)
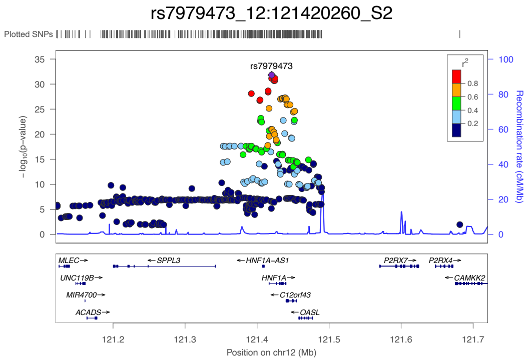
rs7979473_12:121420260_S2
Plotted SNPs
rs7979473
−log10(p−value)
Recombination rate (cM/Mb)
MLEC
SPPL3
HNF1A−AS1
P2RX7
P2RX4
UNC119B
HNF1A
CAMKK2
MIR4700
C12orf43
ACADS
OASL
Position on chr12 (Mb)
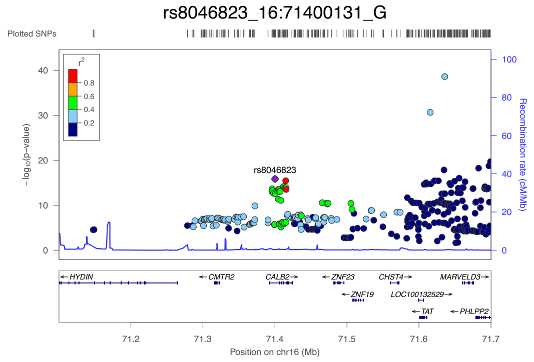
rs8046823_16:71400131_G
Plotted SNPs
rs8046823
−log10(p−value)
Recombination rate (cM/Mb)
HYDIN
CMTR2
CALB2
ZNF23
CHST4
MARVELD3
ZNF19
LOC100132529
TAT
PHLPP2
Position on chr16 (Mb)
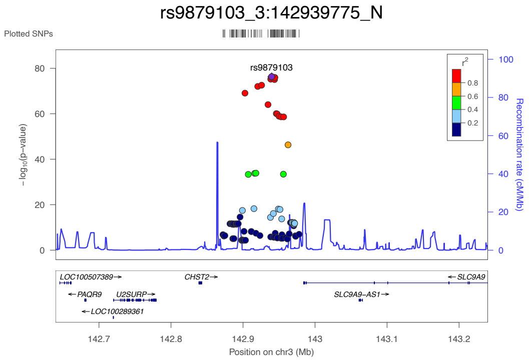
rs9879103_3:142939775_N
Plotted SNPs
rs9879103
−log10(p−value)
Recombination rate (cM/Mb)
LOC100507389
CHST2
SLC9A9
PAQR9
U2SURP
SLC9A9−AS1
LOC100289361
Position on chr3 (Mb)

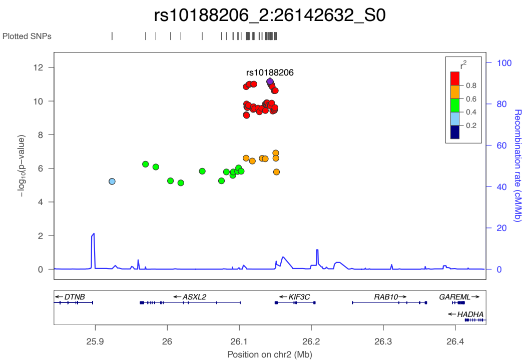
rs10188206_2:26142632_S0
Plotted SNPs
rs10188206
-log10(p-value)
Recombination rate (cM/Mb)
DTNB
ASXL2
KIF3C
RAB10
GAREML
HADHA
Position on chr2 (Mb)

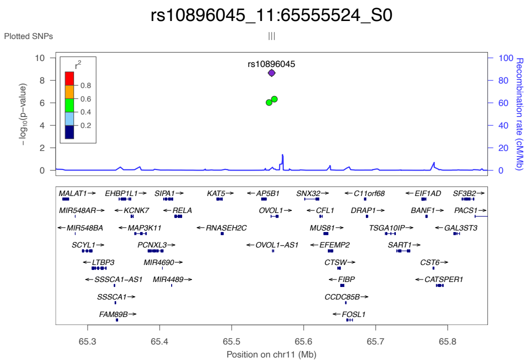
rs10896045_11:65555524_S0
Plotted SNPs
rs10896045
-log10(p-value)
Recombination rate (cM/Mb)
MALAT1
EHBP1L1
SIPA1
KAT5
AP5B1
SNX32
C11orf68
EIF1AD
SF3B2
MIR548AR
KCNK7
RELA
OVOL1
CFL1
DRAP1
BANF1
PACS1
MIR548BA
MAP3K11
RNASEH2C
MUS81
TSGA10IP
GAL3ST3
SCYL1
PCNXL3
OVOL1-AS1
EFEMP2
SART1
LTBP3
MIR4690
CTSW
CST6
SSSCA1-AS1
MIR4489
FIBP
CATSPER1
SSSCA1
CCDC85B
FAM89B
FOSL1
Position on chr11 (Mb)

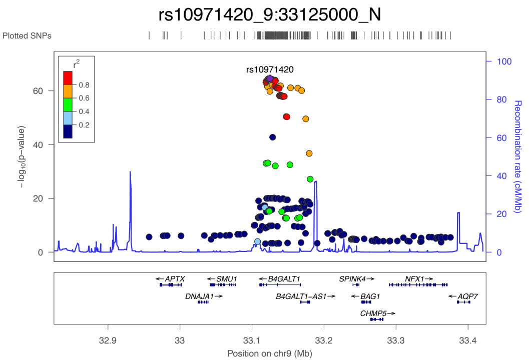
rs10971420_9:33125000_N
Plotted SNPs
rs10971420
-log10(p-value)
Recombination rate (cM/Mb)
APTX
SMU1
B4GALT1
SPINK4
NFX1
DNAJA1
B4GALT1-AS1
BAG1
AQP7
CHMP5
Position on chr9 (Mb)

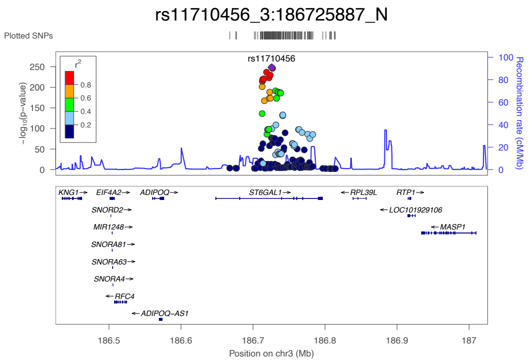
rs11710456_3:186725887_N
Plotted SNPs
rs11710456
-log10(p-value)
Recombination rate (cM/Mb)
KNG1
EIF4A2
ADIPOQ
ST6GAL1
RPL39L
RTP1
SNORD2
LOC101929106
MIR1248
MASP1
SNORA81
SNORA63
SNORA4
RFC4
ADIPOQ-AS1
Position on chr3 (Mb)

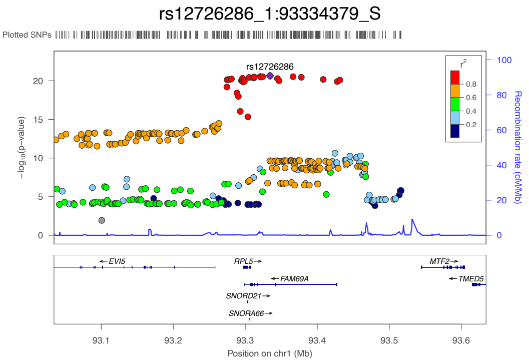
rs12726286_1:93334379_S
Plotted SNPs
rs12726286
-log10(p-value)
Recombination rate (cM/Mb)
EVI5
RPL5
MTF2
FAM69A
TMED5
SNORD21
SNORA66
Position on chr1 (Mb)

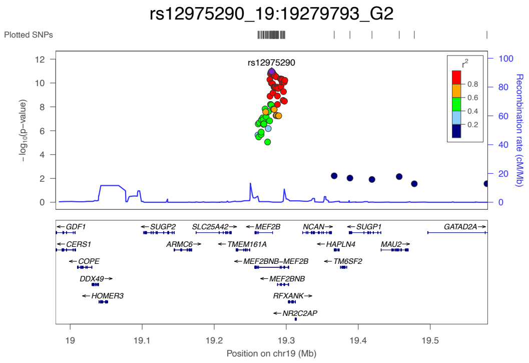
rs12975290_19:19279793_G2
Plotted SNPs
rs12975290
-log10(p-value)
Recombination rate (cM/Mb)
GDF1
SUGP2
SLC25A42
MEF2B
NCAN
SUGP1
GATAD2A
CERS1
ARMC6
TMEM161A
HAPLN4
MAU2
COPE
MEF2BNB-MEF2B
TM6SF2
DDX49
MEF2BNB
HOMER3
RFXANK
NR2C2AP
Position on chr19 (Mb)

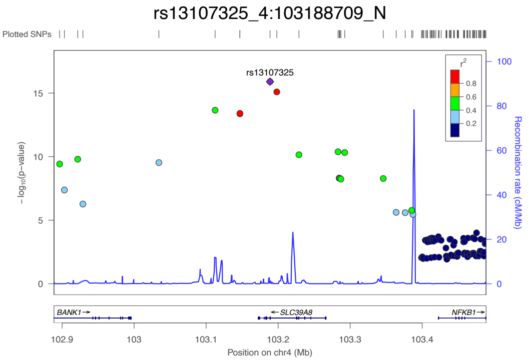
rs13107325_4:103188709_N
Plotted SNPs
rs13107325
-log10(p-value)
Recombination rate (cM/Mb)
BANK1
SLC39A8
NFKB1
Position on chr4 (Mb)

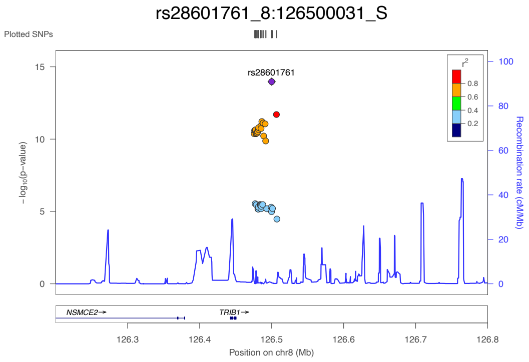
rs28601761_8:126500031_S
Plotted SNPs
rs28601761
-log10(p-value)
Recombination rate (cM/Mb)
NSMCE2
TRIB1
Position on chr8 (Mb)

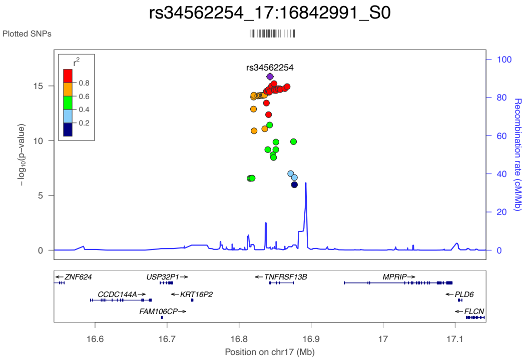
rs34562254_17:16842991_S0
Plotted SNPs
rs34562254
-log10(p-value)
Recombination rate (cM/Mb)
ZNF624
USP32P1
TNFRSF13B
MPRIP
CCDC144A
KRT16P2
PLD6
FAM106CP
FLCN
Position on chr17 (Mb)

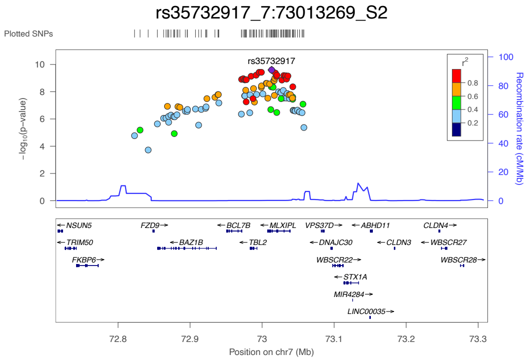
rs35732917_7:73013269_S2
Plotted SNPs
rs35732917
-log10(p-value)
Recombination rate (cM/Mb)
NSUN5
FZD9
BCL7B
MLXIPL
VPS37D
ABHD11
CLDN4
TRIM50
BAZ1B
TBL2
DNAJC30
CLDN3
WBSCR27
FKBP6
WBSCR22
WBSCR28
STX1A
MIR4284
LINC00035
Position on chr7 (Mb)

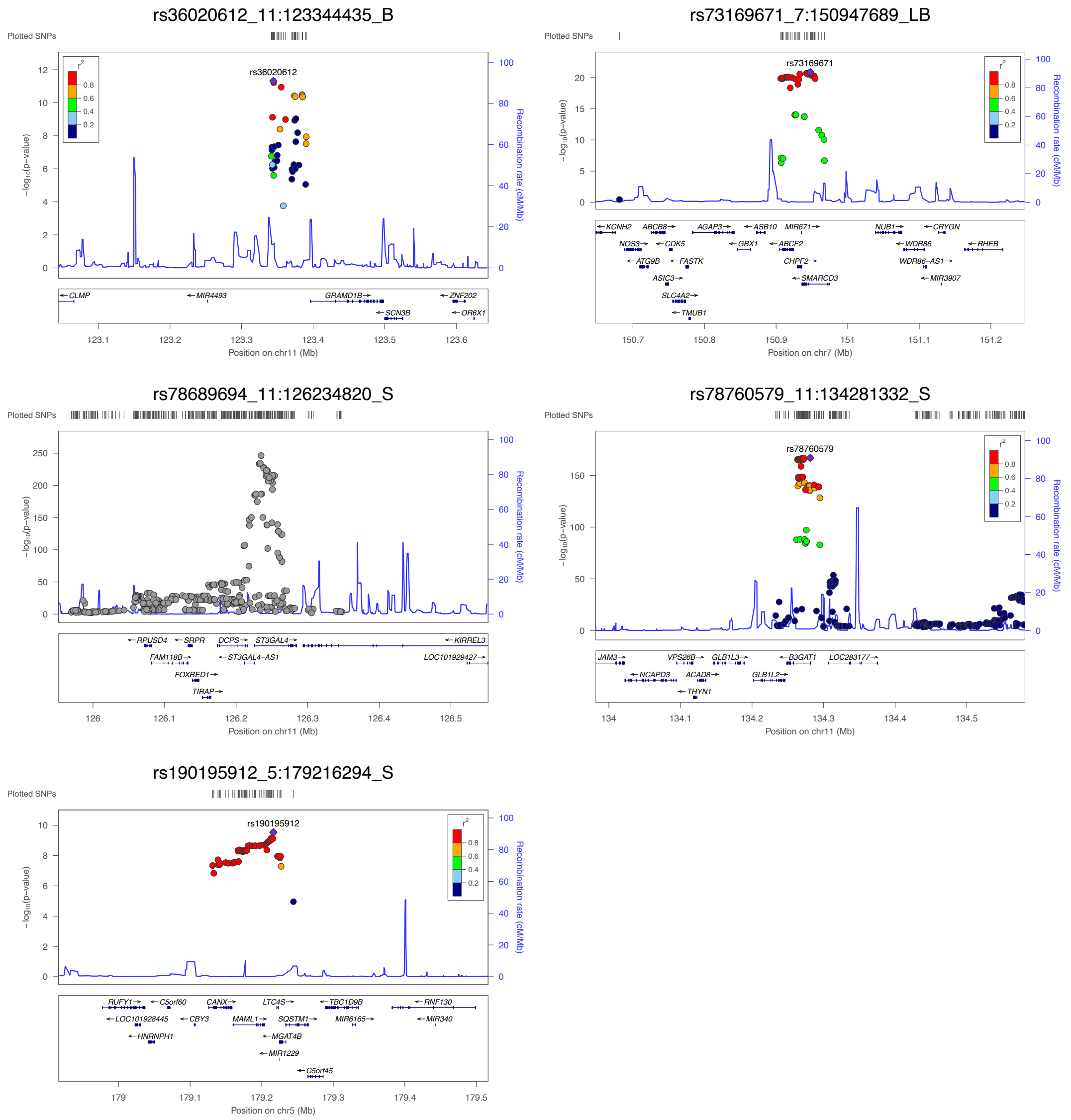

Supplementary Figure 2. Regional association plots for particular loci based on information about recombination rates, linkage disequilibrium between the SNP of interest and neighboring ones, and single-point association tests p-values.

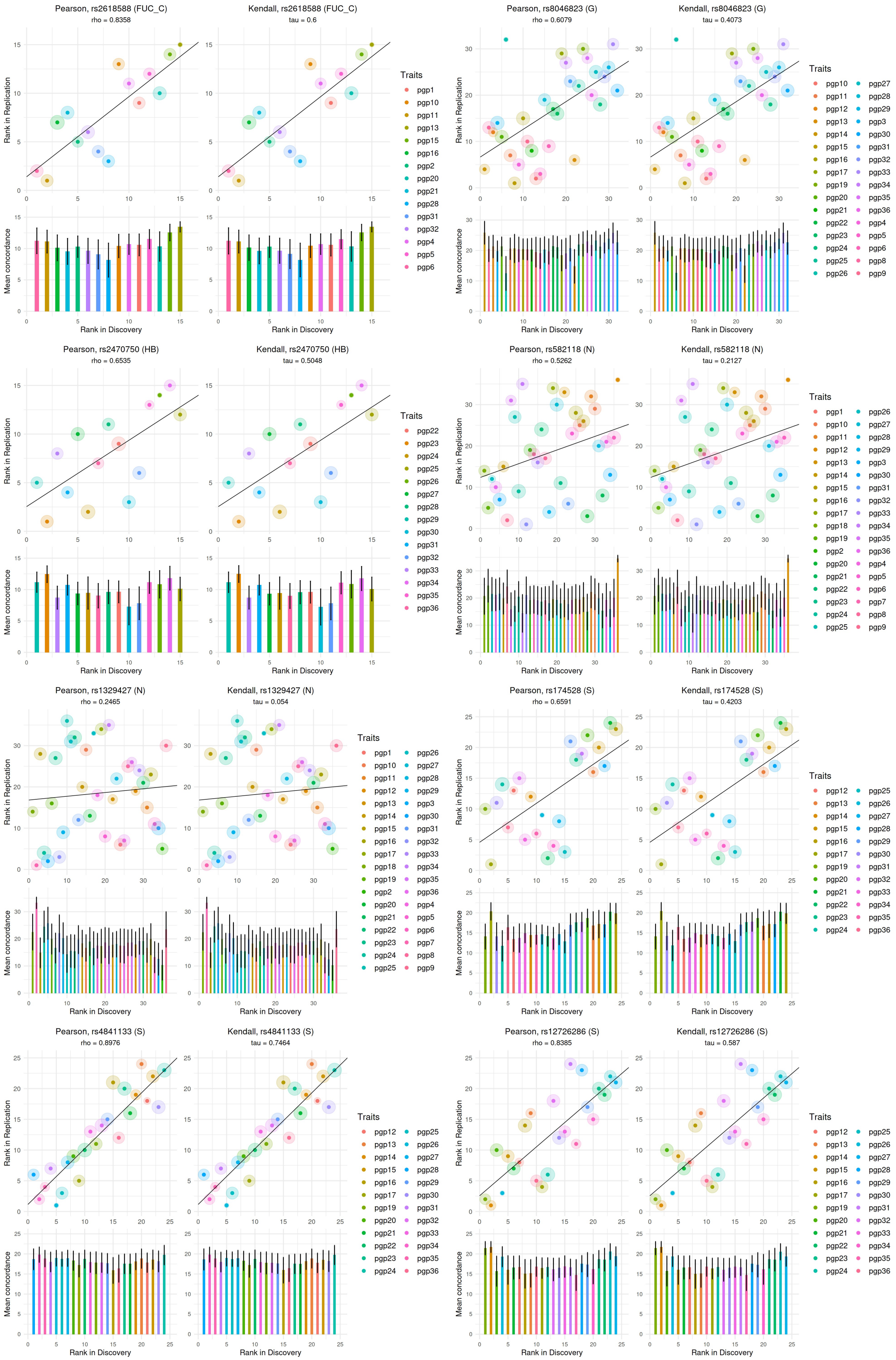
Pearson, rs2618588 (FUC_C)
rho = 0.8358
Kendall, rs2618588 (FUC_C)
tau = 0.6
Pearson, rs8046823 (G)
rho = 0.6079
Kendall, rs8046823 (G)
tau = 0.4073
Pearson, rs2470750 (HB)
rho = 0.6535
Kendall, rs2470750 (HB)
tau = 0.5048
Pearson, rs582118 (N)
rho = 0.5262
Kendall, rs582118 (N)
tau = 0.2127
Pearson, rs1329427 (N)
rho = 0.2465
Kendall, rs1329427 (N)
tau = 0.054
Pearson, rs174528 (S)
rho = 0.6591
Kendall, rs174528 (S)
tau = 0.4203
Pearson, rs4841133 (S)
rho = 0.8976
Kendall, rs4841133 (S)
tau = 0.7464
Pearson, rs12726286 (S)
rho = 0.8385
Kendall, rs12726286 (S)
tau = 0.587
Rank in Replication
Mean concordance
Rank in Discovery
Traits

Supplementary Figure 3. Results of Pearson and Kendall Correlation Methods for Replication of Multivariate Genetic Effects.

Each color represents one trait. There are two parts in this plot. In both parts, the x axis is the ranks of estimated marginal effect sizes in ascending order from discovery cohort. For the upper part, the y axis is the ranks from replication cohort. Therefore each dot represents the rank in discovery cohort and replication cohort for one trait. The radius of shade around a dot is proportional to the standard error of the estimated marginal effect. The standard errors are computed with variances in discovery cohort and replication cohort using inverse variance weights. To facilitate visualization, a regression line is added. Its slope equals to the Spearman's correlation. In the lower part, the y axis is the mean number of concordant pairs generated by a trait. If a trait has a very low bar, it means the trait disturbs the consistency of the whole rank pattern. The whiskers represents ± 1 times the standard deviation about the mean.

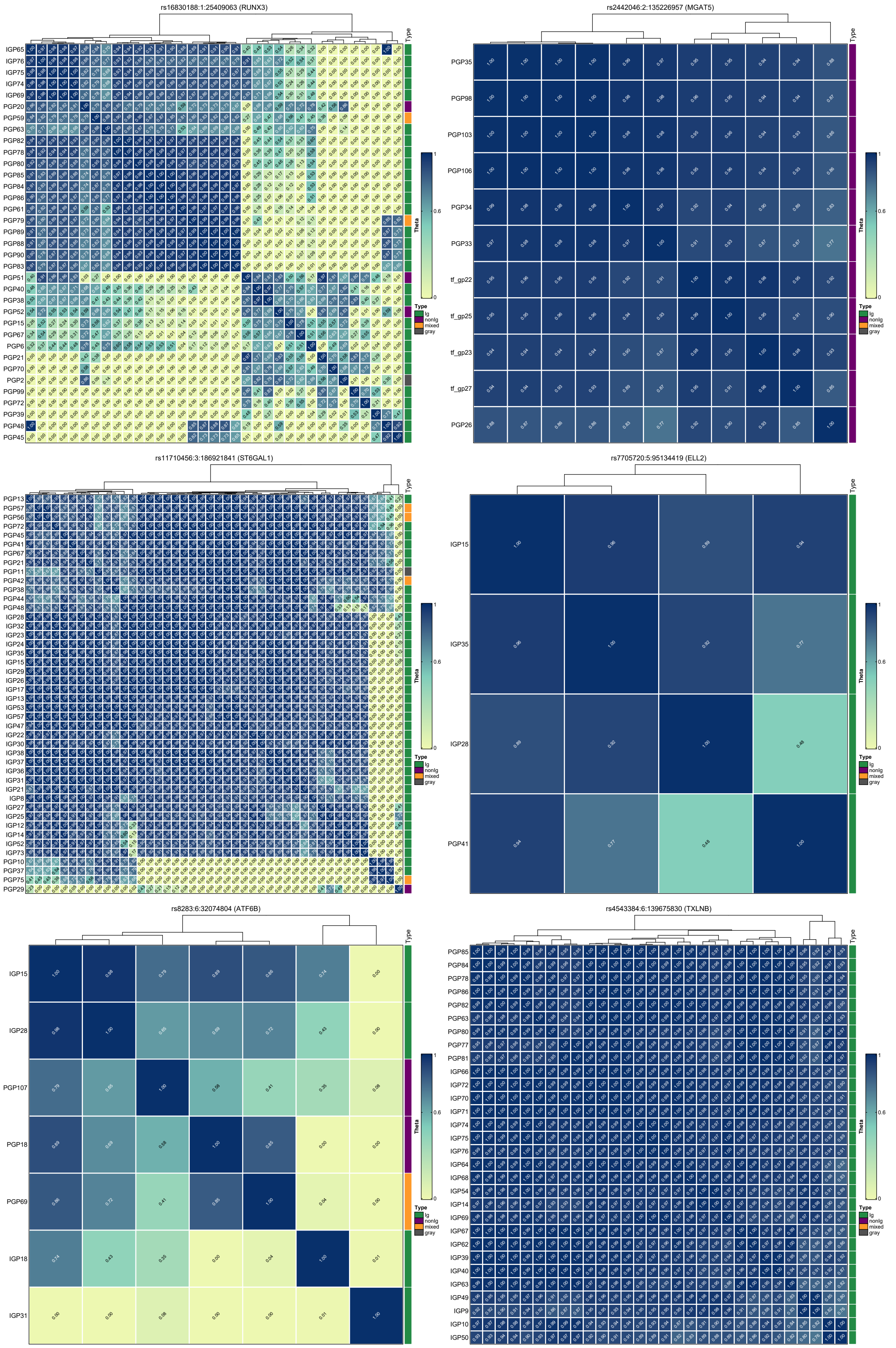
rs16830188:1:25409063 (RUNX3)
IGP65 IGP76 IGP75 IGP74 IGP69 PGP20 PGP59 PGP63 PGP82 PGP78 PGP80 PGP85 PGP84 PGP86 PGP61 PGP79 PGP89 PGP88 PGP90 PGP83 PGP51 PGP40 PGP38 PGP52 PGP15 PGP67 PGP6 PGP21 PGP70 PGP2 PGP99 PGP72 PGP39 PGP48 PGP45
rs2442046:2:135226957 (MGAT5)
PGP35 PGP98 PGP103 PGP106 PGP34 PGP33 tf_gp22 tf_gp25 tf_gp23 tf_gp27 PGP26
rs11710456:3:186921841 (ST6GAL1)
PGP13 PGP57 PGP56 PGP72 PGP45 PGP41 PGP67 PGP21 PGP11 PGP42 PGP38 PGP44 PGP48 IGP28 IGP32 IGP23 IGP24 IGP35 IGP15 IGP29 IGP26 IGP17 IGP13 IGP53 IGP57 IGP47 IGP22 IGP30 IGP38 IGP37 IGP36 IGP31 IGP21 IGP8 IGP27 IGP25 IGP12 IGP14 IGP52 IGP73 PGP10 PGP37 PGP75 PGP29
rs7705720:5:95134419 (ELL2)
IGP15 IGP35 IGP28 PGP41
rs8283:6:32074804 (ATF6B)
IGP15 IGP28 PGP107 PGP18 PGP69 IGP18 IGP31
rs4543384:6:139675830 (TXLNB)
PGP85 PGP84 PGP78 PGP86 PGP82 PGP63 PGP80 PGP77 PGP81 IGP66 IGP72 IGP70 IGP71 IGP74 IGP75 IGP76 IGP64 IGP68 IGP54 IGP14 IGP69 IGP67 IGP62 IGP39 IGP40 IGP63 IGP49 IGP9 IGP10 IGP50
Type
Theta
1
0.6
0
lg
nonlg
mixed
gray

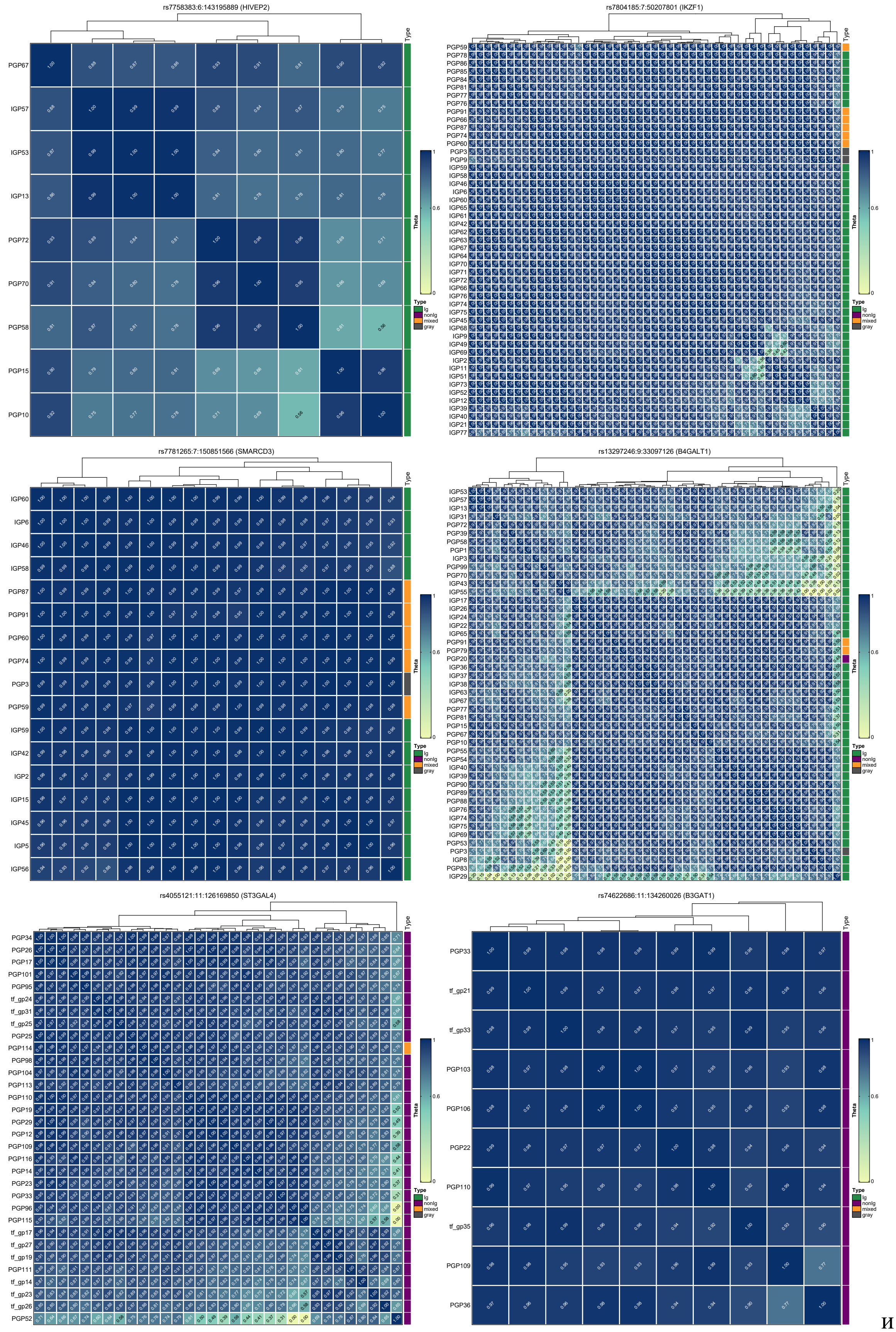
rs7758383:6:143195889 (HIVEP2)
rs7804185:7:50207801 (IKZF1)
rs7781265:7:150851566 (SMARCD3)
rs13297246:9:33097126 (B4GALT1)
rs4055121:11:126169850 (ST3GAL4)
rs74622686:11:134260026 (B3GAT1)
Type
Theta
lg
nonlg
mixed
gray

и

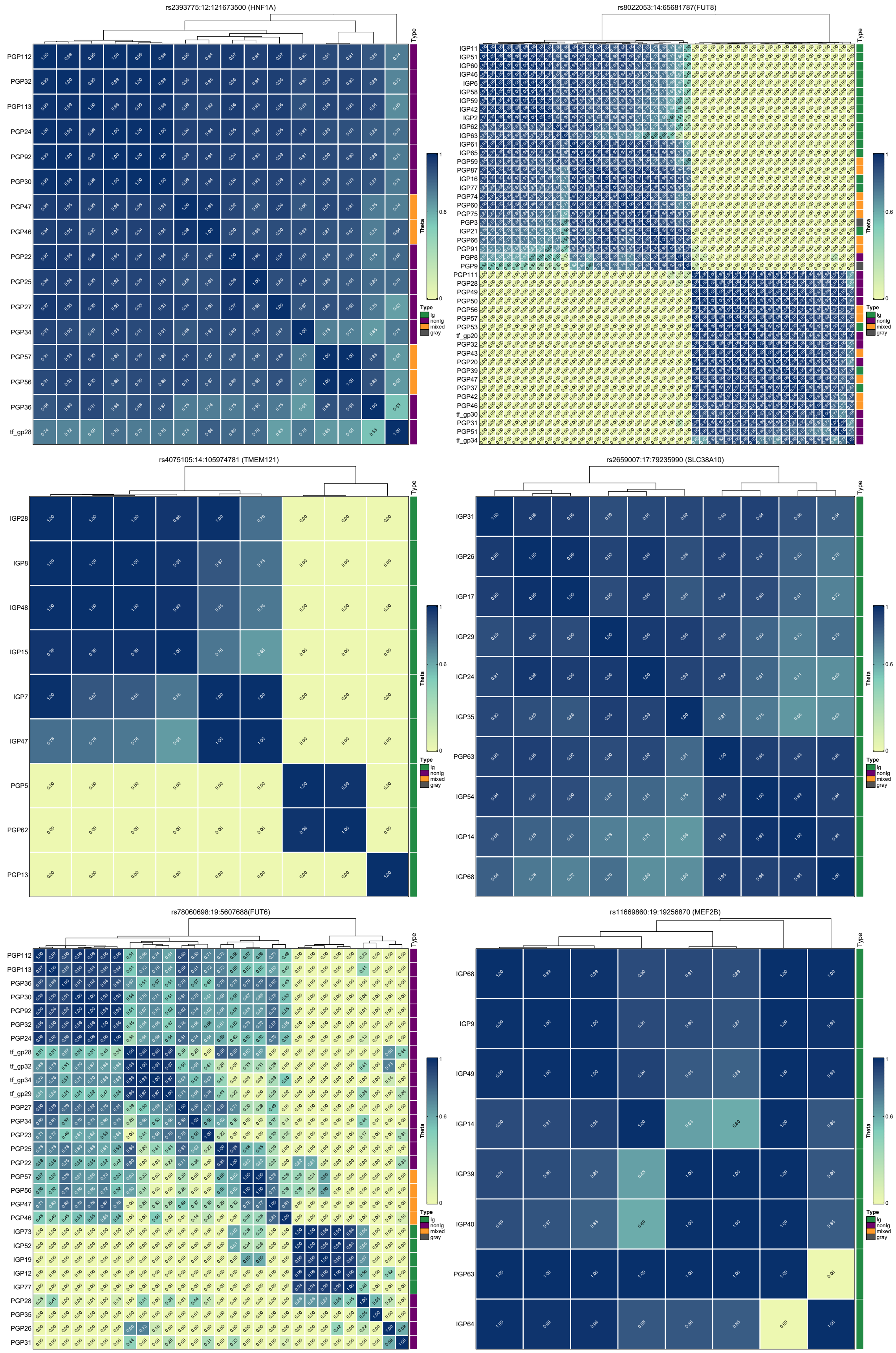
rs2393775:12:121673500 (HNF1A)
PGP112
PGP32
PGP113
PGP24
PGP92
PGP30
PGP47
PGP46
PGP22
PGP25
PGP27
PGP34
PGP57
PGP56
PGP36
tf_gp28
rs8022053:14:65681787(FUT8)
rs4075105:14:105974781 (TMEM121)
IGP28
IGP8
IGP48
IGP15
IGP7
IGP47
PGP5
PGP62
PGP13
rs2659007:17:79235990 (SLC38A10)
IGP31
IGP26
IGP17
IGP29
IGP24
IGP35
PGP63
IGP54
IGP14
IGP68
rs78060698:19:5607688(FUT6)
rs11669860:19:19256870 (MEF2B)
IGP68
IGP9
IGP49
IGP14
IGP39
IGP40
PGP63
IGP64
Type
Theta
lg
nonlg
mixed
gray

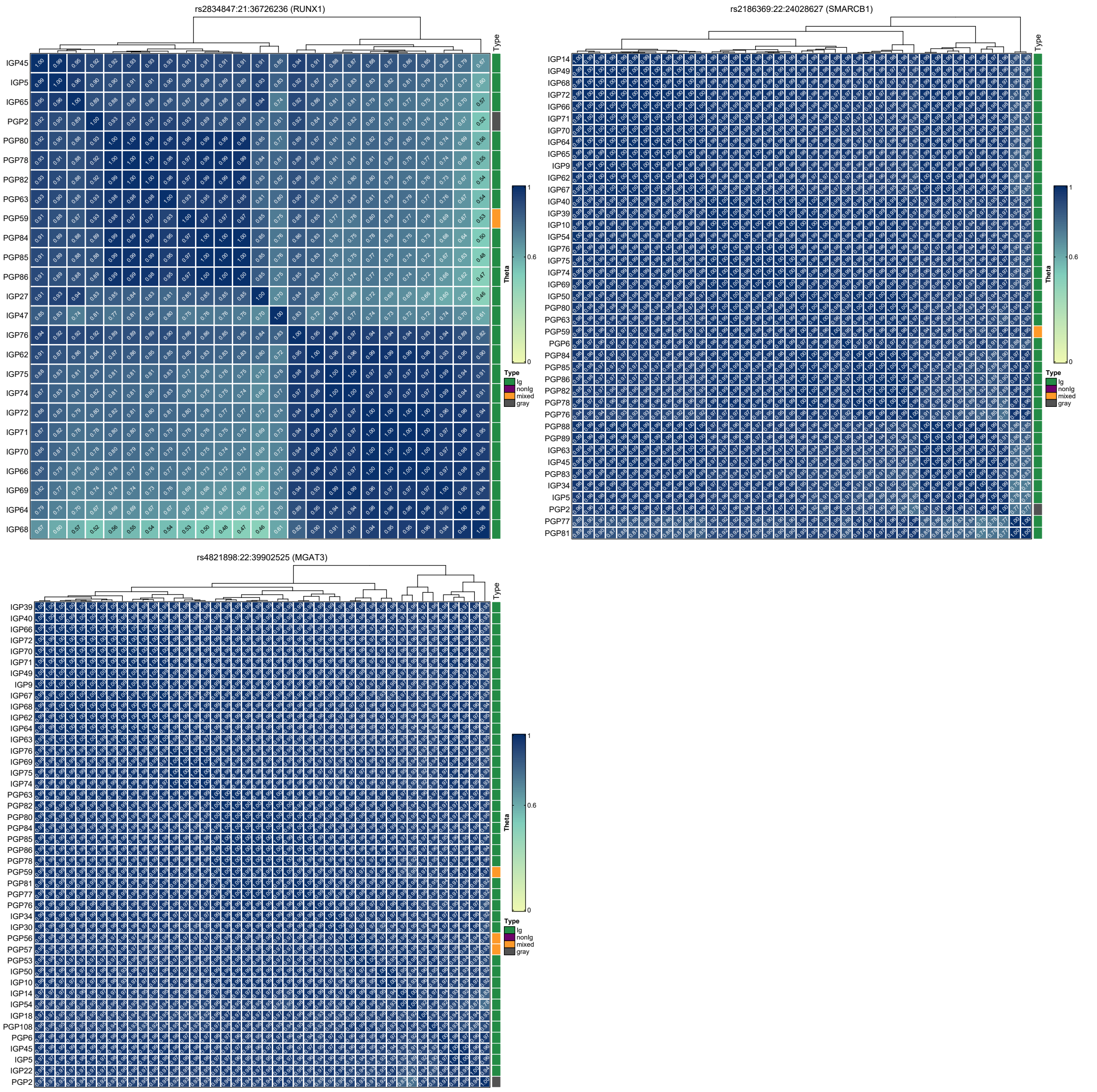

Supplementary Figure 4. Pairwise SMR-Θ colocalisation between TPNG traits and IgG or/and TF traits presented in the form of a heatmap for each particular locus. Each cell indicates the theta value between two glycan traits (PGP - total blood plasma glycan trait; IGP - IgG glycan trait; tf_gp - transferrin glycan trait). Only traits demonstrated significant association in the locus are shown. |Θ| > 0.6 indicates significant colocalization.

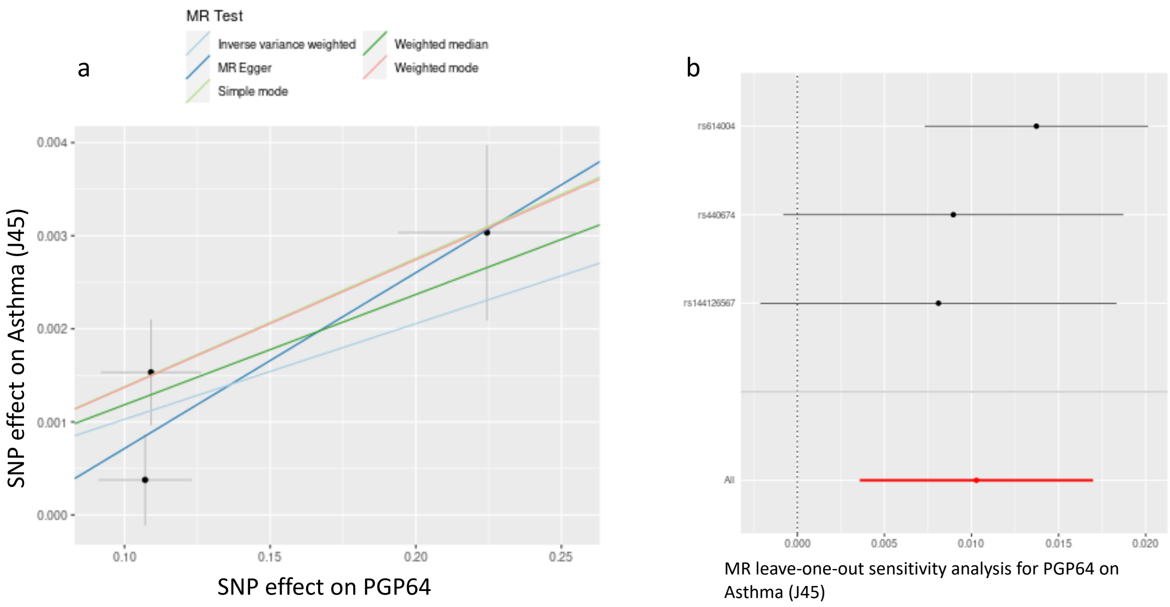

a) Relationship between the effects of each IV on PGP64 (X-axis) and on asthma (J45) (Y-axis), bars represent SE of the corresponding effects. The slopes of lines represent causal effects estimated by 5 different methods: IVW (light blue), MR-Egger (blue), simple mode (light green), weighted median (green), weighted mode (red).
b) Leave-one-out sensitivity analysis. Each black dot represents a causal effect of PGP64 on Asthma (J45) estimated with IVW method. Red dot represents the IWV estimate of the causal effect using all IVs. Lines represent 95%CI.

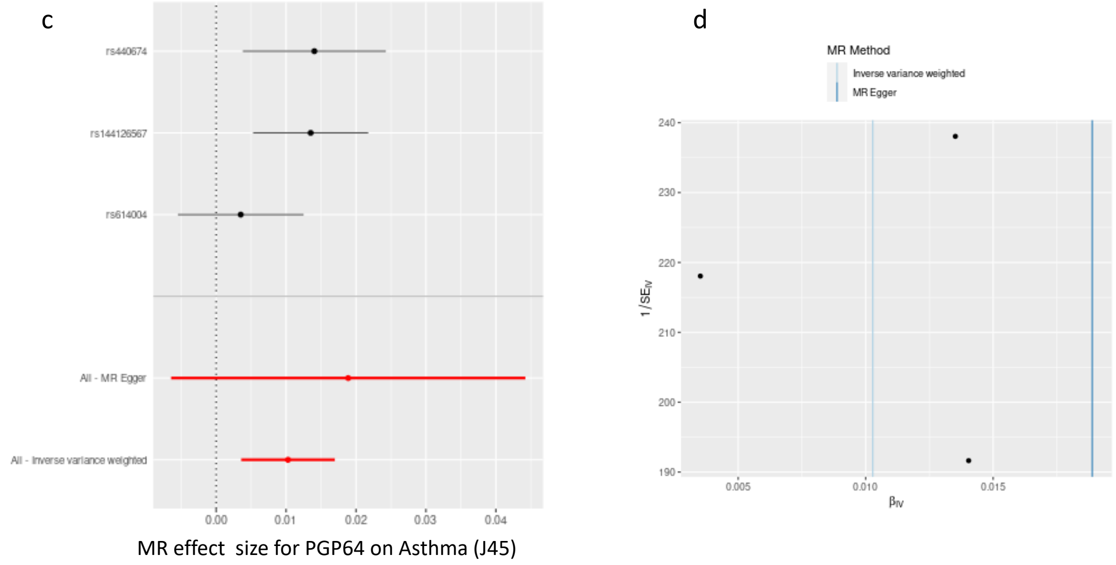

c) Forest plot, where each black dot represents a causal effect estimated for each individual instrument as log odds ratio of asthma (J45) per SD increased in PGP64. Red dots represent combined causal estimate using all SNPs together as estimated by MR-Egger and inverse-variance weighted (IVW) methods. Horizontal lines represent the 95% confidence intervals (CI).
d) Funnel plot, showing the relationship between the causal effect of PGP64 on asthma (J45) estimated for each individual IV against the inverse of the standard error of the causal estimate. Vertical lines show the causal estimates using all IVs for IVW. (light blue) and MR-Egger (blue) methods

Supplementary Figure 5. MR analysis of causal effect of PGP64 - The percentage of M6 in total neutral plasma glycans (GPn)- on asthma (J45)

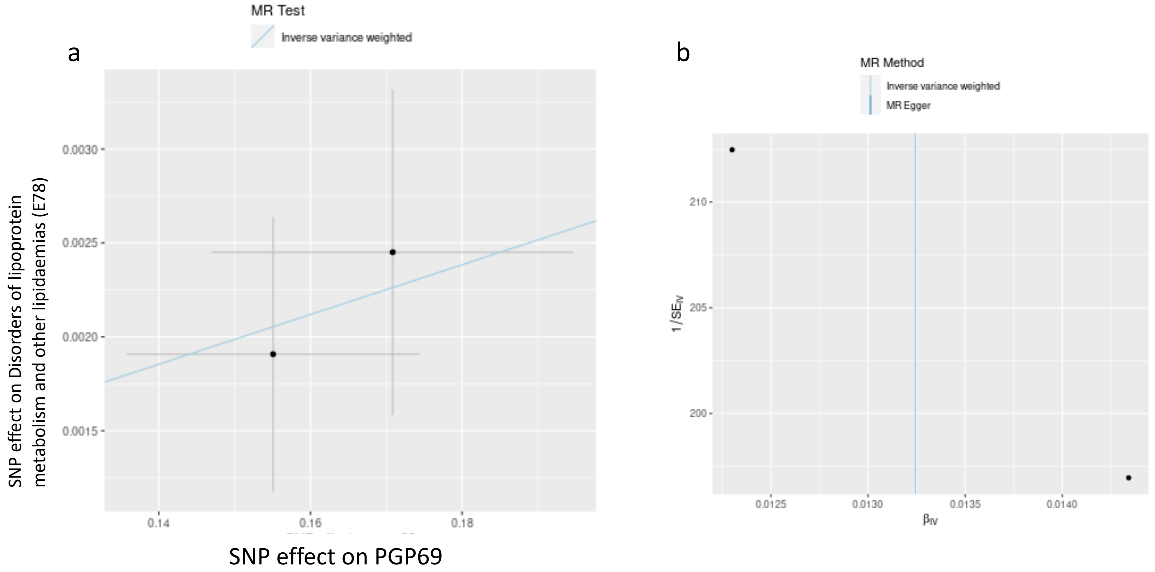

a) Relationship between the effects of each IV on PGP69 (X-axis) and on Disorders of lipoprotein metabolism and other lipidaemias (E78) (Y-axis), bars represent SE of the corresponding effects. The slopes of lines represent causal effect estimated by inverse variance weighted (IVW) method.
b) Funnel plot, showing the relationship between the causal effect of PGP69 on Disorders of lipoprotein metabolism and other lipidaemias (E78) estimated for each individual IV against the inverse of the standard error of the causal estimate. Vertical lines show the causal estimates using all IVs for IVW method.

S

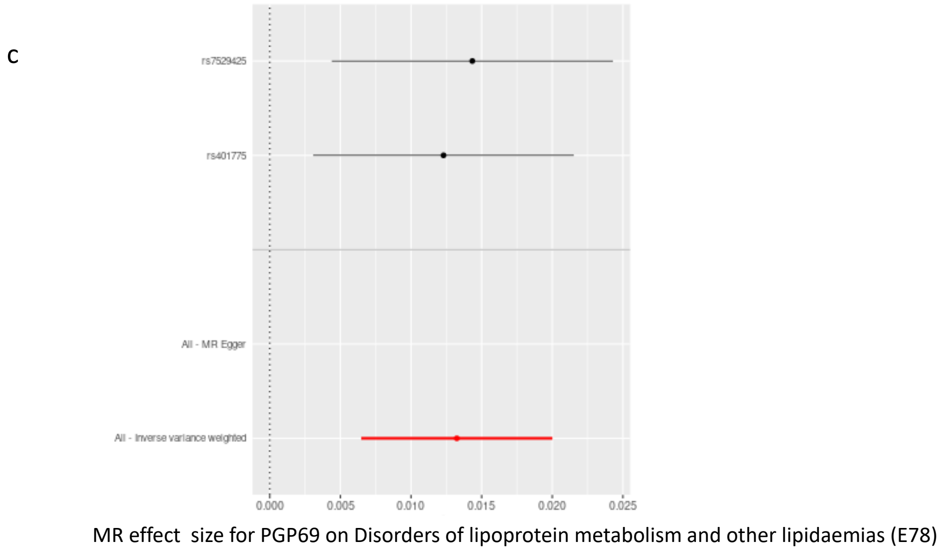

c) Forest plot, where each black dot represents a causal effect estimated for each individual instrument as log odds ratio of asthma (J45) per SD increased in PGP64. Red dots represent combined causal estimate using all SNPs together as estimated by IVW method. Horizontal lines represent the 95% confidence intervals (CI).

Supplementary Figure 6. MR analysis of causal effect of PGP69 -The percentage of M9 in total neutral plasma glycans (GPn) - on Disorders of lipoprotein metabolism and other lipidaemias (E78)

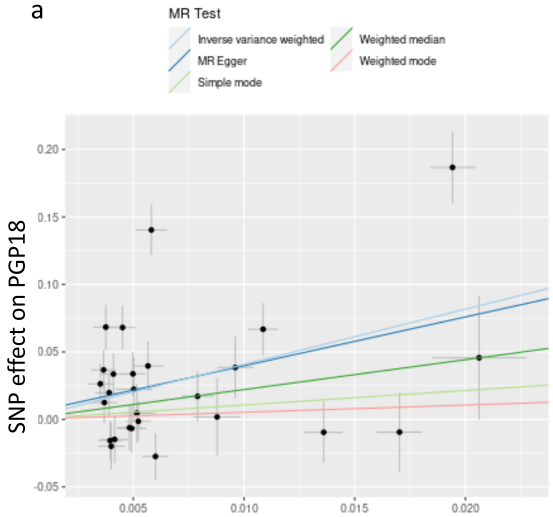

SNP effect on Disorders of lipoprotein metabolism and other lipidaemias (E78) on PGP18

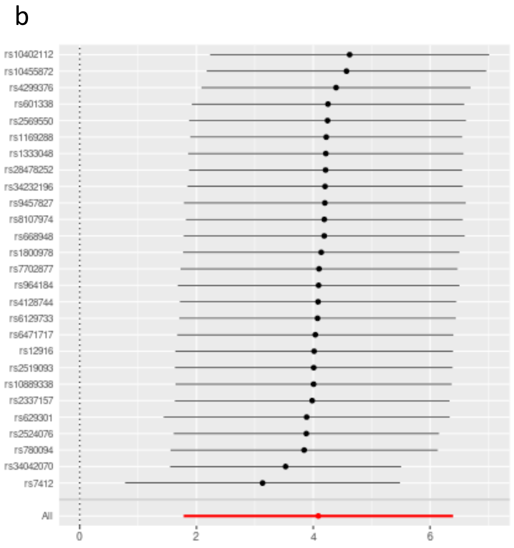

MR leave-one-out sensitivity analysis for Disorders of lipoprotein metabolism and other lipidaemias (E78) on PGP18

a) Relationship between the effects of each IV on E78 (X-axis) and on PGP18 (Y-axis), bars represent SE of the corresponding effects. The slopes of lines represent causal effects estimated by 5 different methods: IVW (light blue), MR-Egger (blue), simple mode (light green), weighted median (green), weighted mode (red).

b) Leave-one-out sensitivity analysis. Each black dot represents a causal effect of Disorders of lipoprotein metabolism and other lipidaemias (E78) on PGP18 estimated with IVW method. Red dot represents the IWV estimate of the causal effect using all IVs. Lines represent 95%CI.

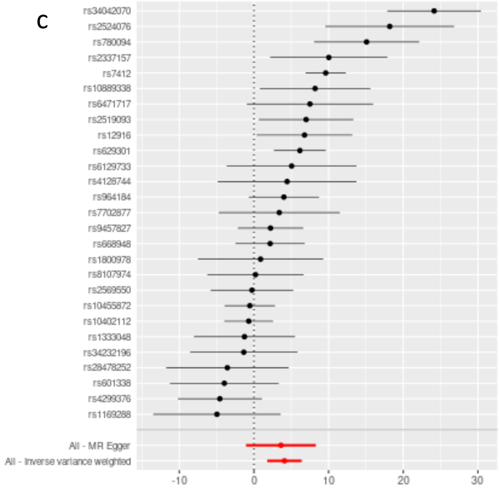

MR effect size for Disorders of lipoprotein metabolism and other lipidaemias (E78) on PGP18

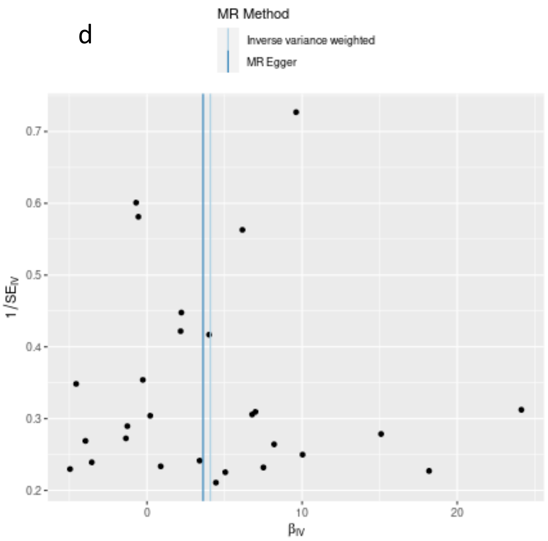

c) Forest plot, where each black dot represents a causal effect estimated for each individual instrument as SD of PGP18 per increase in 1 unit of log odds ratio of Disorders of lipoprotein metabolism and other lipidaemias (E78). Red dots represent combined causal estimate using all SNPs together as estimated by MR-Egger and inverse-variance weighted (IVW) methods. Horizontal lines represent the 95% confidence intervals (CI).

d) Funnel plot, showing the relationship between the causal effect of Disorders of lipoprotein metabolism and other lipidaemias (E78) on PGP18 estimated for each individual IV against the inverse of the standard error of the causal estimate. Vertical lines show the causal estimates using all IVs for IVW. (light blue) and MR-Egger (blue) methods

Supplementary Figure 7. MR analysis of causal effect of Disorders of lipoprotein metabolism and other lipidaemias (E78) on PGP18 - the percentage of M9

a

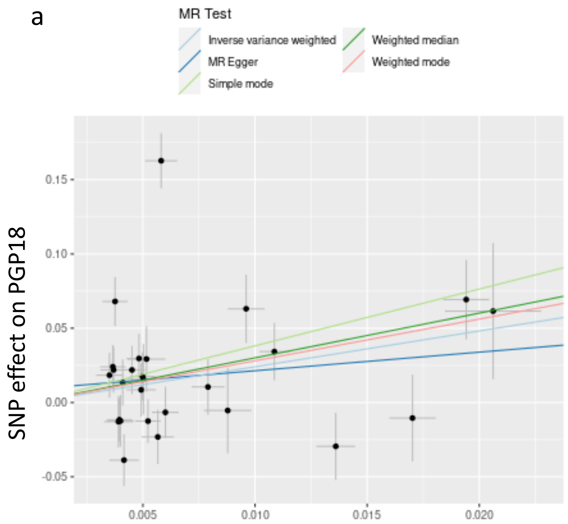

SNP effect on Disorders of lipoprotein metabolism and other lipidaemias (E78) on PGP107

b

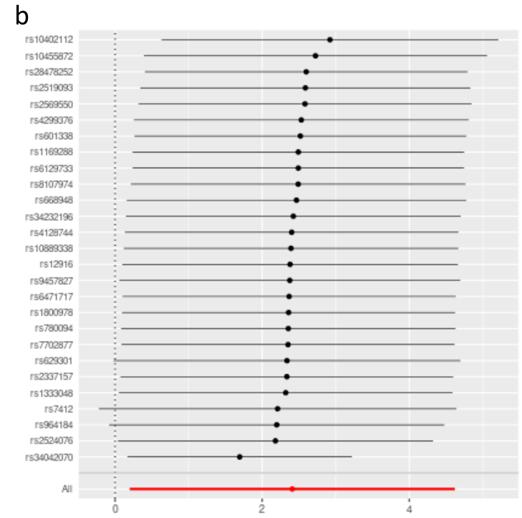

MR leave-one-out sensitivity analysis for Disorders of lipoprotein metabolism and other lipidaemias (E78) on PGP107

a) Relationship between the effects of each IV on E78 (X-axis) and on PGP107 (Y-axis), bars represent SE of the corresponding effects. The slopes of lines represent causal effects estimated by 5 different methods: IVW (light blue), MR-Egger (blue), simple mode (light green), weighted median (green), weighted mode (red).
b) Leave-one-out sensitivity analysis. Each black dot represents a causal effect of Disorders of lipoprotein metabolism and other lipidaemias (E78) on PGP107 estimated with IVW method. Red dot represents the IWV estimate of the causal effect using all IVs. Lines represent 95%CI.

c

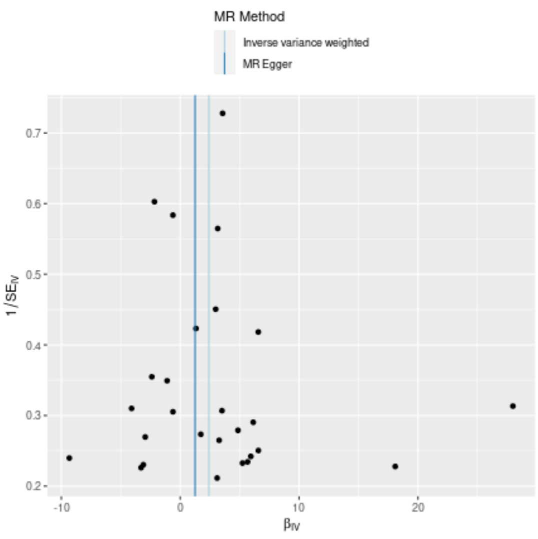

d

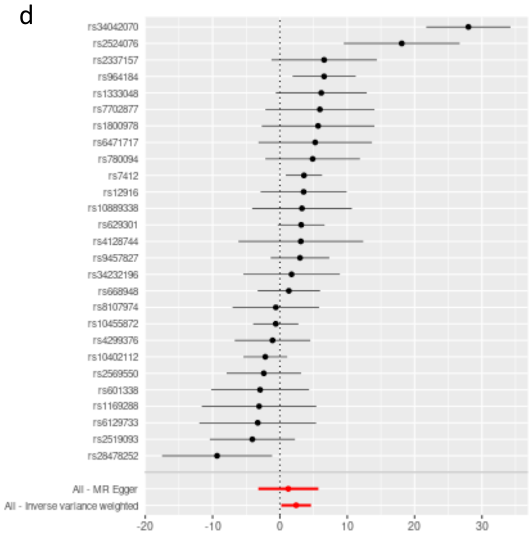

MR effect size for Disorders of lipoprotein metabolism and other lipidaemias (E78) on PGP107

c) Funnel plot, showing the relationship between the causal effect of Disorders of lipoprotein metabolism and other lipidaemias (E78) on PGP107 estimated for each individual IV against the inverse of the standard error of the causal estimate. Vertical lines show the causal estimates using all IVs for IVW. (light blue) and MR-Egger (blue) methods.
d) Forest plot, where each black dot represents a causal effect estimated for each individual instrument as SD of PGP107 per increase in 1 unit of log odds ratio of Disorders of lipoprotein metabolism and other lipidaemias (E78). Red dots represent combined causal estimate using all SNPs together as estimated by MR-Egger and inverse-variance weighted (IVW) methods. Horizontal lines represent the 95% confidence intervals (CI).

Supplementary Figure 8. MR analysis of causal effect of Disorders of lipoprotein metabolism and other lipidaemias (E78) on PGP107 - the percentage of high-mannose structures in total plasma glycans

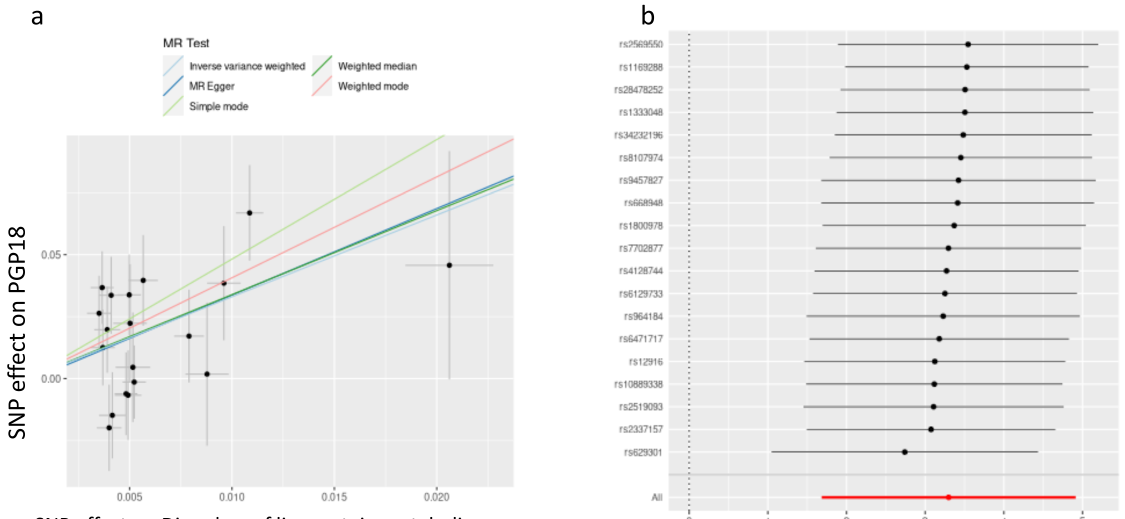

SNP effect on Disorders of lipoprotein metabolism and other lipidaemias (E78) on PGP18

MR leave-one-out sensitivity analysis for Disorders of lipoprotein metabolism and other lipidaemias (E78) on PGP18

a) Relationship between the effects of each IV on E78 (X-axis) and on PGP18 (Y-axis), bars represent SE of the corresponding effects. The slopes of lines represent causal effects estimated by 5 different methods: IVW (light blue), MR-Egger (blue), simple mode (light green), weighted median (green), weighted mode (red).
b) Leave-one-out sensitivity analysis. Each black dot represents a causal effect of Disorders of lipoprotein metabolism and other lipidaemias (E78) on PGP18 estimated with IVW method. Red dot represents the IWV estimate of the causal effect using all IVs. Lines represent 95%CI.

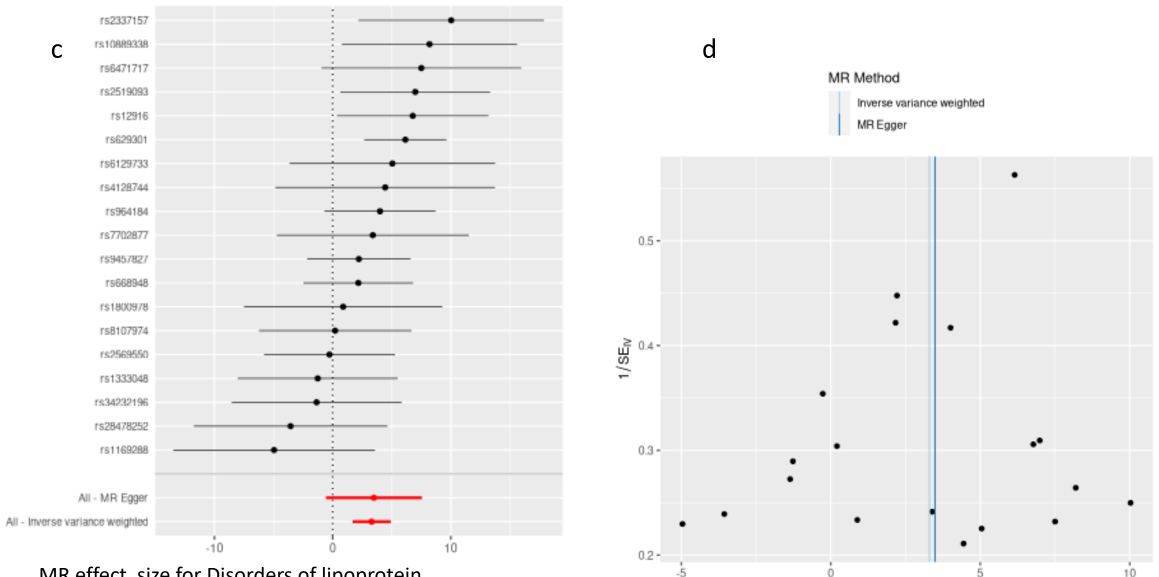

MR effect size for Disorders of lipoprotein metabolism and other lipidaemias (E78) on PGP18

c) Forest plot, where each black dot represents a causal effect estimated for each individual instrument as SD of PGP18 per increase in 1 unit of log odds ratio of Disorders of lipoprotein metabolism and other lipidaemias (E78). Red dots represent combined causal estimate using all SNPs together as estimated by MR-Egger and inverse-variance weighted (IVW) methods. Horizontal lines represent the 95% confidence intervals (CI).
d) Funnel plot, showing the relationship between the causal effect of Disorders of lipoprotein metabolism and other lipidaemias (E78) on PGP18 estimated for each individual IV against the inverse of the standard error of the causal estimate. Vertical lines show the causal estimates using all IVs for IVW. (light blue) and MR-Egger (blue) methods

Supplementary Figure 9. MR analysis of causal effect of Disorders of lipoprotein metabolism and other lipidaemias (E78) on PGP18 - the percentage of M9 - without pleiotropic SNPs.

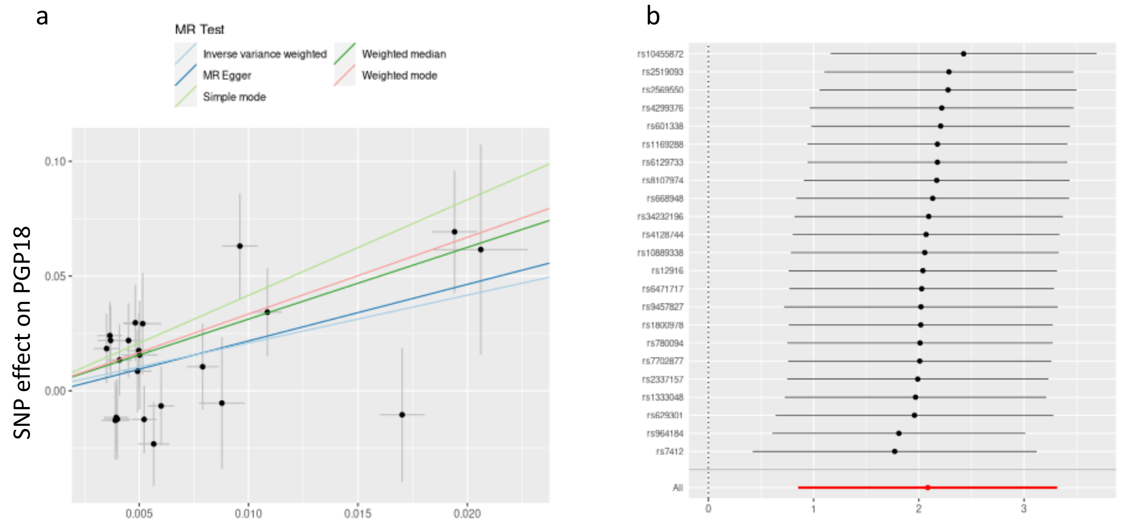

SNP effect on Disorders of lipoprotein metabolism and other lipidaemias (E78) on PGP107

MR leave-one-out sensitivity analysis for Disorders of lipoprotein metabolism and other lipidaemias (E78) on PGP107

a) Relationship between the effects of each IV on E78 (X-axis) and on PGP107 (Y-axis), bars represent SE of the corresponding effects. The slopes of lines represent causal effects estimated by 5 different methods: IVW (light blue), MR-Egger (blue), simple mode (light green), weighted median (green), weighted mode (red).
b) Leave-one-out sensitivity analysis. Each black dot represents a causal effect of Disorders of lipoprotein metabolism and other lipidaemias (E78) on PGP107 estimated with IVW method. Red dot represents the IWV estimate of the causal effect using all IVs. Lines represent 95%CI.

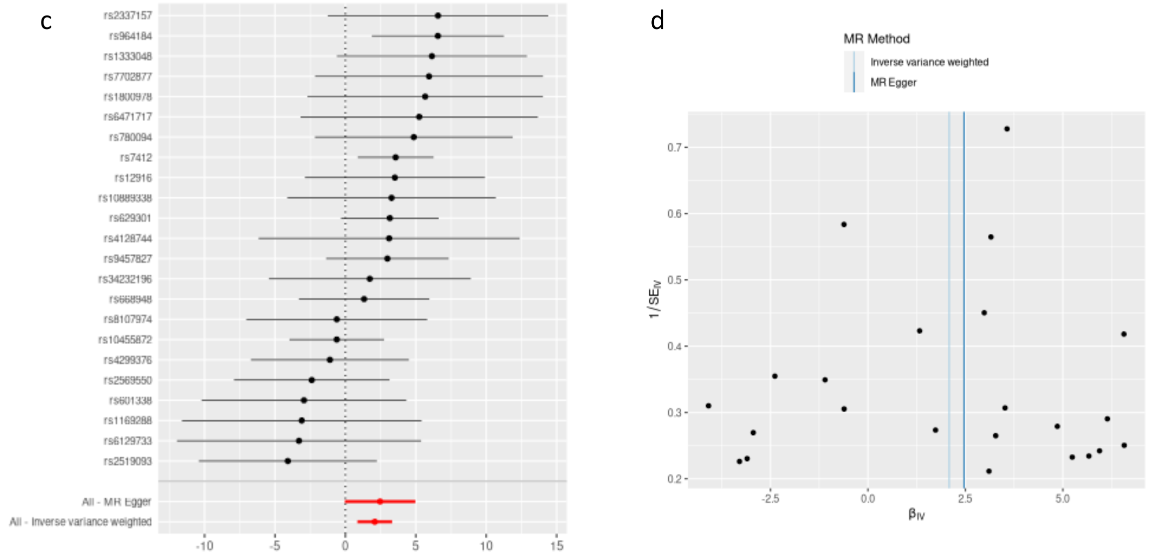

MR effect size for Disorders of lipoprotein metabolism and other lipidaemias (E78) on PGP107

c) Forest plot, where each black dot represents a causal effect estimated for each individual instrument as SD of PGP107 per increase in 1 unit of log odds ratio of Disorders of lipoprotein metabolism and other lipidaemias (E78). Red dots represent combined causal estimate using all SNPs together as estimated by MR-Egger and inverse-variance weighted (IVW) methods. Horizontal lines represent the 95% confidence intervals (CI).

d)Funnel plot, showing the relationship between the causal effect of Disorders of lipoprotein metabolism and other lipidaemias (E78) on PGP107 estimated for each individual IV against the inverse of the standard error of the causal estimate. Vertical lines show the causal estimates using all IVs for IVW. (light blue) and MR-Egger (blue) methods.

Supplementary Figure 10. MR analysis of causal effect of Disorders of lipoprotein metabolism and other lipidaemias (E78) on PGP107 - the percentage of high-mannose structures in total plasma glycans - without pleiotropic SNPs

Supplementary Notes

Table of contents

Study cohort description 28
TwinsUK 28
EPIC-Potsdam 28
PainOmics 28
SOCCS 29
SABRE 29
QMDiab 29
CEDAR 29
Genotyping 30
TwinsUK 30
EPIC-Potsdam 30
PainOmics 31
SOCCS 31
SABRE 31
QMDiab 31
CEDAR 31
Phenotyping 32
Plasma N-glycome quantification 32
Harmonization of glycan peaks 32
Normalization and batch-correction of GPs 32
Univariate genome-wide association analysis 33
Discovery and replication genome-wide association meta-analysis 34
Locus definition 34
Multivariate genome-wide association analysis 34
Phenotype grouping for multivariate analysis 34
Discovery multivariate analysis 35
Replication 35
Statistical power calculation 36

Trait selection for the colocalization-analysis 37
Classification of glycans into Ig-related and non-Ig 38
Systematic prioritization of effector genes for protein glycosylation........ 39
Glycosyltransferases 39
N-glycoproteins 40
Transcription factors 40
Membrane transporters 41
Lipid and glucose metabolism 42
Association between PGS for plasma N-glycosylation traits and ICD-10 diseases 43
Disease phenotypes, significantly associated with N-glycosylation of plasma proteins . 43
Biochemically related groups of plasma N-glycosylation traits, associated with ICD-10 disease phenotypes 45
Causal effect of plasma N-glycosylation traits on ICD-10 disease phenotypes 46
Causal effect of ICD-10 disease phenotypes on plasma N-glycosylation traits 47
Effect of disorders of lipoprotein metabolism and other lipidaemias (E78) on PGP18 .. 47
Effect of disorders of lipoprotein metabolism and other lipidaemias (E78) on PGP107 47
Sensitivity analyses 47
Effect of disorders of lipoprotein metabolism and other lipidaemias (E78) on PGP18 48
Effect of disorders of lipoprotein metabolism and other lipidaemias (E78) on PGP107 48
Tissue-specificity of eQTLs........ 48
References........ 49

Study cohort description

TwinsUK

The TwinsUK cohort[1] (also referred to as the UK Adult Twin Register) is an a nationwide registry of volunteer twins in the United Kingdom, with about 13,000 registered twins (83% female, equal number of monozygotic and dizygotic twins, predominantly middle-aged and older). The Department of Twin Research and Genetic Epidemiology at King's College London (KCL) hosts the registry. From this registry, 2,763 subjects had total plasma N-linked glycan measurements, which were included in the analysis.

EPIC-Potsdam

The prospective EPIC-Potsdam cohort study includes 27,548 participants (16,644 women and 10,904 men), who were recruited within an age-range of 35-65 years from the general population between 1994 and 1998[2]. Baseline assessments comprised anthropometric measures and blood sampling by qualified medical personnel. Blood pressure was measured with the individual sitting with the arm elevated at heart level by using oscillometric devices (BOSO-Oscillomat; Bosch & Sohn, Jungingen, Germany), and the mean of the second and third reading was used. Lifestyle, sociodemographic characteristics, and current health status were assessed with validated questionnaires and in face-to-face interviews[3]. Participants were then actively contacted by sending out questionnaires and, if necessary, by telephone every 2–3 years with response rates between 90 and 96% per follow-up round[4]. From all participants who provided blood at baseline (N = 26,437), a random sample (subcohort, N = 2,500) was drawn, which served as study population for the present analysis. All participants gave written informed consent for biomedical research, and the study was approved by the Ethics Committee of the State of Brandenburg, Germany[2].

PainOmics

The PainOmics[5] study of patients comprised a retrospective multicenter study (ClinicalTrials.gov Identifier NCT02037789) part of the PainOmics project funded by European Community in the Seventh Framework Programme (Project ID: 602736). The primary objective is to recognize genetic variants associated with chronic low back pain (CLBP); secondary objectives are to study glycomic and proteomic profiles associated with CLBP. Glycomic and Activomic approaches aim to reveal alterations in proteome complexity that arise from the post-translational modification that varies in response to changes in the physiological environment, a particularly important avenue to explore in chronic inflammatory diseases. The study was firstly approved by the Institutional Review Boards of IRCCS Foundation San Matteo Hospital Pavia and then by the Institutional Review boards of all clinical centers (King's College London, ZOL Genk/Lanaken, St. Catherine Specialty Hospital) that enrolled patients. Copies of approvals were provided to the European Commission before starting the study. Written informed consent was obtained from all participants. In the period between September 2014 and February 2016, 3,400 patients were enrolled. Sample collection was performed in all patients enrolled, according to the Standard Operating Procedures (SOP) published in PlosOne in 2017[6]. Samples were processed in the PainOmics laboratory in a blinded manner in the University of Parma. Blood collections for

omics analyses were taken at the time of enrollment. All blood samples were processed and stored according to SOP protocols until they were sent to analytical laboratories for omics analysis.

SOCCS

SOCCS study[7,8] comprised 2,057 colorectal cancer cases (CRC) (61% male; mean age at diagnosis (65.8 ± 8.4) years) and 2,111 population controls (60% males; mean age 67.9±9.0 years) as ascertained in Scotland. Cases were taken from an independent prospective incident CRC case series and were aged < 80 years at diagnosis. Control subjects were population controls matched by age (±5 years), gender, and area of residence within Scotland. All participants gave written informed consent and study approval was from the MultiCentre Research Ethics Committee for Scotland and Local Research Ethics committee. Sample collection is described in[7,8].

SABRE

SABRE is a population-based cohort initiated in 4,857 people of European, South Asian and African Caribbean origin aged 40 to 69 living in West London, UK[9]. Peripheral blood samples were collected from the Southall participants at baseline (1988–91) for DNA extraction. We used data from the European-origin participants. Plasma N-glycome quantification was performed using samples collected at the 20-year follow-up visit (2008-2011). All participants gave written informed consent. Approval for the baseline study was obtained from Ealing, Hounslow, and Spelthorne, Parkside and University College London research ethnics committees.

QMDiab

The Qatar Metabolomics Study on Diabetes (QMDiab) is a cross-sectional case–control study with 374 participants. QMDiab has been described previously and comprises male and female participants in near equal proportions, aged between 23 and 71 years, mainly of Arab, South Asian and Filipino descent[10,11]. The initial study was approved by the Institutional Review Boards of HMC and Weill Cornell Medicine—Qatar (WCM-Q) (research protocol #11131/11). Written informed consent was obtained from all participants. All study participants were enrolled between February 2012 and June 2012 at the Dermatology Department of Hamad Medical Corporation (HMC) in Doha, Qatar. Inclusion criteria were a primary form of type 2 diabetes (for cases) or an absence of type 2 diabetes (for controls). Sample collection was conducted in the afternoon, after the general operating hours of the morning clinic. Patient and control samples were collected in a random order as they became available and at the same location using identical protocols, instruments and study personnel. Samples from cases and controls were processed in the laboratory in parallel and in a blinded manner. Data from five participants were excluded from the analysis because of incomplete records, leaving 176 patients and 193 controls. Of the 193 control participants initially enrolled, 12 had HbA1c levels above 6.5% (48 mmol/mol) and were subsequently classified as cases, resulting in 188 cases and 181 controls.

CEDAR

The Correlated Expression and Disease Association Research (CEDAR) study was described previously[12]. Peripheral blood as well as intestinal biopsies (ileum, transverse colon, rectum) was collected from 323 healthy Europeans visiting the Academic Hospital of the University of Liège as part of a national screening campaign for colon cancer. Participants included

182 women and 141 men, averaging 56 years of age (range: 19-86). Enrolled individuals were not suffering any autoimmune or inflammatory disease and were not taking corticosteroids or non-steroid anti-inflammatory drugs (with the exception of low doses of aspirin to prevent thrombosis). Researchers recorded birth date, weight, height, smoking history, declared ethnicity and hematological parameters (red blood cell count, platelet count, differential white blood cell count) for each individual. The experimental protocol was approved by the ethics committee of the University of Liège Academic Hospital. Informed consent was obtained prior to donation in agreement with the recommendations of the declaration of Helsinki for experiments involving human subjects.

Genotyping

Genotyping was carried out using the SNP-array with genome-wide coverage, followed by genomic imputation. The genotype data quality control was conducted using the protocol, guidelines and software, described in[13].

TwinsUK

Genotyping was carried out using combination Illumina SNP arrays: HumanHap300, HumanHap610Q, 1M-Duo and 1.2MDuo 1M. Standard quality control of genotyped data was applied, with SNPs filtered by sample call rate > 98%, MAF > 1%, SNP call rate: >97% (for SNP with MAF≥5%) or >99% (for SNPs with 1% ≤ MAF < 5%), HWE $P \leq 1 \times 10^{-6}$. In total 275,139 SNPs passed the criteria. Imputation was done using IMPUTE2 software with 1000G phase 1 version 3 and mapped to the GRCh37 human genome build. Imputed SNPs were filtered by imputation quality (SNPTEST proper-info) > 0.7, MAF >= 1%; MAC >= 10; leading to 8,557,543 SNPs passed to the GWAS analysis.

EPIC-Potsdam

Genotyping was carried out using three different genotyping arrays: Human660W-Quad_v1_A (N = 325), HumanCoreExome-12v1-0_B (N = 622) and Illumina InfiniumOmniExpressExome-8v1-3_ADNA Analysis BeadChip (N = 1,245).Genotyping and quality control of the Human660W-Quad_v1_A and HumanCoreExome-12v1-0_B chips was described elsewhere[14]. Genotype calling and quality control of the samples genotyped with InfiniumOmniExpressExome-8v1-3_ADNA Analysis BeadChip were carried out jointly using Illumina's GenomeStudio v2011.1 software suite and protocols suggested by the CHARGE consortium[15], Anderson et al[13] and Guo et al.[16]. To improve the genotype calling for rare variants zCall with a threshold of 7 was applied[17]. Individuals with low call rate, discordant sex information (F value between 0.2 and 0.8), related or duplicated individuals (IBD > 0.185) and individuals with divergent ancestry were excluded from further analysis. Phasing and imputation were conducted using the Michigan Imputation Service[18]. The Haplotype Reference Consortium (release 1.1) was used as reference panel[19]. Before imputation pre-phasing was applied using Eagle2[20]. Imputation was carried out in four separated datasets (one for each genotyping chip or two for the HumanCoreExome-12v1-0_B chip) using minimac3[18]. Genotypes map to the GRCh37 human genome build.

PainOmics

Genotyping was carried out using Illumina HumanCore BeadChip and Illumina GSA. Quality control (QC) of genotype was conducted separately for each SNP-array. Standard QC of genotyped data was applied with SNPs filtered by a sample call rate > 98%, minor allele frequency (MAF) > 0.625%, SNP call rate: > 97%, Hardy-Weinberg equilibrium (HWE) $P \leq 1\times10^{-6}$. Imputation was done using the Eagle software tool with HRC r1.1 2016 reference and mapped to the GRCh37 human genome build.

SOCCS

Details of the genotyping procedure can be found in [21] and in [22]. Genotyping was carried out using Illumina SNP arrays HumanHap300, HumanHap240S or Infinium OmniExpressExome-8. Standard QC of genotype data was applied, with SNPs filtered by sample call rate > 95%, MAF > 1%, SNP call rate in autosomal chromosomes > 99%, HWE $P \leq$ Bonferonni cut-off > 0.05/number of tested SNPs. In total, 514,177 SNPs passed these criteria. Imputation was done using SHAPEIT and IMPUTE2 software with 1000 Genomes, phase 1 (Integrated haplotypes, released June 2014) and mapped to the GRCh37 human genome build.

SABRE

Genotyping was done using the Illumina Human Core Bead Chip. In total, 1429 samples of European descent were genotyped. QC of genotype data was conducted separately for each ethnic group. Standard QC of genotype data was applied, with SNPs filtered by sample call rate > 95%, MAF > 1%, SNP call rate > 95%, HWE $P \leq 1\times10^{-4}$. In total, 332,849 SNPs passed these criteria. Imputation was done with Michigan imputation server using Eagle and Minimac software. HRC reference panel was used for imputation of samples with European descent. The GRCh37 human genome build was used.

QMDiab

Genotyping was carried out using Illumina Omni array 2.5 (version 8). Standard quality control of genotyped data was applied, with SNPs filtered by sample call rate > 98%, MAF > 1%, SNP call rate: > 98%, HWE $P \leq 1 \text{ x } 10^{-6}$. In total 1,223,299 SNPs passed criteria. Imputation was done using SHAPEIT software with 1000G phase 3 version 5 and mapped to the GRCh37 human genome build. Imputed SNPs were filtered by imputation quality > 0.7, leading to 20,483,276 SNPs passed to the GWAS analysis.

CEDAR

Total DNA was extracted from EDTA-collected peripheral blood using the MagAttract DNA blood Midi M48 Kit on a QIAcube robot (Qiagen). DNA concentrations were measured using the Quant-iT Picogreen ds DNA Reagents (Invitrogen). Individuals were genotyped for >700 K SNPs using Illumina's Human OmniExpress BeadChips, an iScan system and the Genome Studio software following the guidelines of the manufacturer. We eliminated variants with call rate ≤0.95, deviating from Hardy–Weinberg equilibrium $P \leq 1\times10^{-4}$, or which were monomorphic. We confirmed European ancestry of all individuals by PCA using the HapMap population as reference. Using the real genotypes of 629,570 quality-controlled autosomal SNPs as anchors, we used the

Sanger Imputation Services with the UK10K + 1000 Genomes Phase 3 Haplotype panels (https://imputation.sanger.ac.uk) to impute genotypes at autosomal variants in our population.

Phenotyping

Plasma N-glycome quantification

Plasma N-glycome quantification of samples from the TwinsUK, EPIC-Potsdam, PainOmics, SABRE, QMDiab and CEDAR studies was performed at GENOS by applying the following protocol. Plasma N-glycans were enzymatically released from proteins by PNGase F, fluorescently labeled with 2-aminobenzamide and cleaned-up from the excess of reagents by hydrophilic interaction liquid chromatography solid phase extraction (HILIC-SPE) as described previously [23]. Fluorescently labeled and purified N-glycans were separated by HILIC on a Waters BEH Glycan chromatography column, 150 × 2.1 mm, 1.7 µm BEH particles, installed on an Acquity ultra-high-performance liquid chromatography (UHPLC) instrument (Waters, Milford, MA, USA) consisting of a quaternary solvent manager, sample manager and a fluorescence detector set with excitation and emission wavelengths of 250 nm and 428 nm, respectively. Following chromatography conditions previously described in detail [23], glycan peaks (GPs) – quantitative measurements of glycan levels – were defined by manual integration of intensity peaks in the chromatograms for PainOmics retrospective study (Italian subcohort) and for SABRE cohort, while for other PainOmics subcohorts and EPIC-Potsdam GPs were defined by automatic integration [24]. The number of defined GPs varied among studies from 36 to 42. The abundance of N-glycans in each chromatographic peak was expressed as percentage area of the corresponding peak.

Plasma N-glycome quantification for SOCCS samples was done at NIBRT by applying the same protocol as for EPIC-Potsdam, PainOmics, and SABRE with the only difference in the excitation wavelength (330 nm instead of 250 nm).

Harmonization of glycan peaks

Depending on the cohort, some neighboring chromatographic peaks were not well separated due to differences between used UHPLC column batches and were consequently quantified as one chromatographic peak. To conduct association analysis in four cohorts followed by meta-analysis, we harmonized the set of GPs by applying a recently published protocol[25]. In short, based on this table of correspondence between glycan peaks across cohorts (see Supplementary Table 17), we created a harmonization scheme. Applying this procedure resulted in a harmonized set of 36 GPs for all samples.

Normalization and batch-correction of GPs

Normalization and batch-correction were performed on harmonized UHPLC glycan data separately for all cohorts using the protocol we have published recently[26]. We used probabilistic median quotient normalization [27,28]. This approach is based on the calculation of the dilution factor of each sample with respect to a reference sample. The reference sample was calculated as the median value of the abundance of each GP across all measured samples. For each sample, a vector of quotients was then obtained by dividing each GP measurement by the corresponding value in the reference sample. The median of these quotients was used as the sample's dilution factor, and

the original sample values divided by that value. The underlying assumption is that the different intensities observed across individuals relate to different amounts of the biological material in the collected samples.

Normalized glycan measurements were log-transformed due to the right skewness of distribution and multiplicative nature of batch effects. Before the batch correction, outlying measurements were removed. Outlier was defined as a sample that had at least one GP that are greater than upper quartile + 3 * IQR (interquartile range) or lower than lower quartile - 3 * IQR. The batch correction was performed on log-transformed measurements using the ComBat method[29], where the technical source of variation (batch and plate number) was modeled as a batch covariate. Again, samples with outlying measurements were removed.

From the 36 directly measured glycan traits, 81 derived traits were calculated (see Supplementary Table 1). These derived traits average glycosylation features such as branching, galactosylation, and sialylation, etc across different individual glycan structures and, consequently, they may be more closely related to individual enzymatic activity and underlying genetic polymorphism. As derived traits represent sums of directly measured glycans, they were calculated using normalized and batch-corrected glycan measurements after transformation to the proportions (exponential transformation of batch-corrected measurements).

Univariate genome-wide association analysis

The protocols of association analysis and meta-analysis were created based on the published protocol[30]. Prior to analyses, each cohort received quality controlled glycan data (glycan data were preprocessed by the phenotype provider) and an analysis plan for genome-wide association studies. Samples of some cohorts were split before running the association analysis to avoid effect of potential confounding factors: a case/control status, different SNP-array used for genotyping, different descent of samples. We split samples of SABRE cohort by European of South Asian descent; samples of PainOmics cohort by SNP-array (Illumina GSA and Human Core) and by descent (Croatian, Belgian, British and Italian), samples of EPIC-Potsdam by SNP-array (Human660W-Quad, HumanCoreExome and InfiniumOmniExpressExome); samples of SOCCS cohort by colorectal cancer cases and controls. Glycome measurement for samples of the TwinsUK cohort were done in two batches with differences in few years and therefore, were split in two subcohorts.

Before the association analysis, the total plasma N-glycome traits were adjusted for sex, age and cohort specific covariates. Residuals were quantile transformed to normal distribution (rntransform function in the GenABEL v. 1.8-0 [31,32] R package). GWAS were performed on corrected glycan data and imputed genotypes, considering relatedness and population stratification and assuming an additive model of association.

We performed quality control (QC) for each of 117 individual-level GWAS summary statistics per cohort based on a protocol, suggested by Winkler *et al*[30], implemented in GWAS-MAP platform[33]. As a part of QC, we removed non-informative and SNPs of low imputation quality (imputation quality < 0.7). We kept SNPs with MAF above 1%. As an additional step of QC, we examined the consistent of the effect of three known glyQTLs - *ST6GAL1*, *ST3GAL4* and *FUT8*, that showed most significant association with N-glycome traits

as reported in previous round of blood plasma N-glycome GWAS[26]. Effects of this glyQTLs were consistent among all individual GWASs even with the smallest sample sizes of 150 samples.

Discovery and replication genome-wide association meta-analysis

For each trait cleaned set of individual GWAS summary statistics was meta-analysed using fixed effect inverse-variance method implemented in GWAS-MAP platform[33]. We calculated GWAMA summary statistics for each trait for discovery and replication samples. Additionally, for the purpose of post-GWAS analyses, we generated GWAMA summary statistics for samples of European descent. GWAMA summary statistics passed the same QC procedure. In total, we report results of association analysis for 117 traits and 9,772,134 SNPs.

Locus definition

We used the following procedure to define the associated loci and strongest SNP - trait associations:

1) For each glycan trait repeat the following:
   a) Remove all SNPs with p-values higher that given threshold.
   b) Select the SNP with lowest p-value (top-SNP).
   c) Remove all SNPs within 500 Kb window (+-250 Kb from this top-SNP including top-SNP).
2) Repeat while we have SNPs with p-value lower than the given threshold.
3) Formulate the joint list of all SNP - trait pairs for all 117 GP.
4) Repeat the following:
   a) Select the top-SNP - trait pair with lowest p-value.
   b) Remove all SNP-trait pairs within 1000 Kb window (+-500 Kb from top-SNP); add the names of removed associated traits into the list of associated traits for this locus.
   c) Repeat while we have SNP- trait pairs.
   d) Formulate the list of top-SNP - trait pairs.
5) All SNP-trait pairs located within 1 Mb from each other were checked manually by regional association plots, in case of similar pattern of associations only the SNP-trait pair with lowest p-value was selected.

Multivariate genome-wide association analysis

Phenotype grouping for multivariate analysis

Following the study by Shen et al.[34] and by Shadrina et al.[35], we grouped 36 directly measured total plasma N-glycan traits (described in Supplementary Table 1a) into twenty-one groups (described in Supplementary Table 1b) based on the biochemical similarities of glycans, including one group that combined all 36 total plasma N-glycosylation traits. We composed four multivariate traits on the basis of the number of antennary branches. Glycans with two antennas were included in the "LB" trait (*level of biantennary glycans*), and glycans with three or four antennas were included in the "HB" trait (*highbranching glycans*). Also, to separate highbranching

glycans we considered three- and tetra- antennary structures as "HB3" (*level of triantennary glycans*) and "HB4" (*level of tetraantennary glycans*) multivariate traits. The general fucosylation level of the N-glycans core formed the trait "FUC-C" (*core-fucosylation*) and the overall level of fucosylation of the antennary chains - "FUC-A" (*fucosylation of antennary branches*) respectively. Glycans with additional GlcNAc between branches constructed the multivariate trait «B» (*bisecting GlcNAc*), glycans with a large number of mannose residues in their composition – trait "OM" (*oligomannose structures*). The presence of galactose and sialic acid monomers in the composition of glycans was considered as follows: if there was one galactose (sialic acid) monomer in the glycan, it belonged to the multivariate trait "G1 (S1)" (*monogalactosylation* (*monosialylation*) *of antennary branches*), two monomers – "G2 (S2)" (*digalactosylation* (*disialylation*) *of antennary branches*), three and four –"G3 (S3)" (*trigalactosylation* (*trisialylation*) *of antennary branches*), "G4 (S4)" (*tetragalactosylation* (*tetrasialylation*) *of antennary branches*). And, conversely, if there was no galactose (sialic acid) monomer in the glycan structure, glycan was attributed to the "G0 (S0)" trait (*agalactosylation* (*neutral glycans*)). In addition, all N-glycans having at least one galactose (sialic acid) in their structure made up the trait "G (S)" (*galactosylation* (*sialylation*)).

Discovery multivariate analysis

The multivariate analysis was performed using the summary statistics-based method described by Ning et al.[36]. The method enables multivariate analysis of variance (MANOVA) using the results of multiple GWAS for individual traits. Calculations were performed using the MultiABEL R package (v. 1.) (`MultiSummary` function, `type = “outbred”`)[34,36] on summary statistics of discovery meta-analysis filtered by condition $N > 0.9N_{max}$, where N – the number of samples. The statistical significance threshold for multivariate analysis was set at $P \leq 5.0 \times 10^{-8}/21$, where 21 is the number of multivariate traits.

Associated loci were defined as ±250 Kbp-vicinities of the SNPs most significantly associated with N-glycosylation multivariate trait groups (lead SNPs). SNPs located between 25 and 35 Mb on the chromosome 6 were merged into a single MHC locus. Additionally, for the neighboring loci, regional association plots were manually inspected, and the loci were merged if they fell into one LD block.

Replication

For the replication we used the protocol, published by [37]. Since there is no direct trait-to-trait correspondence between glycan traits measured by HPLC and UHPLC technologies, we tested the association of tag SNPs with all 117 glycan traits. We considered a locus as replicated if the tag SNP showed association with at least one of 117 glycan traits with a replication threshold of $P \leq 0.05/(16*117) = 2.67\times10^{-5}$, where 16 is the number of tag SNPs and 117 is the number of N-glycosylation traits. The summary of the association between 16 loci and glycome traits passed replication threshold is provided in Supplementary Table 3b.

The most common approach to replicate found loci in GWAS is to perform the same test that was performed at the discovery stage on replication cohorts. But in case of testing for associations with multivariate traits such straightforward approach fails to provide evidence for the consistency of the effect between two samples and for its pleiotropy. Therefore, we used a complex four-level replication strategy as proposed by Ning et al[36], which consists of the following steps: MANOVA, Phenotype Score, Pearson correlation method and Kendall correlation method.

In the first stage (MANOVA) we straightforwardly checked whether the locus is still associated with the multivariate trait in the replication cohort using the same test as in the discovery stage; then we checked whether the effect direction is consistent between the two cohorts, using phenotype score approach; next we evaluated the concordance of multivariate effect between two samples using Pearson's and Kendall's correlation coefficients. We considered a locus replicated if it had successfully passed MANOVA and phenotype score steps of replication, and the multivariate effect of the locus replicated if it additionally had passed both Pearson's and Kendall's correlation steps of replication.

MANOVA-test has been performed similarly to described in Discovery section using $P \leq 0.0021$ (0.05 / (7 + 16), where 7 is the number of previously identified but not replicated loci and 16 is the number of novel loci).

Phenotype score-based replication was performed similarly to the one described in Shadrina et al[35]: for each lead SNP-top trait group pair coefficients of the linear combination of genotype onto multiple atomic phenotypes were extracted for the discovery cohort (the secondary output from the `MultiSummary` function) and used for constructing the corresponding trait group phenotypes and then association between the lead SNPs and the constructed linear combinations were tested as $\left(\frac{\beta_{new}}{se_{new}}\right)^2 \sim \chi_1^2$, where

$$\beta_{new} = \frac{B * a}{\sqrt{sum\left(\sum_{ph} * (a \otimes a)\right)}},$$

$$SE_{new} = \frac{\sqrt{\left(SE_1^2 + \frac{\beta_1^2}{N}\right) * sum\left(\sum_{ph} * (a \otimes a)\right) - \frac{(B * a)^2}{N}}}{\sqrt{sum\left(\sum_{ph} * (a \otimes a)\right)}},$$

where B—matrix, where each column β_i—effect of each SNP in $GWAS_i$; a—vector of linear combination coefficients estimated in the discovery cohort; $\sum_{ph}$ — matrix of correlations between 36 total plasma N-glycosylation traits in the replication cohort, ⊗—outer product; SE_1 —standard error of each SNP's effect in the first $GWAS_1$; N—minimal N out of all GWASes for each SNP.

A locus was considered to be replicated if the association of the SNP with the constructed linear combination had the same direction of effect as in the discovery cohort and passed the threshold of $P < 0.0021$.

To evaluate the similarity between estimates of multivariate genetic effects from discovery and replication cohorts across multiple traits, we used MC-based approach implemented in MultiABEL package (`MV.cor.test` function)[34]. For both Pearson's and Kendall's correlation coefficients we consider a multivariate effect for a specific SNP replicated if the 95% confidence intervals didn't include zero.

Statistical power calculation

For each SNP, statistical power (or probability) of replication was estimated using the fact that under the alternative hypothesis (H1: $\beta \neq 0$) the test statistics T^2 from the replication sample is expected to follow the $\chi^2_{df=1,NCP}$ distribution, where NCP is the expected non-centrality parameter computed as $(T^2 - 1) * N_{rep}/N_{disc}$, where $T^2 = (\beta_{disc}/se_{disc})^2$ is the test statistic for

a particular SNP in the discovery cohort, N_{rep} is the sample size of the replication cohort and N_{disc} is the sample size of the discovery cohort. The power of replication is equal to the probability that the statistics following such a distribution would exceed the threshold value $k = 9.8$ that corresponds to right-hand integral of $\chi^2_{df=1}$ equal to $0.05/28 = 1.79 \times 10^{-3}$.

Trait selection for the colocalization-analysis

We used traits, summary statistics for which were available from the GWAS-MAP database, as secondary traits in SMR/HEIDI analysis. For the "disease-related" group of traits we primarily included traits from the UK Biobank's inpatient admissions diagnoses data (data field 41202 – Diagnoses - ICD10) as provided by Gene Atlas and Neale Lab. From this set, we excluded traits that correspond to external causes, and those that do not directly correspond to the disease. We had additionally included UK Biobank's summary statistics for cancer diseases self-reported during the verbal interview, expanding the group with additional unique phenotypic endpoints. The motivation for this is the fact that associations between N-glycosylation levels and some of the cancer disease had already been demonstrated. Additionally, we expanded the set by summary statistics for touchscreens and questionnaires (category 100044 – Medical conditions, category 132 – Medical information), regarding medical diagnoses, since some of these are characterized by a greater effective sample size and, therefore, higher power, and summary statistics from another sources, including cardiovascular diseases from the CARDIoGRAM Consortium, psychiatric traits from the Psychiatrics Genomics Consortium and some others (see Supplementary Table 9a).

For the "complex trait" group, we included data on psychometric and anthropometric traits from the UK Biobank, metabolomic trait data from the Global Lipids Genetics Consortium and {NMR metabolomics} and other complex traits (see Supplementary Table 9a).

Due to the fact that some of the GWASs tested the association of the same trait on overlapping samples (for example, GWASs from the Gene Atlas' UK Biobank collection and Neale Lab's UK Biobank collection), we additionally applied a deduplication procedure. In case of "disease-related" traits we had assigned three- or four-character ICD-10 codes for each trait, with the possibility of having several codes for one trait. In case of UK Biobank's inpatient admissions diagnoses data, those codes had already been provided, in the rest of the cases we manually assigned codes based on the matching of the common trait name with the descriptions of the ICD-10 codes. Secondly, if two studies had exactly the same set of codes while having a significant sample overlap, we left only one study with the greatest effective sample size in the analysis.

In case of the "complex trait" group we performed matching based on the trait names only. We marked a pair of studies as duplicates if either both names were completely matched lexicographically or one of the names was completely contained in the other, or had very similar semantics, as determined by manual curation. Then for each set of duplicates, only one study had been left based on the sample size.

Classification of glycans into Ig-related and non-Ig

We classified all glycan traits (including derived traits) into four groups: Ig-related, non-Ig-related, mixed, and glycans without assignment (NA).

For original traits (36 glycans) we extracted information about the proportion of Ig-linked glycans within specific chromatographic peak from the work[38]. We denote this coefficient as R_i^{Ig}, where i – is a particular glycan trait. For six chromatographic peak this information was not available (NA). We considered a trait to belong to Ig-related class if R_i^{Ig} was more than 80%. We considered a trait to belong to non-Ig class if R_i^{Ig} was less than 20%. Otherwise, we considered the trait to be “mixed”.

For classification of derived traits we performed next procedure:

1) Each derived trait (DT) is a weighted linear combination of original traits

$$DT = \sum_{i=1}^{36} GP_i * W_i$$

or ratio of weighted linear combinations of original traits:

$$DT = \frac{\sum_{i=1}^{36} GP_i * W_i}{\sum_{j=1}^{36} GP_j * W_j}$$

where GP_i is a level of particular glycan and W_i is its weight (0 or 1)

2) For nominator and denominator, we computed the score of relatedness to Ig or non-Ig proteins using formula:

$$Score_{numerator}^{Ig} = \sum_{i=1}^{36} \overline{GP_i} * W_i * R_i^{Ig}$$

$$Score_{denominator}^{Ig} = \begin{cases} \sum_{j=1}^{36} \overline{GP_j} * W_j * R_j^{Ig}, & if\ DT\ is\ a\ ratio \\ 0, & if\ DT\ is\ not\ a\ ratio \end{cases}$$

where $\overline{GP_i}$ is a mean value of particular glycan in the plasma (estimated on 2763 samples from TwinsUK cohort). The original traits without available information for R_i^{Ig} were considered as glycan trait with $R_i^{Ig} = 0$.

3) The total score was computed as mean of scores for nominator and denominator.

$$Score_{Ig} = \frac{Score_{numerator}^{Ig} + Score_{denominator}^{Ig}}{2}$$

4) We considered derived trait to belong to Ig-related class if $Score_{Ig}$ was more than 80%. We considered derived trait to belong to non-Ig class if $Score_{Ig}$ was less than 20%. If the $Score_{Ig}$ of derived trait was between 20% and 80% - we considered it as “mixed”.

Systematic prioritization of effector genes for protein glycosylation

Glycosyltransferases

In ten loci we prioritized ten genes - *FUT8, FUT6, ST6GAL1, ST3GAL4, ST3GAL6, B4GALT1, MGAT5, MGAT3, ABO, B3GAT1* - encoding glycosyltransferases - a group of enzymes that catalyze transfer of sugar molecules (such as fucose, galactose, or sialic acid) from activated donor molecules (such as UDP-glucose or CMP-sialic acid) to acceptor molecules (proteins, lipids, or other sugars) to form glycosidic bonds. We showed the colocalization of found glyQTLs with expression levels of FUT8 in CD14+ monocytes, ST3GAL4 in liver, B3GAT1 in liver and peripheral blood and MGAT3 in CD19+ B lymphocytes and CD8+ T lymphocytes (Supplementary Table 7e). The mRNA levels of these genes were positively correlated with the levels of N-glycan-products of the corresponding biochemical reactions (Supplementary Table 7e).

We observed consistency between known enzymatic activities of the products of eight genes and the spectrum of glycans that were associated with corresponding loci. Fucosyltransferases *FUT8* and *FUT6* were associated with the levels of core and antennary fucosylation, sialyltransferases *ST6GAL1, ST3GAL4 and ST3GAL6* were associated with the levels of sialylation of biantennary and sialylation of highly branched glycans. *ST3GAL6* was associated with the level of highly branched N-glycans, which are at the same time heavily sialylated, galactosyltransferase *B4GALT1* was associated with galactosylation of N-glycans, *N*-acetyl-glucosaminyltransferases *MGAT5* and *MGAT3* were associated with the levels of highly branched and bisected N-glycans. Association of two glycosyltransferase loci - *ABO* and *B3GAT1* – with their spectrum of N-glycan features was less interpretable.

The *ABO* gene is one of the most important genes in determining an individual's blood type. The A allele produces α-1,3-*N*-acetylgalactosamine transferase which transfers GalNAc residues from the UDP-GalNAc donor nucleotide to the Gal residues of the acceptor H antigen, while the B allele produces α-1,3-galactosyl transferase that transfers Gal residues from the UDP-Gal donor nucleotide to the Gal residues of the acceptor H antigen. These *N*-acetylgalactosamine-Gal or Gal-Gal linkages are not known to be presented in the N-glycome of plasma proteins but only on erythrocytes. Accordingly, the AB0 glyQTL index SNP rs582118 (A/G) is in high LD (R^2=86%, EUR samples from 1000 Genomes, p3v5) with rs8176719 (-/C), often referred to as c.261delG (minus strand) which is a key SNP in determining blood group type O status (allele with deletion)[39]. The rs582118-A, which is in LD with c.261delG, is associated with increased level of PGP9, which is formed by two major glycans: high mannose immature M7 and biantennary digalactosylated monosialylated A2G2S1, none of which contain *N*-acetylgalactosamine-Gal or Gal-Gal linkages. The evidence for the gene *ABO*, which is associated with variation in plasma N-glycosylation, is somewhat contradictory. On the one hand, the evidence supporting this gene is strong (three predictors): the candidate encodes a glycosyltransferase; its role is reinforced by containing variants in strong LD ($r^2 \geq 0.8$) with the protein-truncating variant (annotated by Variant Effect Predictor, VEP[40]) and it is predicted to be damaging by FATHMM XF[41], FATHMM InDel[42]; and it is also the nearest gene (see Supplementary Table 7a).

The *B3GAT1* gene encodes beta-1,3-glucuronyltransferase 1, which transfers glucuronic acid to galactose-*N*-acetylglucosamine making human natural killer-1 (HNK-1) carbohydrate epitope, which is highly expressed in nervous system. We demonstrated the pleiotropic effect of *B3GAT1* glyQTL on expression of B3GAT1 gene in liver and peripheral blood. The *B3GAT1* glyQTL is associated with A4F1G4S4 and A4G4S3, which are tetra antennary heavily galactosylated and sialylated N-glycans, attached to liver secreted proteins, including alpha-1-acid protein[38]. Alpha-1-acid protein, also known as orosomucoid, is a major acute-phase protein that is involved in the regulation of inflammation and immune responses. It is heavily glycosylated, with up to 4 glycan chains per protein molecule. Additionally, the *B3GAT1* glyQTL showed association with A3G3S2, which is attached to haptoglobin, which is encoded by HP gene, and beta-2-glycoprotein, found in the present study. The presence of glucuronic acid in the mentioned N-glycans has not yet been shown, therefore, the precise role of B3GAT1 in the regulation of these specific N-glycan structures remains unclear.

The *CHST2* gene encodes *N*-acetylglucosamine-6-*O*-sulfotransferase (GlcNAc6ST)-1. GlcNAc6ST-1 catalyzes the transfer of a sulfate group from the substrate 3′-phosphoadenosine 5′-phosphosulfate (PAPS) to an *N*-acetylglucosamine residue located at the nonreducing end of glycans[43]. The enzyme is localized in the Golgi. There is no evidence yet of sulfate group being part on N-glycome of human blood plasma proteins.

N-glycoproteins

We prioritized the genes encoding N-glycoproteins in two glyQTLs, *HP*/*HRP* and *SERPINA1*, namely haptoglobin (Hp) and alpha-1 antitrypsin. The *HP* glyQTL is associated with various N-glycan structures, some of which are linked to the Hp protein. The glyQTL is colocalized with Hp pQTL, indicating that genetic variants in the HP locus affect N-glycosylation of Hp through the level of Hp in the blood. The Haptoglobin receptor (*HPR*) gene was also prioritized due to its proximity to index SNP rs217184.

Alpha-1 antitrypsin (AAT) is an acute phase protein that regulates inflammation[44]. Mutations in the *SERPINA1* gene can cause AAT deficiency, which increases the risk of lung and liver disease[45]. The rs28929474 index SNP of this glyQTL is a well-known PL*Z allele, leading to Glu366Lys substitution (1.8% frequency in European populations) in protein sequence, which is a leading genetic factor of AAT deficiency[46]. Glu366Lys is negatively associated with the abundance of A2G2S2 ($beta = -0.43\ SD$), the most abundant N-glycan of AAT. This suggests that the *SERPINA1* glyQTL affects the A2G2S2 N-glycan abundance through the effect of Glu366Lys on the level of AAT in blood. However, the *SERPINA1* glyQTL and AAT pQTL do not colocalize, likely due to the low frequency of Glu366Lys. Nonetheless, rs28929474 is associated with the level of glycoprotein acetyl, a mixture of N-glycoproteins, predominantly alpha-1-acid glycoprotein, haptoglobin, and alpha-1-antitrypsin[47].

Transcription factors

In 12 glyQTLs, we prioritized genes encoding transcription factors: previously implicated *RUNX3*, *HNF1A*, *IKZF1*, *SMARCB1*, *ELL2*, *MEF2B*, *HIVEP2*, *RUNX1* and newly found *SMARCD3*, *ATF6B*, *MAX*, *BCL7B*, *MLXIPL*. These transcriptional factors may regulate the

expression of glycosyltransferases and N-glycoproteins (see Tissue specific regulation of protein N-glycosylation).

The direct role of *RUNX3*[48] and *IKZF1*[49] in the regulation of IgG N-glycosylation, and *HNF1A*[50] in the regulation of total plasma N-glycome was confirmed experimentally in previous studies. Briefly, the *HNF1A* affects protein fucosylation through the regulation of expression of fucosyltransferases, including *FUT6* and *FUT8* in liver-derived cells, implicated in the present study, *IKZF1* affects core-fucosylation of proteins through the regulation of expression of *FUT8* in B-lymphocytes-derived cell line and *RUNX3* affects galactosylation of proteins through the regulation of *B4GATL1* in HEK-293FS cell line. The RNA polymerase II transcription elongation factor *ELL2* is involved in the expression of immunoglobulin heavy-chain complex (IgH) in plasma cells[51]. Lowering *ELL2* expression results in lower abundance of secretory-specific forms of immunoglobulin heavy-chain mRNA, suggesting that *ELL2* can affect the plasma N-glycome through the level of immunoglobulins (the most abundant N-glycoproteins in plasma).

MAX is a member of the basic helix-loop-helix leucine zipper (bHLHZ) family of transcription factors. It can form homodimers and heterodimers with other family members, which include Mad, Mxi1 and Myc. Myc is an oncoprotein implicated in cell proliferation, differentiation and apoptosis. The *MYRF* gene encodes a transcription factor that is required for central nervous system myelination and may regulate oligodendrocyte differentiation. It is thought to act by increasing the expression of genes that effect myelin production but may also directly promote myelin gene expression.

Membrane transporters

In three loci we have prioritized genes encoding membrane transporters: previously implicated *SLC9A9* and newly found *SLC39A8, CALB2*. *SLC39A8*[52] deficiency causes a type II CDG[53]. The glycosylation disorder is believed to arise due to reduced functioning of the β-galactosyltransferase enzyme, which requires manganese as a cofactor[52,53]. β-galactosyltransferase transfers UDP-galactose to *N*-acetylglucosamine of a glycan. Hence, manganese deficiency inhibits this process resulting in reduced glycosylation, as evidenced by the reduced levels of the most common tetrasialo-form of transferrin [52,53].

The prioritized gene *CALB2* encodes an intracellular calcium-binding protein. This protein plays a role in diverse cellular functions, including message targeting and intracellular calcium buffering. Calcium ions are essential for the function of the lectin-like chaperones calnexin and calreticulin which are known[54] to be involved in N-glycoprotein folding and in the quality control of misfolded glycoproteins in the endoplasmic reticulum.

The *SLC9A9* gene encodes a sodium/proton exchanger involved in pH regulation in the endosomal compartment[55]. Rivinoya and colleagues[56] showed that elevated pH in the Golgi apparatus can impair terminal protein N-glycosylation, especially sialylation. Previously, Sharapov and colleagues[26] suggested that associations between the *SLC9A9* gene and N-glycosylation may be due to changes in the pH level on the Golgi scale. This suggestion is consistent with current results: the strongest associations between *SLC9A9* and univariate glycan traits are seen for the incidence of bisecting GlcNAc in all fucosylated disialylated structures and

for the ratio of core-fucosylated disialylated structures with and without bisecting GlcNAc. Among the multidimensional traits, total N-glycosylation, galactosylation, and sialylation are most strongly associated, which is also consistent with the hypothesis of an effect on glycosylation through a change in the pH level on the Golgi scale.

Lipid and glucose metabolism

For five glyQTLs we have prioritized genes involved in and associated with lipid metabolism regulation - *GCKR, HNF1A, MLXIPL, TRIB1, FADS2*. The *GCKR* gene is known to be associated with elevated plasma triglycerides levels, type 2 diabetes mellitus, familial combined hyperlipidemia and nonalcoholic fatty liver disease[57]. The product of the *GCKR* gene is a regulatory protein which inhibits glucokinase in liver cells by binding non-covalently to form an inactive complex with the enzyme. The T allele of a common genetic polymorphism rs1260326, the lead SNP in the present study, encodes *GCKR* that responds less adequately to fructose-6-phospate, a natural facilitator of *GCKR*-glucokinase complex, which leads to the equilibrium shift towards the dissociation of the complex, and as a consequence to an increase in glucokinase activity, glucose phosphorylation, and consequently, pyruvate production in hepatocytes. It is anticipated that increased cytosol pyruvate levels augment glycogen deposition and *de novo* lipogenesis. The latter explains the positive association with elevated triglycerides, fatty acids levels and livers steatosis[57]. A glucose-activated transcription factor *MLXIPL* also promotes lipogenesis by trans-activating key enzymes needed for the process[58], and therefore stimulates the deposition of fat in the liver. In turn, *TRIB1* pseudokinase downregulates hepatic lipogenesis and glycogenesis via multiple molecular interactions. *TRIB1* is required for the function of the hepatic master-regulator *HNF4A*, the deficiency of which causes impurity of liver integrity and lipid metabolism in mice[59,60], moreover, the latter protein directly controls regulation of the fat amount stored in the liver in mice[61]. It is also of interest, that *TRIB1* has been shown to interact with *MLXIPL*, which indicates multimodality of lipid metabolism regulation by *TRIB1* in hepatocytes[62]. In turn, one of the observed consequences of the liver steatosis is an increase in plasma C-reactive protein levels, which is a known glycoprotein[63]. This association possibly accounts for the association between the rs1260326's T allele in *GCKR* and plasma C-reactive protein levels[64,65], and thus it is likely that these loci contribute to N-glycosylation variation via liver steatosis-induced C-reactive protein secretion into the bloodstream.

Polymorphisms in the *HNFA1* gene also have been associated with variations of low-density lipoprotein and plasma levels of C-reactive protein. Additionally, it has been shown that HNF1A-null mice have enlarged fatty livers and impaired catabolism and transport of fatty acids[66,67], which may suggest an additional mechanism for association with levels of N-glycosylation via increase in CRP secretion into the bloodstream due to liver steatosis.

Another intriguing locus which possibly contributes to a treasury of steatosis association is FADS2, covering a cluster of 3 fatty acid desaturase genes *FADS1, FADS2, FADS3*. FADS1 and FADS2 activity and genetic polymorphisms in the region are associated with liver steatosis[68–70], however the mechanisms are still to be revealed, and these findings may be a consequence of the association of the desaturase activity with lipid composition and obesity[71]. Nevertheless, there are

existing highlights from mice that FADS2-deficient phenotypes are characterized by perturbed liver lipidogenesis. FADS2-deficiency caused lipid bilayer alterations interfere with maturation of microsomal form of SREPB-1c into the nuclear form[72]. SREPB-1c is a transcriptional factor responsible for expression of genes involved in fatty acid biosynthesis and thus its function deficiency attenuates lipidogenesis and liver steatosis[58,72], which in turn can decrease CRP plasma levels.

Association between PGS for plasma N-glycosylation traits and ICD-10 diseases

The full results of the association analysis can be found in Supplementary Table 10. Statistically significant associations are as well depicted in Fig. 4. The threshold for statistical significance was set at $1.07 \times 10^{-5} = 0.05/(28 * 167)$, where 28 is the number of plasma N-glycome principal components explaining over 99% of the 117 N-glycosylation traits variation, and 167 is the numbers of ICD-10 codes.

Disease phenotypes, significantly associated with N-glycosylation of plasma proteins

Disorders of lipoprotein metabolism and other lipidaemias (E78)

Disorders of lipoprotein metabolism were statistically significantly associated with PGS for 18 plasma N-glycosylation traits, which is the biggest number of associations observed for the studied diseases. Interestingly, four out these 18 PGS are for the N-glycan traits that describe the high-mannose content of the plasma N-glycome, such as M9, M7 N-glycan species as well as high-mannose N-glycans in general, and PGS for these N-glycan traits are all positively associated with E78 disease phenotypes.

We also noticed that disorders of lipoprotein metabolism and other lipidaemias (E78) and essential (primary) hypertension (I10) share a number of associations with PGS for certain N-glycan traits. Both disorders are negatively correlated with PGS for PGP94 (S0total, the percentage of neutral glycan structures in total plasma glycans) and PGP117 (Gntotal, the percentage of galactosylated structures in total neutral plasma glycans) and positively associated with the PGS for PGP23 (the percentage of A3G3S2), PGP69 (M9n, the percentage of M9 in total neutral plasma glycans) and PGP18 (the percentage of M9). The positive association between E78 (lipidaemias) and PGS for PGP18 was the strongest association observed in our dataset ($P = 1.06 \times 10^{-22}$).

Cardiovascular diseases

Essential (primary) hypertension (I10) was statistically significantly associated with PGS for 11 plasma N-glycosylation traits. The strongest positive association ($P = 1.06 \times 10^{-10}$) for essential hypertension was observed with the PGS for the percentage of M9 in total neutral plasma glycans (PGP18, M9). Essential hypertension was also positively correlated with PGS for abundance of M9 high-mannose glycan in the total neutral glycome (PGP69, M9n). These two positive associations with PGS for abundances for high-mannose structures were also statistically significant for two other cardiovascular diseases in the analysis, angina pectoris (I20) and chronic ischemic heart disease (I25).

PGS for triantennary sialylated structures (PGP23, A3G3S2; PGP29, A3G3S3), sialylation of core-fucosylated glycans without bisecting *N*-acetylglucosamine (PGP37, FGS/(FG+FGS))

were positively correlated with essential hypertension. Negative correlations were observed for essential hypertension and PGS for neutral and/or galactosylated structures (PGP94, S0total; PGP100, G1total; PGP117, Gntotal), for the ratio of fucosylated monosialylated and disialylated structures without bisecting *N*-acetylglucosamine (PGP47, FS1/FS2) and for the monogalactosylated bisected N-glycan A2BG1 (PGP3).

Interestingly, essential hypertension shares associations for PGS for six glycan traits with disorders of lipoprotein metabolism and other lipidaemias, for four traits - with type 2 diabetes, and for three traits with obesity.

Non-insulin-dependent diabetes mellitus (E11)

Non-insulin-dependent diabetes mellitus (E11) or type 2 diabetes was strongly positively associated with PGS for seven glycan traits, the most significant association being with PGS for PGP37 (FGS/(FG+FGS), P = 1.70 x 10^{-9}), the percentage of sialylation of core-fucosylated galactosylated structures without bisecting *N*-acetylglucosamine. Other N-glycan PGS that were statistically significantly positively associated with this disease phenotype group include PGS for sialylation of all core-fucosylated structures without bisecting *N*-acetylglucosamine (PGP39, FGS/(F+FG+FGS)), for triantennary trisialylated N-glycan A3G3S3 (PGP29) and for the percentage of M9 in total neutral plasma glycans (PGP69, M9n). Negative association with non-insulin-dependent diabetes mellitus were observed for PGS for two traits connected with low abundance of sialylated structures (PGP94, S0total; PGP117, Gntotal) and for PGP17, describing the percentage of the A2G2S2 glycan in the plasma N-glycome. Some of these associations were shared with obesity (E66), disorders of lipoprotein metabolism (E78) and primary hypertension (I19).

Obesity (E66)

Obesity (E66) was statistically significantly associated with PGS for six plasma N-glycosylation traits. In particular, it was negatively associated with PGS for four traits, describing increase in galactosylation, such as G1total (PGP100), Gntotal (PGP117), FA2G1 (PGP5) and G1n (PGP71). Negative association with Gntotal is shared with disorders of lipoprotein metabolism and other lipidaemias, with G1n – with unspecified diabetes (E14). Moreover, obesity was also negatively associated with S0total (PGP94), sharing this association with type 1 diabetes and disorders of lipoprotein metabolism and other lipidaemia, and positively with PGS for FBG2n/FG2n (PGP88), that is related to increased bisection.

Behavioural disorders due to use of tobacco (F17)

Tobacco use disorders (F17) were associated with PGS for six plasma N-glycosylation traits, four of which were related to increased abundance of bisected N-glycans (PGP59, FA2Bn; PGP82, FBG1n/G1n; PGP84, FBn/Fn; PGP85, FBn/Fntotal). This disease was also negatively associated

with PGS for PGP76 (Fn, the percentage of fucosylated structures without bisecting GlcNAc in total neutral plasma glycans) and PGP5 (FA2G1).

Asthma (J45)

Asthma (J45) was positively associated with PGS for five plasma N-glycosylation traits, out of which four describe the abundances of high-mannose structures (PGP18, M9; PGP64, M6n; PGP69, M9n; PGP107, Mtotal). It was also negatively associated with PGS for PGP2 (FA2B).

Biochemically related groups of plasma N-glycosylation traits, associated with ICD-10 disease phenotypes

Galactosylation-related traits

PGS for the glycan traits describing abundances of galactosylated structures (G1total, Gntotal, FA2G1 (PGP4), FA2G1 (PGP5), FA2G1n, G1n) were negatively associated with obesity, lipoprotein metabolism disorders, primary hypertension and non-insulin dependent diabetes.

Sialylation-related traits

PGS for glycosylation traits related to presence of sialylated structures found on immunoglobulins (PGP37, FGS/(FG+FGS); PGP39, FGS/(F+FG+FGS)) were negatively correlated with primary hypertension and non-insulin dependent diabetes, while PGS for PGP94 (S0total, percentage of neutral glycan structures in total plasma glycans) related to absence of sialylation was negatively correlated with primary hypertension, lipidaemias (E78), obesity and non-insulin dependent diabetes (Fig. 4). PGS for relative abundances of trisialylated structures that are normally present on non-Ig plasma glycoproteins were positively correlated with disorders of lipoprotein metabolism and lipidemias (PGP49-51, all three traits describe ratios of various mono- and disialylated structures to trisialylated N-glycans) while PGS for antennary fucosylation of trisialylated N-glycans (PGP112, G3Fa/G3total) were negatively correlated with disorders of lipoprotein metabolism (E78). PGS for abundances of di- and trisialylated fully galactosylated triantennary structures were positively correlated with leiomyoma of uterus, non-insulin dependent diabetes mellitus, essential (primary) hypertension and gout, while PGS for two of such traits, namely, PGP22 (A3G3S2) and PGP27 (A3G3S3) were negatively associated with cholelithiasis (K80). PGS for traits describing lower levels of antennary sialylation (PGP47, PGP49-51) were negatively associated with disorders of lipoprotein metabolism and other lipidaemias.

Bisection-related traits

PGS for the traits related to bisection (PGP59, FA2Bn; PGP82, FBG1n/G1n; PGP84, FBn/Fn; PGP85, FBn/Fn total) were positively correlated with mental and behavioural disorders due to use of tobacco (F17) and negatively with disorders of lipoprotein metabolism (PGP108 - Btotal, PGP3 -A2BG1, Fig. 4).

H*igh-mannose-related traits*

PGS for relative abundances of several high-mannose structures were positively associated with seven diseases: asthma (PGP107, Mtotal; PGP18, M9; PG64, M6n; PGP69, M9n), chronic obstructive pulmonary disease (M9), essential (primary) hypertension (M9, M9n), angina pectoris (M9, M9n), chronic ischaemic heart disease (M9, M9n), disorders of lipoprotein metabolism (Mtotal; PGP114, M7n; M9; M9n), and non-insulin dependent diabetes (M9n).

Causal effect of plasma N-glycosylation traits on ICD-10 disease phenotypes

Effect of PGP64 on asthma

Two-sample MR analysis of the causal effect of PGP64 on asthma (J45) was based on three SNPs used as instrumental variables (IVs) that were associated with PGP64 at the designated level of statistical significance ($P \leq 1.79 \times 10^{-9}$). The statistically significant estimate of the causal effect in this case was obtained with the Weighted median method ($P = 9.78 \times 10^{-4}$, Table 2, Supplementary Table 11a). All five methods of Two-Sample MR implemented in the TwoSampleMR R package gave similar estimates of causal effect in the range of 0.010 - 0.019 log OR units of asthma (J45) per SD unit of PGP64 (Supplementary Table 12a, Supplementary Fig. 5a). No significant heterogeneity (Supplementary Table 12b) was observed between the IVs and the test for directional horizontal pleiotropy was also non-significant (Supplementary Table 12c). Leave-one-out sensitivity analysis (Supplementary Figure 5b) and the forest plot suggested that no single IV was driving the observed causal effect (Supplementary Figure 5c). Although the funnel plot for the three available IVs is not very informative, no obvious asymmetry suggesting horizontal pleiotropy was observed (Supplementary Figure 5d).

As additional sensitivity analysis we performed colocalization (SMR-HEIDI) analysis for the effect of PGP64 on Asthma (J45) for the three loci used as IVs in the two-sample MR to test whether these associations are due to the same underlying causal variant or if they are due to linkage disequilibrium between two causal variants (Supplementary Table 13).

We found evidence for the shared causal variant between PGP64 and Asthma (J45) (P SMR = 0.0032; P HEIDI = 0.30) at the locus tagged by rs144126567. Evidence for linkage (P HEIDI <= 0.05) was observed for the loci tagged by rs440674. Therefore, sensitivity analysis confirmed the observed causal effect for PGP64 (The percentage of M6 in total neutral plasma glycans) on asthma for at least one locus.

Effect of PGP69 on disorders of lipoprotein metabolism and other lipidaemias

Two-sample MR analysis of the causal effect of PGP69 on disorders of lipoprotein metabolism and other lipidaemias (E78) was based on two IVs. Therefore, only IVW estimate of causal effect was available that produced a statistically significant estimate of such effect, equal to 0.013 log OR units of E78 per 1 SD unit of PGP69 ($P = 1.25 \times 10^{-4}$, Table 2, Supplementary Table 11a, Supplementary Figure 6a). No significant heterogeneity was observed between the IVs (Supplementary Table 12b). Due to the low number of IVs we could not conduct the leave-one-out sensitivity analysis or test for the presence of directional horizontal pleiotropy and the funnel plot was not informative enough (Supplementary Figure 6b). Forest plot suggests that both IVs give similar estimates to the overall one and the final estimate of the causal effect is not driven by a single IV (Supplementary Figure 6c). Additional SMR-HEIDI analysis for the causal effect of

PGP69 on disorders of lipoprotein metabolism and other lipidaemias revealed evidence for linkage (*P* HEIDI <= 0.05) for the locus tagged by rs401775. SMR-HEIDI analysis did not provide any proof of the causal effect of PGP64 on E78 (Supplementary Table 13).

Causal effect of ICD-10 disease phenotypes on plasma N-glycosylation traits

We found statistically significant effects of disorders of lipoprotein metabolism and other lipidaemias (E78) phenotype on PGP18 and on PGP107 using the same 27 genetic variants associated with E78 as IVs in both cases.

Effect of disorders of lipoprotein metabolism and other lipidaemias (E78) on PGP18

The statistically significant estimate of the causal effect of E78 on PGP18 was obtained with the IVW method and was equal to 4.09 SD units of PGP18 per 1 log OR unit of E78, with a corresponding p-value = 5.16×10^{-4} (Table 2). Different two-sample MR methods produced estimates that varied in magnitude, from 0.53 (Weighted Mode, *P* = 0.746) to 3.62 (MR Egger, *P* = 0.143) SD units of PGP18 per 1 log OR unit of E78 (Supplementary Table 12a, Supplementary Fig. 7a). Both IVW and MR Egger tests indicated presence of heterogeneity between the IVs (Supplementary Table 12b). The test for directional horizontal pleiotropy was non-significant (Supplementary Table 12c). Leave-one-out sensitivity analysis (Supplementary Fig. 7b) and the forest plot (Supplementary Fig. 7c) suggested that no single IV was driving the observed causal effect, the funnel plot was asymmetric with an excess of IVs with strong positive effect on the abundance of PGP18 (Supplementary Fig. 7d).

Effect of disorders of lipoprotein metabolism and other lipidaemias (E78) on PGP107

Two-sample MR estimates of causal effect of E78 on PGP107 ranged from 1.25 (MR Egger, *P* = 0.587) to 3.820 (Simple mode, *P* = 0.031) SD units of PGP18 per 1 log OR unit of E78 (Supplementary Table 12a, Supplementary Fig. 8a). However, only Weighted median provided a causal effect estimate that was statistically significant at our designated threshold: bcausal = 3.01 SD units of PGP18 per 1 log OR unit of E78 with a *P* of 8.16 x 10-4 (Table 2-3). Both IVW and MR Egger tests did not indicate presence of heterogeneity between the IVs (Supplementary Table 12b). The test for directional horizontal pleiotropy was non-significant (Supplementary Table 12c). Leave-one-out sensitivity analysis suggested that no single IV was driving the observed causal effect (Supplementary Figure 8b). The funnel plot is asymmetric with an excess of IVs with strong positive effect on the abundance of PGP107 (Supplementary Figure 8c) and the same can be observed in the forest plot (Supplementary Figure 8d).

Sensitivity analyses

As additional sensitivity analysis for the causal effects of disorders of lipoprotein metabolism and other lipidaemias (E78) on plasma N-glycosylation traits PGP18 and PGP107 we used the MR-PRESSO R package[73] to identify and remove the pleiotropic IVs from analysis. We

used a strict criterium for IV exclusion, removing the SNPS for which *P*-values of the test for outliers in MR-PRESSO were < 1 and performed another MR analysis without these SNPs.

Effect of disorders of lipoprotein metabolism and other lipidaemias (E78) on PGP18

For MR analysis of the causal effect of E78 on PGP18 we removed eight outlier IVs and performed two-sample MR on a filtered set of 19 IVs. Five used methods produced estimates that varied from 3.39 to 4.82 SD units of PGP18 per log OR unit of E78 (Supplementary Table 14a, Supplementary Fig. 9a). The lowest p-value corresponded to the IVW estimate of causal effect equal to 3.30 SD units of PGP18 per log OR unit of Disorders of lipoprotein metabolism (p-value = 6.19 x 10-5, Supplementary Table 14a). After removal of outliers the five MR methods started to agree more in the estimate of causal effect and the statistical significance of the estimates improved. No significant heterogeneity between the IVs was detected and the test for directional horizontal pleiotropy was non-significant (Supplementary Tables 14b,c). Leave-one-out sensitivity analysis (Supplementary Fig. 9b) and the forest plot (Supplementary Fig. 9c) suggested that no single IV was driving the observed causal effect. The funnel plot after removal of outliers looks more symmetric (Supplementary Fig. 9d).

Effect of disorders of lipoprotein metabolism and other lipidaemias (E78) on PGP107

When we were performing additional sensitivity analysis for the causal effect of E78 on PGP107 we removed four outlier IVs and performed two Sample MR on a filtered set of 23 IVs. Five used methods produced estimates that varied from 2.09 to 4.16 SD units of PGP107 per log OR unit of E78 (Supplementary Table 14a). The lowest p-value corresponded to the Weighted median estimate of causal effect equal to 3.13 SD units of PGP107 per log OR unit of E78 (p-value = 6.74 x 10-5, Supplementary Table 14a, Supplementary Fig. 10a). No significant heterogeneity between the IVs was detected (Supplementary Table 14b) and the test for directional horizontal pleiotropy was non-significant (Supplementary Table 14c). Leave-one-out sensitivity analysis (Supplementary Fig. 10b) and the forest plot (Supplementary Fig. 10c) suggested that no single IV was driving the observed causal effect. The funnel plot after removal of outliers looks more symmetric (Supplementary Fig. 10d).

The results of the sensitivity analyses are collinear with the first round of two-sample MR analysis for the effect of E78 disease phenotype on PGP18 and PGP107 and suggest the presence of the positive causal effect of Disorders of lipoprotein metabolism and other lipidaemias (E78) on PGP18 and on PGP107 plasma N-glycosylation traits.

Tissue-specificity of eQTLs

To evaluate the tissue specificity of eQTLs, we selected two of the most relevant tissue from the GTEx study[74] – EBV-transformed B lymphocytes and liver cells – and examined the number of genes that have either a common variant or variants in high LD ($R^2 > 0.8$) in these two tissues. We utilized 1000 Genomes EUR data to calculate the LD matrix between SNPs within each gene.

MR between N-glycans and liver enzymes and NAFLD

To further explore the genetic link between blood plasma N-glycosylation and markers of liver physiology we performed bidirectional Mendelian randomization (MR) analyses. Specifically, we investigated causal relationships between all measured N-glycan levels and non-alcoholic fatty liver disease (NAFLD) —a common chronic liver disease—as well as four clinically relevant liver biomarkers: blood levels of alanine aminotransferase (ALT), alkaline phosphatase (ALP), gamma-glutamyl transferase (GGT), and aspartate aminotransferase (AST), which reflect distinct aspects of liver physiology and health.

Our MR results demonstrate that increased levels of glycans PGP25 and PGP27 (both possessing the tri-sialylated, tri-antennary A3G3S3 structure), predominantly present on alpha-1-acid glycoprotein, alpha-1-antitrypsin, and beta-2-glycoprotein 1 (primarily hepatocyte-derived proteins) [https://doi.org/10.1016/j.eng.2023.03.013], are significantly associated with lower AST levels. These glycoproteins play critical roles in modulating systemic inflammation, oxidative stress, and immune responses, processes directly relevant to liver injury and repair [https://doi.org/10.4254/wjh.v13.i11.1688]. Therefore, the association between increased A3G3S3 glycan levels and lower AST might reflect a protective effect against hepatocellular damage or stress.

However, we did not observe significant causal associations for ALT, ALP, GGT, or NAFLD. This lack of significant findings may result partly from limited statistical power due to the small number of genetic instrumental variables available for glycan traits (3–8 instruments per trait). Alternatively, there might be a specific biological effect linking these glycans to AST levels, but not necessarily to other liver enzymes or NAFLD. Consequently, the absence of significant findings for these other liver phenotypes should be interpreted with caution. While our data supports a genetic association between the A3G3S3 glycan structure and AST, further studies with enhanced statistical power are needed to clarify potential implications for liver health.

References

1. Moayyeri, A., Hammond, C. J., Hart, D. J. & Spector, T. D. The UK Adult Twin Registry (TwinsUK Resource). *Twin Research and Human Genetics* **16**, 144–149 (2013).
2. Boeing, H., Korfmann, A. & Bergmann, M. M. Recruitment Procedures of EPIC-Germany. *Annals of Nutrition and Metabolism* **43**, 205–215 (1999).
3. Boeing, H., Wahrendorf, J. & Becker, N. EPIC-Germany – A Source for Studies into Diet and Risk of Chronic Diseases. *Annals of Nutrition and Metabolism* **43**, 195–204 (1999).
4. Bergmann, M. M., Bussas, U. & Boeing, H. Follow-Up Procedures in EPIC-Germany – Data Quality Aspects. *Annals of Nutrition and Metabolism* **43**, 225–234 (1999).

5. Allegri, M. *et al.* 'Omics' biomarkers associated with chronic low back pain: protocol of a retrospective longitudinal study. *BMJ Open* **6**, e012070 (2016).
6. Dagostino, C. *et al.* Validation of standard operating procedures in a multicenter retrospective study to identify -omics biomarkers for chronic low back pain. *PLOS ONE* **12**, e0176372 (2017).
7. Vuckovic, F. *et al.* IgG Glycome in Colorectal Cancer. *Clinical Cancer Research* **22**, 3078–3086 (2016).
8. Theodoratou, E. *et al.* Glycosylation of plasma IgG in colorectal cancer prognosis. *Scientific Reports* **6**, 28098 (2016).
9. Tillin, T., Forouhi, N. G., McKeigue, P. M., Chaturvedi, N. & SABRE Study Group. Southall And Brent REvisited: Cohort profile of SABRE, a UK population-based comparison of cardiovascular disease and diabetes in people of European, Indian Asian and African Caribbean origins. *International journal of epidemiology* **41**, 33–42 (2012).
10. Mook-Kanamori, D. O. *et al.* 1,5-Anhydroglucitol in Saliva Is a Noninvasive Marker of Short-Term Glycemic Control. *The Journal of Clinical Endocrinology & Metabolism* **99**, E479–E483 (2014).
11. Suhre, K. *et al.* Connecting genetic risk to disease end points through the human blood plasma proteome. *Nature Communications* **8**, 14357 (2017).
12. Momozawa, Y. *et al.* IBD risk loci are enriched in multigenic regulatory modules encompassing putative causative genes. *Nature Communications* **9**, 2427 (2018).
13. Anderson, C. A. *et al.* Data quality control in genetic case-control association studies. *Nature Protocols* **5**, 1564–1573 (2010).
14. Langenberg, C. *et al.* Gene-Lifestyle Interaction and Type 2 Diabetes: The EPIC InterAct Case-Cohort Study. *PLoS Medicine* **11**, (2014).
15. Grove, M. L. *et al.* Best practices and joint calling of the HumanExome BeadChip: the CHARGE Consortium. *PloS one* **8**, e68095 (2013).

16. Guo, Y. *et al.* Illumina human exome genotyping array clustering and quality control. *Nature Protocols* **9**, 2643–2662 (2014).

17. Goldstein, J. I. *et al.* zCall: a rare variant caller for array-based genotyping: genetics and population analysis. *Bioinformatics* **28**, 2543–5 (2012).

18. Das, S. *et al.* Next-generation genotype imputation service and methods. *Nature Genetics* **48**, 1284–1287 (2016).

19. McCarthy, S. *et al.* A reference panel of 64,976 haplotypes for genotype imputation. *Nature Genetics* **48**, 1279–1283 (2016).

20. Loh, P. R., Palamara, P. F. & Price, A. L. Fast and accurate long-range phasing in a UK Biobank cohort. *Nature Genetics* **48**, 811–816 (2016).

21. Tenesa, A. *et al.* Genome-wide association scan identifies a colorectal cancer susceptibility locus on 11q23 and replicates risk loci at 8q24 and 18q21. *Nature Genetics* **40**, 631–637 (2008).

22. Law, P. J. *et al.* Association analyses identify 31 new risk loci for colorectal cancer susceptibility. *Nature Communications* **10**, 2154 (2019).

23. Trbojević Akmačić, I. *et al.* High-throughput glycomics: Optimization of sample preparation. *Biochemistry (Moscow)* **80**, 934–942 (2015).

24. Agakova, A., Vučković, F., Klarić, L., Lauc, G. & Agakov, F. Automated Integration of a UPLC Glycomic Profile. *Methods in Molecular Biology* **1503**, 217–233 (2017).

25. Sharapov, S. Z. *et al.* Defining the genetic control of human blood plasma N-glycome using genome-wide association study. *Human Molecular Genetics* **28**, 2062–2077 (2019).

26. Sharapov, S. Z. *et al.* Replication of 15 loci involved in human plasma protein N-glycosylation in 4802 samples from four cohorts. *Glycobiology* **31**, 82–88 (2021).

27. Dieterle, F., Ross, A., Schlotterbeck, G. & Senn, H. Probabilistic Quotient Normalization as Robust Method to Account for Dilution of Complex Biological Mixtures. Application in 1 H NMR Metabonomics. *Analytical Chemistry* **78**, 4281–4290 (2006).

28. Benedetti, E. *et al.* Systematic evaluation of normalization methods for glycomics data based on performance of network inference. *bioRxiv* 814244 (2019) doi:10.1101/814244.

29. Johnson, W. E., Li, C. & Rabinovic, A. Adjusting batch effects in microarray expression data using empirical Bayes methods. *Biostatistics* **8**, 118–127 (2007).

30. Winkler, T. W. *et al.* Quality control and conduct of genome-wide association meta-analyses. *Nature Protocols* **9**, 1192–1212 (2014).

31. Aulchenko, Y. S., Ripke, S., Isaacs, A. & van Duijn, C. M. GenABEL: an R library for genome-wide association analysis. *Bioinformatics* **23**, 1294–1296 (2007).

32. Karssen, L. C., van Duijn, C. M. & Aulchenko, Y. S. The GenABEL Project for statistical genomics. *F1000Research* **5**, 914 (2016).

33. Shashkova, T. I. *et al.* The GWAS-MAP platform for aggregation of results of genome-wide association studies and the GWAS-MAP|homo database of 70 billion genetic associations of human traits. *Vavilov Journal of Genetics and Breeding* **24**, 876–884 (2020).

34. Shen, X. *et al.* Multivariate discovery and replication of five novel loci associated with Immunoglobulin G N-glycosylation. *Nat Commun* **8**, 447 (2017).

35. Shadrina, A. S. *et al.* Multivariate genome-wide analysis of immunoglobulin G N-glycosylation identifies new loci pleiotropic with immune function. *Hum Mol Genet* **30**, 1259–1270 (2021).

36. Ning, Z. *et al.* Nontrivial Replication of Loci Detected by Multi-Trait Methods. *Front Genet* **12**, 627989 (2021).

37. NCI-NHGRI Working Group on Replication in Association Studies *et al.* Replicating genotype–phenotype associations. *Nature* **447**, 655–660 (2007).

38. Clerc, F. *et al.* Human plasma protein N-glycosylation. *Glycoconj J* **33**, 309–343 (2016).

39. Yamamoto, F., Clausen, H., White, T., Marken, J. & Hakomori, S. Molecular genetic basis of the histo-blood group ABO system. *Nature* **345**, 229–233 (1990).

40. McLaren, W. *et al.* The Ensembl Variant Effect Predictor. *Genome Biol* **17**, 122 (2016).

41. Rogers, M. *et al.* FATHMM-XF: accurate prediction of pathogenic point mutations via extended features. *Bioinform.* (2018) doi:10.1093/bioinformatics/btx536.
42. Ferlaino, M. *et al.* An integrative approach to predicting the functional effects of small indels in non-coding regions of the human genome. *BMC Bioinformatics* **18**, 442 (2017).
43. Uchimura, K. *et al.* Human N-acetylglucosamine-6-O-sulfotransferase involved in the biosynthesis of 6-sulfo sialyl Lewis X: molecular cloning, chromosomal mapping, and expression in various organs and tumor cells. *J Biochem* **124**, 670–678 (1998).
44. Seixas, S. & Marques, P. I. Known Mutations at the Cause of Alpha-1 Antitrypsin Deficiency an Updated Overview of SERPINA1 Variation Spectrum. *The Application of Clinical Genetics* **14**, 173–194 (2021).
45. Alpha-1 antitrypsin deficiency: A conformational disease associated with lung and liver manifestations - Greene - 2008 - Journal of Inherited Metabolic Disease - Wiley Online Library. https://onlinelibrary.wiley.com/doi/10.1007/s10545-007-0748-y.
46. Balcar, L. *et al.* Alpha-1 antitrypsin Pi∗Z allele is an independent risk factor for liver transplantation and death in patients with advanced chronic liver disease. *JHEP Reports* **4**, 100562 (2022).
47. Kettunen, J. *et al.* Genome-wide study for circulating metabolites identifies 62 loci and reveals novel systemic effects of LPA. *Nat Commun* **7**, 11122 (2016).
48. Mijakovac, A. *et al.* Effects of Estradiol on Immunoglobulin G Glycosylation: Mapping of the Downstream Signaling Mechanism. *Frontiers in Immunology* **12**, (2021).
49. Klarić, L. *et al.* Glycosylation of immunoglobulin G is regulated by a large network of genes pleiotropic with inflammatory diseases. *Science Advances* **6**, eaax0301 (2020).
50. Lauc, G. *et al.* Genomics Meets Glycomics—The First GWAS Study of Human N-Glycome Identifies HNF1α as a Master Regulator of Plasma Protein Fucosylation. *PLoS Genetics* **6**, e1001256 (2010).

51. Martincic, K., Alkan, S. A., Cheatle, A., Borghesi, L. & Milcarek, C. Transcription elongation factor ELL2 directs immunoglobulin secretion in plasma cells by stimulating altered RNA processing. *Nature Immunology* **10**, 1102–1109 (2009).
52. Riley, L. G. *et al.* A SLC39A8 variant causes manganese deficiency, and glycosylation and mitochondrial disorders. *J Inherit Metab Dis* **40**, 261–269 (2017).
53. Park, J. H. *et al.* SLC39A8 Deficiency: A Disorder of Manganese Transport and Glycosylation. *Am J Hum Genet* **97**, 894–903 (2015).
54. Ushioda, R. & Nagata, K. The endoplasmic reticulum-associated degradation and disulfide reductase ERdj5. *Methods Enzymol* **490**, 235–258 (2011).
55. Roxrud, I., Raiborg, C., Gilfillan, G. D., Strømme, P. & Stenmark, H. Dual degradation mechanisms ensure disposal of NHE6 mutant protein associated with neurological disease. *Exp Cell Res* **315**, 3014–3027 (2009).
56. Rivinoja, A., Hassinen, A., Kokkonen, N., Kauppila, A. & Kellokumpu, S. Elevated Golgi pH impairs terminal N-glycosylation by inducing mislocalization of Golgi glycosyltransferases. *J. Cell. Physiol.* **220**, 144–154 (2009).
57. Robbins, A. L. & Savage, D. B. The genetics of lipid storage and human lipodystrophies. *Trends Mol Med* **21**, 433–438 (2015).
58. Xu, X., So, J.-S., Park, J.-G. & Lee, A.-H. Transcriptional control of hepatic lipid metabolism by SREBP and ChREBP. *Semin Liver Dis* **33**, 301–311 (2013).
59. Baklaushev, V. P. *et al.* Luciferase Expression Allows Bioluminescence Imaging But Imposes Limitations on the Orthotopic Mouse (4T1) Model of Breast Cancer. *Sci Rep* **7**, 7715 (2017).
60. Soubeyrand, S., Martinuk, A., Naing, T., Lau, P. & McPherson, R. Role of Tribbles Pseudokinase 1 (TRIB1) in human hepatocyte metabolism. *Biochim Biophys Acta* **1862**, 223–232 (2016).
61. Liver fat storage is controlled by HNF4α through induction of lipophagy and is reversed by a potent HNF4α agonist - PubMed. https://pubmed.ncbi.nlm.nih.gov/34117215/.

62. TRIB1 downregulates hepatic lipogenesis and glycogenesis via multiple molecular interactions - PubMed. https://pubmed.ncbi.nlm.nih.gov/24389359/.

63. Zimmermann, E. *et al.* C-reactive protein levels in relation to various features of non-alcoholic fatty liver disease among obese patients. *Journal of hepatology* **55**, 660–665 (2011).

64. Orho-Melander, M. *et al.* Common missense variant in the glucokinase regulatory protein gene is associated with increased plasma triglyceride and C-reactive protein but lower fasting glucose concentrations. *Diabetes* **57**, 3112–3121 (2008).

65. Dehghan, A. *et al.* Meta-analysis of genome-wide association studies in >80 000 subjects identifies multiple loci for C-reactive protein levels. *Circulation* **123**, 731–738 (2011).

66. Pontoglio, M. *et al.* Hepatocyte nuclear factor 1 inactivation results in hepatic dysfunction, phenylketonuria, and renal Fanconi syndrome. *Cell* **84**, 575–585 (1996).

67. Lee, Y. H., Sauer, B. & Gonzalez, F. J. Laron dwarfism and non-insulin-dependent diabetes mellitus in the Hnf-1alpha knockout mouse. *Mol Cell Biol* **18**, 3059–3068 (1998).

68. Hua, M.-C. *et al.* Alternation of plasma fatty acids composition and desaturase activities in children with liver steatosis. *PLoS One* **12**, e0182277 (2017).

69. A genome-wide association study for nonalcoholic fatty liver disease identifies novel genetic loci and trait-relevant candidate genes in the Million Veteran Program | medRxiv. https://www.medrxiv.org/content/10.1101/2020.12.26.20248491v1.

70. Wang, L. *et al.* Fatty acid desaturase 1 gene polymorphisms control human hepatic lipid composition. *Hepatology* **61**, 119–128 (2015).

71. Czumaj, A. & Śledziński, T. Biological Role of Unsaturated Fatty Acid Desaturases in Health and Disease. *Nutrients* **12**, 356 (2020).

72. Stoffel, W. *et al.* Obesity resistance and deregulation of lipogenesis in Δ6-fatty acid desaturase (FADS2) deficiency. *EMBO Rep* **15**, 110–120 (2014).

73. Verbanck, M., Chen, C.-Y., Neale, B. & Do, R. Detection of widespread horizontal pleiotropy in causal relationships inferred from Mendelian randomization between complex traits and diseases. *Nat Genet* **50**, 693–698 (2018).

74. The GTEx Consortium *et al.* The GTEx Consortium atlas of genetic regulatory effects across human tissues. *Science* **369**, 1318–1330 (2020).
