# Supplementary material for: A genome-wide association study in 10,000 individuals links plasma N-glycome to liver disease and anti-inflammatory proteins
Source: Nat Commun. 2025 Jul 1;16:5525. doi: 10.1038/s41467-025-60431-y (PMC12218978; doi:10.1038/s41467-025-60431-y)
Supplement: Supplementary file 2 — Description of Additional Supplementary Files [file 41467_2025_60431_MOESM2_ESM.pdf]

Supplementary Data 1
1a. Description of total plasma protein N-glycosylation traits
1b. Description of total plasma protein N-glycosylation multivariate traits

Supplementary Data 2
2a. Description of the cohorts and design
2b. Demographic descriptive statistics of discovery and replication cohorts
2c. Details of the genotyping, imputation and association analysis

Supplementary Data 3
3a. Loci associated with total plasma N-Glycome traits in the univariate analysis
3b. Loci associated with total plasma N-Glycome traits in the multivariate analysis

Supplementary Data 4
4. Phenotypic variance explained by significantly associated SNPs identified by CoJo (conditional & joint association analysis using GWAS summary statistics) (p<=5e-8/28).

Supplementary Data 5
5a. Results of LDSC regression on discovery meta-analysis (7540)
5b. Results of LDSC regression on EUR meta-analysis (10172)
5c. Genomic control results for discovery meta-analysis and for multivariate analysis

Supplementary Data 6
6. SBayesR whole genome prediction models and SNP-based heritability for 117 N-glycome traits

Supplementary Data 7
7a. Summary of in silico post-GWAS follow up for gene and variant prioritization
7b. Predicted functional consequences of single nucleotides variants (FATHMM-XF)
7c. Predicted functional consequences of small indels (FATHMM-indel)
7d. Predicted functional consequences of single nucleotides variants (VEP)
7e. Summary of the SMR/HEIDI analysis for pleiotropy with with RNA level gene expression
7f. Summary of the SMR/HEIDI analysis for pleiotropy with protein expression level
7g. Results of gene prioritization analysis by DEPICT
7h. Results of gene set enrichment analysis by DEPICT
7i. Results of tissue enrichment by DEPICT
7j. Gene descriptions
7k. Prioritized variants

Supplementary Data 8
8a. Loci associated with total plasma N-Glycome traits that were found and replicated in previous works
8b. Loci associated with transferrin and immunoglobulin G N-Glycome traits that were found and replicated in works [1-3]
8c. Significant (p < 0.01/36 = 0.00028) association of the partial regression coefficients in the multivariate analysis of a trait set "N-glycosylation" (36 N-glycans)

Supplementary Data 9
9a. Secondary Traits used in the SMR/HEIDI analysis
9b. Summary of the SMR/HEIDI analysis for pleiotropy with complex traits

Supplementary Data 10
10. Association of ICD-10 disease phenotypes and PRS for plasma N-glycosylation traits

Supplementary Data 11
11a. MR analysis of causal effects of plasma N-glycosylation traits on disease phenotypes
11b. MR analysis of causal effects of disease phenotypes on plasma N-glycosylation traits.

Supplementary Data 12
12a. Sensitivity analyses for the significant causal effects of plasma N-glycosylation traits on ICD-10 disease phenotypes and vice versa.
12b. Heterogeneity tests for the significant causal effects of plasma N-glycosylation traits on ICD-10 disease phenotypes and vice versa.
12c. Test for directional horizontal pleiotropy for the significant causal effects of plasma N-glycosylation traits on ICD-10 disease phenotypes and vice versa.

Supplementary Data 13
13. SMR-HEIDI analysis for the effects of plasma N-glycosylation traits on diseases

Supplementary Data 14
14a. MR-PRESSO Sensitivity analysis for the effects of disorders of lipoprotein metabolism and other lipidaemias on plasma N-glycosylation traits
14b. Heterogeneity tests for the significant causal effects of plasma N-glycosylation traits on ICD-10 disease phenotypes estimated after removal of pleiotropic IVs
14c. Test for directional horizontal pleiotropy for the significant causal effects of ICD-10 disease phenotypes on plasma N-glycosylation traits estimated after removal of pleiotropic IVs

Supplementary Data 15
15. Description of genome-wide association studies providing summary statistics for ICD-10 disease phenotypes used for MR and SMR-HEIDI analyses in the current study

Supplementary Data 16
16a. Bidirectional Mendelian Randomization analysis for N-glycosylation traits and liver-associated traits.
16b. Bidirectional Mendelian Randomization analysis for N-glycosylation traits and pQTLs.
16c. SMR-HEIDI analysis for the effects of plasma N-glycosylation traits on liver enzymes.

Supplementary Data 17
17. Correspondence between UHPLC glycan peaks measured in the studied cohorts
