# Supplementary material for: A genome-wide association study in 10,000 individuals links plasma N-glycome to liver disease and anti-inflammatory proteins
Source: Nat Commun. 2025 Jul 1;16:5525. doi: 10.1038/s41467-025-60431-y (PMC12218978; doi:10.1038/s41467-025-60431-y)
Supplement: Supplementary file 20 — Reporting Summary [file 41467_2025_60431_MOESM20_ESM.pdf]

nature portfolio

Corresponding author(s): Sodbo Sharapov, Yurii S. Aulchenko

Last updated by author(s): April 15, 2025

Reporting Summary

Nature Portfolio wishes to improve the reproducibility of the work that we publish. This form provides structure for consistency and transparency in reporting. For further information on Nature Portfolio policies, see our Editorial Policies and the Editorial Policy Checklist.

Statistics

For all statistical analyses, confirm that the following items are present in the figure legend, table legend, main text, or Methods section.

n/a	Confirmed	
☐	☒	The exact sample size (*n*) for each experimental group/condition, given as a discrete number and unit of measurement
☐	☒	A statement on whether measurements were taken from distinct samples or whether the same sample was measured repeatedly
☐	☒	The statistical test(s) used AND whether they are one- or two-sided<br>*Only common tests should be described solely by name; describe more complex techniques in the Methods section.*
☐	☒	A description of all covariates tested
☐	☒	A description of any assumptions or corrections, such as tests of normality and adjustment for multiple comparisons
☐	☒	A full description of the statistical parameters including central tendency (e.g. means) or other basic estimates (e.g. regression coefficient) AND variation (e.g. standard deviation) or associated estimates of uncertainty (e.g. confidence intervals)
☐	☒	For null hypothesis testing, the test statistic (e.g. *F*, *t*, *r*) with confidence intervals, effect sizes, degrees of freedom and *P* value noted<br>*Give P values as exact values whenever suitable.*
☐	☒	For Bayesian analysis, information on the choice of priors and Markov chain Monte Carlo settings
☒	☐	For hierarchical and complex designs, identification of the appropriate level for tests and full reporting of outcomes
☐	☒	Estimates of effect sizes (e.g. Cohen's *d*, Pearson's *r*), indicating how they were calculated

Our web collection on statistics for biologists contains articles on many of the points above.

Software and code

Policy information about availability of computer code

Data collection	No software was used to collect the data.
Data analysis	Quality control analyses, meta-analyses, clumping, colocalisation analyses, Mendelian randomisation were performed using the GWAS-MAP platform [10.18699/VJ20.686]. R v.4.2.0 (General statistics); GCTA-COJO v1.93.2beta (conditional analysis); SBayesR vgctb_2.03 (PGS models); locuszoom v0.12, PLINK 1.90 v2021-05-28, VEP v113, FATHMM v2.3, DEPICT v1 rel194 (post-gwas analyses)

For manuscripts utilizing custom algorithms or software that are central to the research but not yet described in published literature, software must be made available to editors and reviewers. We strongly encourage code deposition in a community repository (e.g. GitHub). See the Nature Portfolio guidelines for submitting code & software for further information.

Data

Policy information about availability of data

All manuscripts must include a data availability statement. This statement should provide the following information, where applicable:

- Accession codes, unique identifiers, or web links for publicly available datasets
- A description of any restrictions on data availability
- For clinical datasets or third party data, please ensure that the statement adheres to our policy

The full genome-wide summary association statistics for 117 glycome traits from the European GWAMA (Genome-Wide Association Meta-Analysis) conducted on

participants of European ancestry, totaling 10,172 individuals, have been deposited in the Zenodo database under accession code 15057709 (CC BY 4.0) (https://doi.org/10.5281/zenodo.15057709).

The full genome-wide summary association statistics for 117 glycome traits from the discovery GWAMA (Genome-Wide Association Meta-Analysis) conducted on participants, totaling 7,540 individuals, have been deposited in the Zenodo database under accession code 15150419 (CC BY 4.0) (https://doi.org/10.5281/zenodo.15150419).

The first part of the full genome-wide summary association statistics for 117 glycome traits from the replication GWAMA (Genome-Wide Association Meta-Analysis) conducted on participants, totaling 3,224 individuals, have been deposited in the Zenodo database under accession code 15161945 (CC BY 4.0) (https://doi.org/10.5281/zenodo.15161945).

The second part of the full genome-wide summary association statistics for 117 glycome traits from the replication GWAMA (Genome-Wide Association Meta-Analysis) conducted on participants, totaling 3,224 individuals, have been deposited in the Zenodo database under accession code 15166735 (CC BY 4.0) (https://doi.org/10.5281/zenodo.15166735).

The data generated in the secondary analyses of this study are included with this article in the Supplementary Data.

Summary statistics for IgG N-Glycosylation (DOI: 10.1126/sciadv.aax0301), TF N-Glycosylation (DOI: 10.1038/s41467-022-29189-5); summary statistics for complex and medical conditions-related traits were obtained from the UK Biobank (DOI: 10.1038/s41588-018-0248-z), the CARDIoGRAM Consortium (http://www.cardiogramplusc4d.org/), the Psychiatric Genomics consortium (https://pgc.unc.edu/) and other trait collections from other studies (see Supplementary Table 9 for the full list of the traits analyzed). All data is contained in GWAS-MAP platform [doi: 10.18699/VJGB-22-46].

Research involving human participants, their data, or biological material

Policy information about studies with human participants or human data. See also policy information about sex, gender (identity/presentation), and sexual orientation and race, ethnicity and racism.

Reporting on sex and gender	Data on sex was collected and used as a covariate in GWAS analyses. Sex and gender-based analyses were not performed.
Reporting on race, ethnicity, or other socially relevant groupings	Participants to the meta-analysis were all of European Ancestry except for SABRE cohort (South Asian descent), QMDIAB (Arab, South Asian and Filipino descent).
Population characteristics	Participants to the meta-analysis were all of European Ancestry except for SABRE cohort (South Asian descent), QMDIAB (Arab, South Asian and Filipino descent).
Recruitment	Participants were recruited differently across studies that took part to the meta-analysis, see the following PMID: [TwinsUK: 23088889], [SABRE: 21044979], [SOCCS: 18199722,19011631,22634755], [QMDiab: 24423354, 25434815, 26049400], [Painomics: 28459826], [CEDAR: 29930244]
Ethics oversight	TwinsUK: The TwinsUK Study was approved by London-Westminster Research Ethics Committee (REC reference EC04/015), and Guy's and St Thomas' NHS Foundation Trust Research and Development (R&D). The TwinsUK BioBank was approved by the HRA - Liverpool East Research Ethics Committee (REC reference 19/NW/0187), IRAS ID 258513. All participants provide written, informed consent. EPIC-Potsdam: All participants gave written informed consent for biomedical research, and the study was approved by the Ethics Committee of the State of Brandenburg, Germany. PainOmics: The study was firstly approved by the Institutional Review Boards of IRCCS Foundation San Matteo Hospital Pavia and then by the Institutional Review boards of all clinical centers (King's College London, ZOL Genk/Lanaken, St. Catherine Specialty Hospital) that enrolled patients. Copies of approvals were provided to the European Commission before starting the study. Written informed consent was obtained from all participants. SOCCS: All participants gave written informed consent and study approval was from the MultiCentre Research Ethics Committee for Scotland and Local Research Ethics committee. SABRE: All participants gave written informed consent. Approval for the baseline study was obtained from Ealing, Hounslow, and Spelthorne, Parkside and University College London research ethnics committees. QMDiab: The initial study was approved by the Institutional Review Boards of HMC and Weill Cornell Medicine-Qatar (WCM-Q) (research protocol #11131/11). Written informed consent was obtained from all participants. CEDAR: The experimental protocol was approved by the ethics committee of the University of Liege Academic Hospital.

Note that full information on the approval of the study protocol must also be provided in the manuscript.

Field-specific reporting

Please select the one below that is the best fit for your research. If you are not sure, read the appropriate sections before making your selection.

☒ Life sciences ☐ Behavioural & social sciences ☐ Ecological, evolutionary & environmental sciences

For a reference copy of the document with all sections, see nature.com/documents/nr-reporting-summary-flat.pdf

Life sciences study design

All studies must disclose on these points even when the disclosure is negative.

Sample size	We have not performed sample size calculation as there is no known lower bound of glyQTL effect. However, we calculated the sample size of discovery and replication groups to achieve 80% replication power given the discovery threshold of 5e-8/28 and 50 loci, which we expected to

discover. Expected amount of loci discovered where calculated given the sample size and amount of found loci in the previous round (10.1093/hmg/ddz054).

Data exclusions	Data points that did not pass the internal QC of each cohort were excluded.
Replication	This study included replication cohort (N=3224), to confirm the true-positive status of discovery analysis (N=7450). This allowed us to reach a priori 80% of replication power of true-positive signal.
Randomization	No group allocation was performed in this study. Sex and age were included as covariates.
Blinding	Investigators were not blinded to group allocation during data collection and/or analysis - this is not relevant in the case of GWAS, as participants are not allocated to any group.

Reporting for specific materials, systems and methods

We require information from authors about some types of materials, experimental systems and methods used in many studies. Here, indicate whether each material, system or method listed is relevant to your study. If you are not sure if a list item applies to your research, read the appropriate section before selecting a response.

Materials & experimental systems

n/a	Involved in the study
☒	☐ Antibodies
☒	☐ Eukaryotic cell lines
☒	☐ Palaeontology and archaeology
☒	☐ Animals and other organisms
☒	☐ Clinical data
☒	☐ Dual use research of concern
☒	☐ Plants

Methods

n/a	Involved in the study
☒	☐ ChIP-seq
☒	☐ Flow cytometry
☒	☐ MRI-based neuroimaging

Plants

Seed stocks	*Report on the source of all seed stocks or other plant material used. If applicable, state the seed stock centre and catalogue number. If plant specimens were collected from the field, describe the collection location, date and sampling procedures.*
Novel plant genotypes	*Describe the methods by which all novel plant genotypes were produced. This includes those generated by transgenic approaches, gene editing, chemical/radiation-based mutagenesis and hybridization. For transgenic lines, describe the transformation method, the number of independent lines analyzed and the generation upon which experiments were performed. For gene-edited lines, describe the editor used, the endogenous sequence targeted for editing, the targeting guide RNA sequence (if applicable) and how the editor was applied.*
Authentication	*Describe any authentication procedures for each seed stock used or novel genotype generated. Describe any experiments used to assess the effect of a mutation and, where applicable, how potential secondary effects (e.g. second site T-DNA insertions, mosiacism, off-target gene editing) were examined.*
